# Peer mentorship to improve self-management of hip and knee osteoarthritis: a randomised feasibility trial

**DOI:** 10.1136/bmjopen-2020-045389

**Published:** 2021-07-21

**Authors:** Anna M Anderson, Elizabeth C Lavender, Esther Dusabe-Richards, Teumzghi F Mebrahtu, Linda McGowan, Philip G Conaghan, Sarah R Kingsbury, Gerry Richardson, Deborah Antcliff, Gretl A McHugh

**Affiliations:** 1 School of Healthcare, University of Leeds, Leeds, UK; 2 Leeds Institute of Rheumatic and Musculoskeletal Medicine and NIHR Leeds Biomedical Research Centre, University of Leeds, Leeds, UK; 3 Centre for Health Economics, University of York, York, UK; 4 NIHR Research Design Service for Yorkshire and the Humber, York, UK; 5 Physiotherapy Department, Bury Care Organisation, Northern Care Alliance NHS Group, Bury, UK

**Keywords:** rheumatology, pain management, social medicine

## Abstract

**Objective:**

To determine the feasibility of conducting a randomised controlled trial (RCT) of a peer mentorship intervention to improve self-management of osteoarthritis (OA).

**Design:**

Six-month parallel group non-blinded randomised feasibility trial.

**Setting:**

One secondary care and one primary care UK National Health Service Trust.

**Participants:**

Fifty adults aged ≥55 years old with hip and/or knee OA.

**Interventions:**

Participants were allocated 1:1 to the intervention or control group using an online randomisation service. Intervention group participants received usual care (information resources) and up to eight community-based self-management support sessions delivered by a peer mentor (trained volunteer with hip and/or knee OA). Control group participants received usual care only.

**Outcome measures:**

Key feasibility outcomes were participant and peer mentor recruitment and attrition, intervention completion and the sample size required for a definitive RCT. Based on these feasibility outcomes, four success criteria for proceeding to a definitive RCT were prespecified. Patient-reported outcomes were collected via questionnaires at baseline, 8 weeks and 6 months.

**Results:**

Ninety-six individuals were screened, 65 were eligible and 50 were randomised (25 per group). Of the 24 participants who commenced the intervention, 20 completed it. Four participants did not complete the 6-month questionnaire. Twenty-one individuals were eligible for the peer mentor role, 15 were trained and 5 withdrew prior to being matched with a participant. No intervention-related harms occurred. Allowing for 20% attrition, the sample size required for a definitive RCT was calculated as 170 participants. The intervention group showed improvements in self-management compared with the control group.

**Conclusions:**

The feasibility outcomes achieved the prespecified criteria for proceeding to an RCT. The exploratory analyses suggest peer mentorship may improve OA self-management. An RCT of the OA peer mentorship intervention is therefore warranted with minor modifications to the intervention and trial procedures.

**Trial registration number:**

ISRCTN:50675542.

**Strengths and limitations of this study**

- This randomised feasibility trial is the first study to develop a novel peer mentorship intervention specifically focused on improving self-management of hip and knee osteoarthritis (OA).
- Extensive patient and public involvement ensured the intervention and trial procedures were tailored to the needs of individuals with OA.
- A comprehensive range of feasibility outcomes were assessed, providing valuable information for designing a future definitive randomised controlled trial.
- Validated patient-reported outcome measures were administered; however, the results must be interpreted cautiously because the trial was not powered to detect statistically significant differences.
- A key limitation was that the majority of participants were recruited through a physiotherapy service and hence had already received some self-management support.

## Introduction

Osteoarthritis (OA) is one of the most prevalent musculoskeletal conditions, with hip and knee OA affecting over 300 million individuals worldwide.^1^ Furthermore, the prevalence of OA is increasing due to the ageing population and rising obesity levels.^2^ Individuals with OA often experience severe pain, impaired function and reduced quality of life.^3^ OA can have a profound psychosocial impact^4^ and results in substantial economic burden.^5^

National and international guidelines emphasise that patient education, self-management strategies and exercise are core elements for managing OA.^6^ However, implementation of OA guidelines is currently poor.^7^ Patients report receiving insufficient information about OA management^8^ and having a limited understanding of the condition.^4 9^ This negatively impacts patients’ health behaviours.^4 9^ A need for tailored interventions to support OA self-management has therefore been highlighted.^10^

Peer support interventions are an established approach for supporting chronic condition self-management.^11 12^ Various peer support models have been described, such as peer-led group programmes and peer mentorship.^13^ The latter involves a trained individual with a particular health condition (the ‘peer mentor’) providing one-to-one support to another individual with the same condition.^13^ This approach is likely to be particularly valuable for individuals with OA due to the heterogeneous nature of the condition, which means tailored support is crucial.^10 14^

Previous studies have highlighted the value of OA interventions incorporating peer support.^15^ However, no previous studies have explored an OA peer mentorship intervention. Although such an intervention offers multiple potential benefits, peer mentorship interventions can present feasibility/acceptability issues, such as high peer mentor attrition.^16^ Therefore, this feasibility trial aimed to develop and trial a peer mentorship intervention to improve OA self-management. Its key objectives were to determine the feasibility of conducting a randomised controlled trial (RCT) of the OA peer mentorship intervention in terms of: the feasibility/acceptability of the intervention and trial procedures; participant recruitment and retention; questionnaire completion rates; generating the sample size required for a definitive RCT; and estimating the intervention costs. Additionally, the potential impact of the intervention on patient-reported outcomes and resource use was explored.

## Methods

This was a 6-month parallel group randomised feasibility trial. A nested qualitative study was included (reported elsewhere). The trial was prospectively registered and conducted between 1 September 2017 and 16 February 2020. The trial is reported according to the CONSORT 2010 extension for randomised pilot and feasibility trials^17^ (online supplemental tables 1 and 2). The development and feasibility testing of the OA peer mentorship intervention was guided by the Medical Research Council guidance on developing and evaluating complex interventions.^18^

10.1136/bmjopen-2020-045389.supp1 Supplementary data

### Trial procedures and participants

#### Recruitment and consent

Potential participants were identified from rheumatology and orthopaedic clinics of one secondary care National Health Service (NHS) Trust and physiotherapy clinics and electronic records of one primary care NHS Trust. Both are large Trusts in Northern England. The initial approach was made via clinical staff during clinic appointments or through an invitation letter. Individuals interested in participating were provided with further details about the trial and screened for eligibility by a researcher at their clinic appointment or via telephone.

All participants provided written informed consent at their clinic appointment or during a baseline visit from a researcher. The latter took place in the participant’s home or another private location of the participant’s choice.

### Eligibility criteria

Inclusion criteria were: (1) aged ≥55 years old and (2) clinician-confirmed diagnosis of hip and/or knee OA.^19^ Exclusion criteria were: (1) presence of inflammatory arthritis (including gout and rheumatoid arthritis), (2) serious health conditions that would prevent participation and (3) listed for hip/knee replacement.

### Sample size

Recommendations for pilot studies suggest 20 participants per arm is acceptable assuming at worst a small effect size (Cohen’s d=0.2) for a continuous outcome and 80% power.^20^ Allowing for 20% attrition,^21^ the sample size was set at 25 participants per arm.

### Randomisation

Participants were randomised to the intervention or control group with 1:1 allocation using the Sealed Envelope online randomisation service.^22^ This was set up by an independent statistician to generate blocked randomisation with varying block lengths, stratified according to educational level. The researcher performing the randomisation was unable to access the allocation codes. Given the nature of the intervention, participant blinding was not possible.

### Intervention group

Intervention group participants received usual care and the OA peer mentorship intervention. Usual care consisted of information resources (an OA booklet from Arthritis Research UK (now Versus Arthritis)^23^ and a handout about local services/support groups/activities). The intervention group participants received the information resources during their initial mentorship session and were offered the opportunity to discuss the resources with their peer mentor.

### Control group

Control group participants received usual care only. The control group participants received the information resources during their baseline researcher visit and were offered the opportunity to discuss the resources with the researcher.

### OA peer mentorship intervention

#### Intervention development

The OA peer mentorship intervention was developed in two stages. The first stage included a rapid review of published primary studies investigating one-to-one peer support interventions. The review aimed to identify: the range of methods and approaches used in delivering peer support interventions; training and support approaches for peer mentors involved in intervention delivery; and challenges of developing and implementing face-to-face peer support to improve self-management of long-term conditions. Using Medline, CINAHL and PsycInfo, a search of the literature between 2007 and 2018 was undertaken using the terms and synonyms ‘peer support’, ‘long term condition’ and ‘intervention’. Thirteen papers were included.^16 24–35^ The findings highlighted the importance of encouraging a person-centred approach and retaining flexibility within the peer mentorship sessions. There was little information about ‘matching’ the mentee with the peer mentor, although some studies based this on factors such as gender and age. There were issues around recruitment of peer mentors and a need to provide support and guidance to peer mentors throughout the intervention.

A preliminary version of the OA peer mentorship intervention was developed based on the rapid review findings and the following sources:

- Guidelines on self-management and OA from organisations such as the UK National Institute for Health and Care Excellence and the UK charity Versus Arthritis.
- The ‘Staying Connected Programme’: an arthritis self-management programme previously run by Arthritis Care Northern Ireland (now Versus Arthritis Northern Ireland).^36^
- Input from project team members, including a consultant rheumatologist, a health psychologist and a physiotherapist specialising in activity pacing/chronic pain management.

The above-mentioned sources were also used to develop a draft peer mentor educational resource pack. The pack was designed to supplement peer mentor training sessions and be used a resource during mentorship sessions. The pack included a range of handouts that peer mentors could give to participants.

The second stage of the development process consisted of expert review sessions conducted with the following key stakeholders: study patient and public involvement (PPI) members (n=2); other older individuals with OA (n=5); health professionals (n=4); voluntary/community organisation representatives (n=4); and researchers (n=2). Review sessions were conducted face to face (group, paired or individual meetings), via telephone or via email. The stakeholders were provided with information about the provisional OA peer mentorship intervention and a copy of the draft educational pack. Stakeholders’ opinions of these were assessed using a pro forma.

Key refinements made based on the expert review sessions included:

- Peer mentors were encouraged not to cover too many topics in the first mentorship session to allow more time for developing rapport and managing participant expectations.
- Participants were provided with notebooks for recording goals, reflections and additional notes.
- The language used in the educational pack was simplified.
- Additional infographic handouts and further information on falls, local authority services and emotional well-being were added to the educational resource pack.

### Finalised intervention

**Figure 1:**
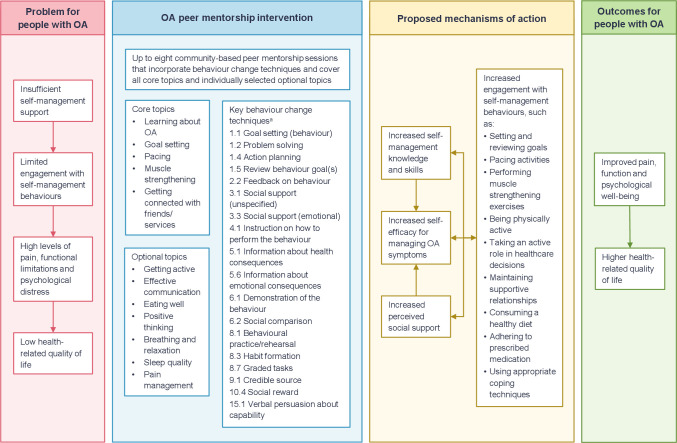
OA peer mentorship intervention logic model. ^a^Behaviour change techniques are coded using the Behaviour Change Technique Taxonomy version 1.^60^ OA, osteoarthritis.

The finalised OA peer mentorship intervention aimed to improve participants’ health outcomes through increasing their engagement with self-management behaviours. Figure 1 presents a logic model of the intervention, including the proposed mechanisms of action. The intervention involved up to eight 1-hour self-management support sessions delivered by a trained peer mentor. During the sessions, the peer mentor provided guided support that incorporated multiple behaviour change techniques (BCTs) and covered a combination of core and optional topics (figure 1). In line with a person-centred approach, the implementation of BCTs and the choice and order of topics covered was flexible and participant led. However, peer mentors were encouraged to cover all core topics at least once and set/review goals with the participant weekly. Online supplemental table 3 provides examples of the implementation of the key BCTs employed.

The peer mentorship sessions took place approximately weekly in the participant’s home or another private location of their choice. A person-centred approach was emphasised throughout.^37 38^ Therefore, the number of sessions and scheduling were flexible.

The volunteer coordinator attended the start of each initial session to introduce the participant and peer mentor, remind them of the trial and intervention aims and answer questions. The remainder of the initial session and all subsequent sessions were undertaken by the mentor alone. The volunteer coordinator contacted the participant and peer mentor following the initial session to check they were happy to continue with subsequent sessions with their mentorship match. Peer mentors completed a ‘session summary’ in writing following each session, detailing the topics covered during that session, any challenges encountered and their reflections on the progress made.

### Peer mentor eligibility and recruitment

Peer mentors were trained volunteers aged ≥50 years old with hip and/or knee OA who were able to travel independently. Multiple approaches were used to recruit peer mentors, including printed/social media advertisements and sharing trial information at local support/activity groups.

Potential peer mentors were asked to complete an application form, supply two references and take part in a telephone interview with the volunteer coordinator. Those assessed as suitable were invited to attend a compulsory 2-day training event. Prior to being matched with a participant, all peer mentors were required to complete enhanced Disclosure and Barring Service (criminal record) checks^39^ for safeguarding purposes.

### Peer mentor training and matching

Three training events were held due to varying peer mentor availability and staggering of the mentor recruitment, which meant it was not possible to train all mentors in one event. The first training event was provided by two external facilitators from Arthritis Care Northern Ireland and a trial team member (physiotherapist specialising in activity pacing/chronic pain management). The remaining events were provided by three trial team members (volunteer coordinator, the physiotherapist who facilitated the first training event and another musculoskeletal physiotherapist). Feedback from the first event was used to refine the content/delivery of the subsequent events.

The training events involved presentations and interactive activities covering OA self-management topics, mentorship skills and the practicalities of the peer mentor role (online supplemental table 4).

Participant/mentor matching was undertaken by the volunteer coordinator and the researcher who completed the baseline researcher visit. The initial intention was to base matching on location, gender preference, age and OA site(s). However, due to peer mentors’ preferences and discrepancies in the availability of intervention group participants and trained peer mentors, these criteria were relaxed.

### Data collection and analysis

#### Feasibility outcomes

This trial’s primary focus was on assessing the feasibility outcomes specified in table 1. Four associated success criteria for proceeding to a definitive RCT were prespecified based on relevant guidance^40^ (table 1).

**Table 1:** Feasibility outcomes and success criteria for proceeding to a definitive randomised controlled trial

Feasibility outcome	Details of feasibility outcome	Success criterion
Peer mentor recruitment rate	Number of peer mentors trained divided by the number individuals who were eligible for the peer mentor role	1. Demonstration that the intervention can be delivered in practice and is acceptable to participants and peer mentors*
Peer mentor attrition rate	Number of peer mentors who were not matched with a participant divided by the number of peer mentors trained	
Intervention completion rate	Number of participants who completed at least three peer mentorship sessions divided by the number of participants who commenced the intervention	
Participant recruitment rate	Number of participants randomised divided by the number of individuals who were eligible to participate	2. ≥60% of eligible individuals recruited
Attrition rate overall and in each group	Number of participants who did not complete the 6-month questionnaire divided by the number of participants randomised	3. <20% participant attrition with no evidence of attrition bias
Sample size required for a definitive RCT	Sample size calculation using the feasibility trial PIH scale scores assuming: 5% significance level; minimum power of 0.8; and 1:1 allocation ratio. The PIH scale scores were used because the PIH scale was chosen as the provisional primary outcome measure.	4. Calculation of a sample size that is achievable in a main trial
Questionnaire completion rate	Number of questionnaires completed divided by the number of participants provided with the questionnaires	None specified for these feasibility outcomes
Intervention fidelity	Content analysis of the mentors’ session summaries	
Cost of delivering the mentor training event	Estimation based on the events delivered by the trial team members accounting for: staff salary, national insurance and pension contributions; resources; refreshments; and peer mentor travel expenses; but not venue hire, estates and indirect costs	
Cost of delivering the intervention	Estimation based on the volunteer coordinator and peer mentor costs, excluding the training costs	

*No minimum/maximum rates were prespecified as acceptable for this success criterion.

PIH scale, revised 12-item Partners In Health scale; RCT, randomised controlled trial.

### Patient-reported outcomes

Patient-reported outcomes were assessed using paper self-report questionnaires administered at baseline, 8 weeks and 6 months. The baseline questionnaires were administered by a researcher or the volunteer coordinator. The majority of baseline questionnaires were administered after the participant had been informed of their group allocation. This approach was chosen due to the fluctuating availability of trained peer mentors. This meant there was sometimes a delay between participants being allocated to the intervention group and being able to commence their mentorship sessions. Therefore, most intervention group participants completed the baseline questionnaire with the volunteer coordinator immediately prior to their initial mentorship session to avoid a delay between completion of the baseline questionnaire and commencement of their mentorship sessions.

The 8-week and 6-month questionnaires were administered and returned via post. Non-responders were followed up with a second questionnaire posting 2 weeks later and a telephone call 4 weeks later.

The questionnaires covered participants’ sociodemographic and clinical characteristics and included validated tools assessing the biomedical/psychosocial outcomes specified below.

### Provisional primary outcome measure

- Revised 12-item Partners in Health (PIH) Scale: 12-item scale that assesses chronic condition self-management.^41^ Each item is scored on a 9-point Likert scale (0–8). Scores are summed to give a total score (0–96). Higher scores indicate higher self-management knowledge/behaviours.

### Provisional secondary outcome measures

- Multidimensional Scale of Perceived Social Support (MSPSS): 12-item scale that assesses perceived social support from a significant other, family and friends.^42^ Each item is scored on a 7-point Likert scale (1–7), and the mean for all items is calculated. Higher scores indicate greater perceived social support.
- Western Ontario and McMaster’s University Osteoarthritis Index Likert version (WOMAC 3.1): 24-item disease-specific scale with three subscales that assess pain (5 items), stiffness (2 items) and physical function (17 items).^43^ Each item is scored on a 5-point Likert scale (0–4). Scores are summed to give a total score (0–96) and subscale scores (pain: 1–20; stiffness: 0–8; physical function: 0–68). Higher scores indicate more severe problems.
- Eight-item Arthritis Self-Efficacy Scale English version: 8-item scale that assesses self-efficacy for managing arthritis symptoms.^44–46^ Each item is scored on a 10-point Likert scale (1–10), and the mean for all items is calculated. Higher scores indicate greater self-efficacy.
- Hospital Anxiety and Depression Scale (HADS): 14-item scale with two subscales that assess symptoms of anxiety (7 items) and depression (7 items).^47^ Each item is scored on a 4-point Likert scale (0–3). Scores are summed to give a total score (0–42) and subscale scores (0–21). Higher scores indicate more severe symptoms.
- EQ-5D-5L: Descriptive system and visual analogue scale (VAS) that assess general health status.^48 49^ The descriptive system includes five dimensions, each of which has five response levels. Each response is converted to a single-digit number. The numbers for each of the five dimensions can then be converted to a single index value anchored at 0 (a state equivalent to dead) and 1 (full health). The VAS consists of a single score on a scale from 0 (worst health imaginable) to 100 (best health imaginable).

The 8-week and 6-month questionnaires included sections on healthcare and community resource use, adapted from the Client Services Receipt Inventory.^50^

Missing data were addressed in line with guidance for the relevant outcome measurement tool. The quantitative questionnaire responses were analysed using STATA (V.15). Given the trial was not powered to detect statistically significant differences, the analyses were predominantly descriptive. However, exploratory analyses of the outcome measure data and healthcare resource use data were performed. The outcome scores were tested for normality at the 5% significance level.^51^ Between-group comparisons were made using analysis of covariance models and, where the scores were significantly skewed, quantile regression models were used.^52^

### Patient and public involvement

PPI played a key role in this trial as follows:

- During the trial development stage, six individuals with OA were consulted for research ideas, and eight members of a local musculoskeletal PPI group participated in a group discussion. These patient representatives emphasised that one-to-one mentorship would be preferable to a group-based approach and felt the questionnaire burden was appropriate.
- Three patient representatives became ‘study PPI members’ and assisted with the conduct of the study, reviewing lay materials and finalising the dissemination plans. These PPI members were also invited to attend peer mentorship training sessions.
- Two study PPI members and five other individuals with OA helped refine the provisional intervention and educational resources through expert review sessions.
- The peer mentors were all trained volunteers with hip and/or knee OA. Peer mentors were encouraged to give regular informal feedback about their involvement in the trial, as well as being invited to participate in a nested qualitative study (reported elsewhere).

## Results

### Feasibility outcomes

### Participant flow and intervention completion

**Figure 2:**
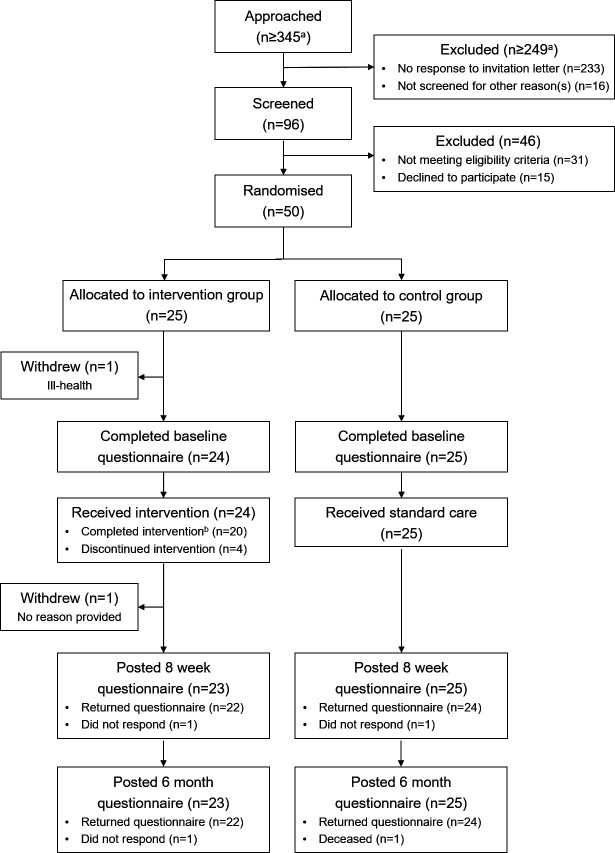
Participant flow diagram. ^a^Minimum number of individuals approached/excluded. Additional individuals may have been approached/excluded because the number of individuals approached through physiotherapy clinics was not recorded to minimise the administrative burden on physiotherapists. ^b^Intervention completion was defined as completion of at least three peer mentorship sessions.

Participant recruitment took place between 22 November 2018 and 30 May 2019. The final questionnaire was completed on the 16 February 2020. At least 345 individuals were approached (figure 2). Ninety-six individuals were screened, of whom 65 were eligible. The most common reason for ineligibility was being listed for joint replacement (n=8). Fifty individuals were randomised. Therefore, the participant recruitment rate was 77%. The most common reason for declining participation was insufficient time/other commitments (n=7). Most participants were recruited through the primary care physiotherapy department (n=44).

One intervention group participant withdrew due to ill-health prior to completing the baseline questionnaire and commencing the intervention. Of the 24 participants who commenced the intervention, 20 completed at least three mentorship sessions (figure 2). The intervention completion rate was therefore 83%. Reasons for discontinuing the intervention largely related to participants feeling the intervention was not relevant to their needs, for example, due to having mild OA symptoms and/or feeling they did not require additional self-management support. The mean number of mentorship sessions received was 5.79 (SD=2.25; median=7). No harms/unintended effects related to the intervention occurred.

One intervention participant who discontinued the intervention after two mentorship sessions withdrew (no reason provided). One additional intervention participant who discontinued the intervention after two mentorship sessions did not complete the follow-up questionnaires. One control group participant did not complete the follow-up questionnaires. A family member of this control group participant returned his 6-month questionnaire indicating he had died. The attrition rates were therefore: 8% overall, 12% in the intervention group and 4% in the control group.

### Questionnaire completion

Among the participants who were provided with the questionnaires, the questionnaire completion rate for both groups was 100% at baseline and 96% at 8 weeks and 6 months (figure 2). Minor issues were noted with the resource use sections. Some participants duplicated details, for example, by recording the same physiotherapy appointments under ‘Hospital services’ and ‘Services outside the hospital’. Additionally, the ‘day activities’ reported by participants largely related to their hobbies, such as yoga and dancing, rather than social/community support activities.

### Peer mentor flow and characteristics

Peer mentor recruitment took place between May 2018 and January 2019. Thirty-two individuals enquired about the role, of whom 21 were eligible (online supplemental figure 1). Five eligible individuals were unable to attend the training events, one withdrew due to ill-health and 15 were trained. The peer mentor recruitment rate was therefore 71%. Five trained mentors withdrew prior to being matched, most commonly due to ill-health (n=3). The peer mentor attrition rate was therefore 33%. The most successful peer mentor recruitment approach was a local community magazine advertisement, which led to recruitment of five active peer mentors.

Nine active peer mentors were female and one was male. Their mean age was 68 years old (SD=5.5; range=57–75). All had been living with hip and/or knee OA for at least 3 years. Nine were retired, although three had only recently stopped work, and one worked full-time. Each active peer mentor supported between one and four participants (online supplemental table 1).

### Session summary data

The session summaries indicated the following topics were covered by almost all participant/mentor dyads and were the most frequently covered overall: learning about OA; goal setting; pacing; muscle strengthening; and pain management. In addition, getting active and eating well were frequently covered.

The least frequently covered topics were: getting connected with friends/services; and effective communication. Correspondingly, these topics were covered by only six and eight dyads, respectively. Positive thinking and sleep quality were also rarely covered.

### Intervention costs

The estimated cost of delivering the training event to five peer mentors (mean number of mentors per event) was £239 per mentor (online supplemental table 5). The estimated cost of delivering the intervention based on the mean number of 5.79 mentorship sessions was £274 per participant (online supplemental table 6).

### Sample size calculation

Based on the assumptions stated in table 1, between-groups variance and within-groups variance of the PIH scale at 6 months of 369.1 and 6237.1, respectively, and detecting a difference in the PIH scale of 4.4 (effect size at 6 months), the sample size required for a definitive RCT was calculated as 136 participants (68 per group). Retaining the same attrition rate as for the feasibility trial (20%) would give a required sample size of 170 (85 per group).

### Patient-reported outcomes

#### Participant baseline characteristics

Table 2 provides a summary of participants’ baseline sociodemographic and clinical characteristics. Additional details are available in online supplemental table 7.

**Table 2:** Participant baseline characteristics

Characteristics	Intervention n=24	Control n=25
Age in years, mean (SD)	70.0 (8.6)	69.3 (8.1)
Men, n (%)	10 (41.7)	4 (16.0)
Ethnicity, white, n (%)	21 (87.5)	24 (96.0)
Further education*, n (%)	15 (62.5)	15 (60.0)
Employed, n (%)	7 (29.2)	5 (20.0)
Retired, n (%)	17 (70.8)	20 (80.0)
Body mass index in kg/m^2^, mean (SD)	27.5 (6.3)	28.5 (5.5)
Duration of arthritis diagnosis in years, mean (SD)	4.7 (5.2)	5.3 (6.2)
At least one hip affected by arthritis, n (%)	14 (58.3)	13 (52.0)
At least one knee affected by arthritis, n (%)	22 (91.7)	21 (84.0)
VAS for current pain due hip/knee arthritis, mean (SD)	52.8 (26.8)	48.6 (26.3)
VAS for ability to cope with/manage arthritis in general, mean (SD)	66.2 (21.8)	64.4 (21.8)
Number of joints affected by pain for >6 weeks in the last 3 months, median (IRQ)	5 (4)	3 (3)
Number of comorbidities†, n (%)		
0	8 (33.3)	6 (24.0)
1	5 (20.8)	10 (40.0)
2	5 (20.8)	3 (12.0)
≥3	6 (25.0)	6 (24.0)

*Further education was defined as any formal education undertaken once the participant was aged over 16 years old.

†Comorbidities include: diabetes; asthma; bronchitis; gastrointestinal problems; angina/heart problems; high blood pressure; depression; and anxiety.

VAS, Visual Analogue Scale (0–100 mm).

### Provisional primary outcome

The mean PIH scores at baseline were 73.9 and 76.5 for the intervention and control groups, respectively (table 3). After 8 weeks, the mean PIH score improved in the intervention group (mean change=2.6; SD=13.2) and deteriorated in the control group (mean change= −7; SD=12.3). Thus, the intervention group participants appeared to have better self-management knowledge/behaviours than the control group participants at 8 weeks (effect size=8.3; 95% CI 2.2 to 14.4) (table 4). At 6 months, the intervention group’s mean PIH score was similar to the group’s score at 8 weeks, while the control group’s mean PIH score increased but remained lower than at baseline. Overall, the intervention group had higher PIH scores than the control group after 6 months (effect size=4.4; 95% CI −2.8 to 11.6) (table 4).

**Table 3:** Outcome measurement scores at baseline, 8 weeks and 6 months

Outcome measure	Baseline means (SD)		8-week means (SD)		6-month means (SD)	
	Intervention (n=22)	Control (n=24)	Intervention (n=22)	Control (n=24)	Intervention (n=22)	Control (n=24)
PIH scale	73.9 (16.3)	76.5 (14.3)	76.5 (14.5)	69.5 (10.6)	76.0 (13.4)	73.0 (15.0)
MSPSS	5.3 (1.1)	5.7 (0.98)	4.9 (1.6)	5.2 (1.6)	4.9 (1.3)	5.4 (1.4)
WOMAC total*	32.6 (18.8)	41.9 (19.5)	34.0 (17.8)	43.5 (19.4)	34.7 (18.5)	41.3 (17.8)
WOMAC pain*	6.8 (4.5)	9.0 (4.2)	7.5 (3.9)	9.6 (4.1)	7.7 (4.0)	8.9 (3.2)
WOMAC stiffness*	3.7 (1.6)	4.3 (1.9)	3.3 (1.3)	4.3 (1.6)	3.4 (1.3)	4.0 (1.7)
WOMAC function*	22.1 (14.0)	28.5 (14.6)	23.2 (13.2)	29.6 (15.3)	23.6 (14.8)	28.3 (13.5)
ASES-8	6.0 (1.9)	5.9 (1.7)	5.5 (2.2)	6.5 (2.4)	6.3 (1.6)	5.3 (2.5)
HADS total*	9.4 (4.4)	11.3 (7.2)	10.5 (5.7)	12.7 (8.3)	10.7 (6.2)	12.8 (8.4)
HADS anxiety*	5.3 (3.1)	6.7 (4.3)	6.3 (3.4)	7.3 (4.8)	5.9 (3.9)	7.5 (4.9)
HADS depression*	4.1 (2.0)	4.6 (3.6)	4.3 (3.1)	5.4 (4.0)	4.8 (2.7)	5.3 (4.0)
EQ-5D-5L index‡, †	0.723 (0.139)	0.645 (0.170)	0.691 (0.099)	0.647 (0.249)	0.687 (0.088)	0.647 (0.183)
EQ-5D-5L VAS†	70 (15.0)	75 (20.0)	70 (25.0)	70 (22.5)	75 (20.0)	70 (23.5)

*Higher scores indicate more severe problems.

†Median (IQR).

‡EQ-5D-5L index value control group n=23 due to missing data.

ASES-8, 8-item Arthritis Self-Efficacy Scale English version; HADS, Hospital Anxiety and Depression Scale; MSPSS, Multidimensional Scale of Perceived Social Support; PIH scale, revised 12-item Partners in Health Scale; VAS, Visual Analogue Scale; WOMAC, Likert version of the Western Ontario and McMaster’s University Osteoarthritis Index.

**Table 4:** Outcome measurements score changes and effect of the intervention at 8 weeks and 6 months

Outcome measure	Changes at 8 weeks		Effect size at 8 weeks, mean (95% CI)*	Changes at 6 months		Effect size at 6 months, mean (95% CI)*
	Intervention	Control		Intervention	Control	
PIH scale	2.6 (13.2)	−7.0 (12.3)	8.3 (2.2 to 14.4)	2.1 (10.6)	−3.5 (16.6)	4.4 (−2.8 to 11.6)
MSPSS	−0.28 (1.4)†	−0.25 (1.2)†	0.0 (−0.80 to 0.80)‡	−0.42 (0.93)†	−0.06 (0.6)†	−0.40 (−0.84 to 0.03)‡
WOMAC total§	3.0 (19)†	5.0 (22)†	−2.6 (−10.2 to 4.9)‡	2.1 (8.6)	−0.59 (19.1)	−0.65 (−8.9 to 7.6)
WOMAC pain§	0.64 (2.6)	0.63 (3.7)	−0.75 (−2.5 to 1.0)	0.91 (2.4)	−0.09 (3.8)	0.06 (−1.6 to 1.7)
WOMAC stiffness§	−0.36 (1.5)	−0.04 (1.1)	−0.59 (−1.3 to 0.08)	−0.27 (1.8)	−0.29 (1.2)	−0.31 (−1.1 to 0.46)
WOMAC function§	1.1 (7.8)	1.0 (10.4)	−1.3 (−6.7 to 4.1)	1.5 (7.8)	−0.21 (15.4)	−0.8 (−7.7 to 6.0)
ASES-8	0.48 (1.6)	−0.04 (2.25)	0.64 (−0.51 to 1.8)	0.69 (3.0)†	0.0 (3.3)†	0.34 (−1.3 to 1.9)‡
HADS total§	1.12 (3.8)	1.38 (4.5)	−0.27 (−2.8 to 2.3)	1.33 (3.4)	1.5 (4.3)	−0.01 (−2.4 to 2.4)
HADS anxiety§	0.95 (2.8)	0.58 (2.1)	0.26 (−1.2 to 1.7)	0.59 (2.4)	0.79 (2.6)	−0.23 (−1.8 to 1.3)
HADS depression§	0.20 (2.4)	0.79 (3.4)	−0.74 (−2.5 to 1.0)	0.74 (1.6)	0.67 (2.5)	0.03 (−1.3 to 1.3)
EQ-5D-5L index¶	−0.057 (0.20)	0 (0.12)	−0.06 (−0.18 to 0.06)	−0.053 (0.14)	0 (0.19)	−0.04 (−0.12 to 0.04)
EQ-5D-5L VAS	−2.5 (20)†	0 (10)†	0 (−10.8 to 10.8)‡	0 (20)†	−5 (21)†	6.7 (−4.6 to 18.0)‡

*Analysis of covariance models adjusted for baseline outcomes.

†Median (IQR).

‡Median (95% CIs).

§Higher scores indicate more severe problems.

¶EQ-5D-5L index value control group n=23 due to missing data.

ASES-8, 8-item Arthritis Self-Efficacy Scale English version; HADS, Hospital Anxiety and Depression Scale; MSPSS, Multidimensional Scale of Perceived Social Support; PIH scale, revised 12-item Partners in Health Scale; WOMAC, Likert version of the Western Ontario and McMaster’s University Osteoarthritis Index.

### Provisional secondary outcomes

There were no significant between-group differences for any of the secondary outcomes at 8 weeks or 6 months. However, the mean WOMAC and HADS scores were lower in the intervention group than the control group at baseline and both follow-up time-points.

### Resource use

Healthcare resource use between baseline and 6 months was largely similar between groups. The main differences were more overnight hospital stays in the control group (mean difference between groups=−0.91; 95% CI −1.78 to −0.04), and more GP practice nurse visits in the intervention group (mean difference between groups=0.93; 95% CI 0.15 to 1.71) (online supplemental table 8). No participants reported attending a day centre. Two intervention group participants and four control group participants reported receiving help with daily activities.

## Discussion

This feasibility trial aimed to develop and trial a peer mentorship intervention to improve OA self-management. All four prespecified success criteria for proceeding to a definitive RCT (table 1) were achieved. The peer mentor recruitment rate (71%), peer mentor attrition rate (33%) and intervention completion rate (83%) all compare favourably with previous peer mentorship studies.^16 32 53 54^ This suggests the OA peer mentorship intervention can be delivered in practice and is acceptable to participants and peer mentors. However, some participants discontinued the intervention due to having mild OA symptoms and feeling they did not need self-management support. This could be overcome by specifying a minimum symptom severity threshold and/or a maximum arthritis-related self-efficacy threshold in the eligibility criteria to prevent inclusion of individuals who have mild symptoms and/or already feel confident about managing their symptoms.

The participant recruitment rate was 77% and hence well above the prespecified minimum threshold of 60%. However, this recruitment rate did not account for individuals lost prior to screening. The overall participant attrition rate at 6 months was 8% and hence well below the prespecified maximum threshold of 20%. This suggests that additional strategies to improve participant recruitment and retention would not be needed for a future RCT. Allowing for 20% attrition, the sample size required for a definitive RCT was calculated as 170 participants (85 per group). Assuming each peer mentor supports two participants, this would require 43 mentors. This target RCT sample size is achievable. However, only 10 active peer mentors were recruited during this trial. Additional strategies would therefore be needed to recruit sufficient mentors. These could include using multiple recruitment sites across a wider geographical area, recruiting mentors over a longer time-period, using snowballing recruitment with previously trained peer mentors and optimising recruitment through media outlets, such as magazine advertisements.

The findings for the remaining feasibility outcomes were also encouraging. The questionnaire completion rates were consistently high (96%–100%). However, minor modifications to the resource use sections would be beneficial to avoid participants duplicating details/reporting unnecessary details. The session summary data indicated intervention fidelity was good overall, with most participant/mentor dyads covering four of the five core topics. The remaining core topic, getting connected with friends/services, was only covered by six dyads. This may be because the participants already had good social support networks, as suggested by their relatively high MSPSS scores. Additionally, most dyads covered the optional topic of pain management. Therefore, changing getting connected to an optional topic and pain management to a core topic warrants consideration.

The estimated costs of training peer mentors and delivering the intervention were £239 per mentor and £274 per participant respectively. The training costs would have been lower if fewer training events had been run with more mentors per event. This was not possible due to varying peer mentor availability and staggering of the mentor recruitment. A similar issue was noted in a study of peer mentorship for people with advanced cancer^54^ and would need accounting for in a future RCT.

This trial included exploratory analyses of the impact of the OA peer mentorship intervention on patient-reported outcomes. These analyses provide a preliminary insight into the potential effectiveness of the intervention. However, the results must be interpreted cautiously because the present trial was not powered to detect statistically significant differences. The exploratory analyses suggest the OA peer mentorship intervention may improve self-management knowledge/behaviours (table 4). However, no significant effects of the OA peer mentorship intervention on other outcomes, such as pain and arthritis-related self-efficacy, were observed in the present trial.

A Cochrane review identified low to moderate quality evidence indicating self-management education programmes for people with OA do not result in any clinically meaningful benefits.^55^ Despite this, the authors concluded that trials investigating alternative self-management support approaches, particularly those involving tailored support, are warranted. One such approach is the Staying Connected Programme, a tailored one-to-one 8-week self-management programme delivered by trained volunteers.^36^ A recent quasi-experimental study identified significant improvements in pain and arthritis-related self-efficacy among individuals with arthritis who participated in this programme.^36^ The Staying Connected Programme volunteers were not required to have an arthritis diagnosis, despite peer support being recognised as a valuable approach for supporting self-management.^11 12^ In addition, the Staying Connected Programme aimed to support individuals with various types of arthritis and other long-term conditions.^36^ The present trial’s intervention therefore aimed to replicate some key elements of the Staying Connected Programme while also incorporating peer support and being tailored specifically to the needs of people with OA. Although there are disparities between the findings of the present trial and the Staying Connected Programme study, neither was an adequately powered RCT. Future work is therefore required to determine the effectiveness of both the present trial’s OA peer mentorship intervention and the Staying Connected Programme.

In addition to drawing on the Staying Connected Programme, the development of the OA peer mentorship intervention incorporated multiple other sources and an expert review with key stakeholders. This approach, combined with extensive PPI, helped ensure the intervention is feasible, acceptable and focused on the needs of individuals with OA. The substantial investment in the development process will also maximise the chances of the intervention proving effective during a future definitive RCT. A potential limitation is that the development process was not based on a single behaviour change theory or theoretical framework. However, the broad range of sources considered and input from multidisciplinary experts helped ensure that the intervention has a sound theoretical basis (figure 1). In particular, the focus on enhancing self-efficacy is consistent with other peer support interventions aimed at improving chronic condition self-management.^24 32 56 57^

This trial also presents additional limitations. Notably, 88% of participants were recruited through a physiotherapy service and hence had already received some self-management support. This could be addressed through using alternative recruitment sites, such as GP practices. Furthermore, the majority of participants were older, white, well-educated females, and a Cochrane review identified that the impact of self-management education programmes for OA may vary between Caucasian, educated females and other subgroups.^55^ Additionally, peer support interventions may be most effective among the ‘hardly reached’, such as individuals with lower education levels.^58^ Targeting the OA peer mentorship intervention to specific subgroups could therefore be valuable. Participants and peer mentors’ experiences of the OA peer mentorship intervention were explored through a nested qualitative study (reported elsewhere). However, the impact of providing the intervention on the peer mentors was not quantitatively assessed. This is an important consideration because providing peer mentorship may have positive and/or negative effects on peer mentors.^11 26^ Another limitation of the present trial was that most participants were aware of their group allocation when completing the baseline questionnaire, which may have influenced their questionnaire responses.^59^ This could be overcome in a future definitive RCT by ensuring a greater number of trained peer mentors are available and/or administering the baseline questionnaire by post/online.

In conclusion, this trial’s findings suggest peer mentorship is a feasible, acceptable and promising approach for improving OA self-management. Further investigation of the OA peer mentorship intervention is therefore warranted. However, minor modifications to the intervention and trial procedures should be considered, particularly regarding the participant and peer mentor recruitment procedures.

## Supplementary Material

Reviewer comments

Author's manuscript

## Data Availability

Data are available on reasonable request. Deidentified participant data and the trial protocol are available from the corresponding author via A.Anderson@leeds.ac.uk on reasonable request. Reuse is permitted for the purpose of health and care research as long as the original creators are acknowledged.

## References

[1] GBD 2017 Disease and Injury Incidence and Prevalence Collaborators. Global, regional, and national incidence, prevalence, and years lived with disability for 354 diseases and injuries for 195 countries and territories, 1990-2017: a systematic analysis for the global burden of disease study 2017. Lancet 2018;392:1789–858. 10.1016/S0140-6736(18)32279-7 30496104 PMC6227754

[2] Turkiewicz A, Petersson IF, Björk J, et al. Current and future impact of osteoarthritis on health care: a population-based study with projections to year 2032. Osteoarthritis Cartilage 2014;22:1826–32. 10.1016/j.joca.2014.07.015 25084132

[3] Hunter DJ, Schofield D, Callander E. The individual and socioeconomic impact of osteoarthritis. Nat Rev Rheumatol 2014;10:437–41. 10.1038/nrrheum.2014.44 24662640

[4] Smith TO, Purdy R, Lister S, et al. Living with osteoarthritis: a systematic review and meta-ethnography. Scand J Rheumatol 2014;43:441–52. 10.3109/03009742.2014.894569 24882107

[5] Puig-Junoy J, Ruiz Zamora A. Socio-Economic costs of osteoarthritis: a systematic review of cost-of-illness studies. Semin Arthritis Rheum 2015;44:531–41. 10.1016/j.semarthrit.2014.10.012 25511476

[6] Nelson AE, Allen KD, Golightly YM, et al. A systematic review of recommendations and guidelines for the management of osteoarthritis: the chronic osteoarthritis management initiative of the U.S. bone and joint initiative. Semin Arthritis Rheum 2014;43:701–12. 10.1016/j.semarthrit.2013.11.012 24387819

[7] Basedow M, Esterman A. Assessing appropriateness of osteoarthritis care using quality indicators: a systematic review. J Eval Clin Pract 2015;21:782–9. 10.1111/jep.12402 26083547

[8] McHugh GA, Campbell M, Luker KA. Quality of care for individuals with osteoarthritis: a longitudinal study. J Eval Clin Pract 2012;18:534–41. 10.1111/j.1365-2753.2010.01616.x 21210900

[9] Darlow B, Brown M, Thompson B, et al. Living with osteoarthritis is a balancing act: an exploration of patients' beliefs about knee pain. BMC Rheumatol 2018;2:15. 10.1186/s41927-018-0023-x 30886966 PMC6390552

[10] Hunter DJ, Bowden JL. Therapy: are you managing osteoarthritis appropriately? Nat Rev Rheumatol 2017;13:703–4. 10.1038/nrrheum.2017.160 28931953

[11] Embuldeniya G, Veinot P, Bell E, et al. The experience and impact of chronic disease peer support interventions: a qualitative synthesis. Patient Educ Couns 2013;92:3–12. 10.1016/j.pec.2013.02.002 23453850

[12] Fisher EB, Boothroyd RI, Elstad EA, et al. Peer support of complex health behaviors in prevention and disease management with special reference to diabetes: systematic reviews. Clin Diabetes Endocrinol 2017;3:4. 10.1186/s40842-017-0042-3 28702258 PMC5471959

[13] Heisler M. Overview of peer support models to improve diabetes self-management and clinical outcomes. Diabetes Spectrum 2007;20:214–21. 10.2337/diaspect.20.4.214

[14] Hunter DJ, Bierma-Zeinstra S. Osteoarthritis. Lancet 2019;393:1745–59. 10.1016/S0140-6736(19)30417-9 31034380

[15] Ali SA, Kokorelias KM, MacDermid JC, et al. Education and social support as key factors in osteoarthritis management programs: a scoping review. Arthritis 2018;2018:2496190. 10.1155/2018/2496190 29854457 PMC5964569

[16] Simmons D, Cohn S, Bunn C, et al. Testing a peer support intervention for people with type 2 diabetes: a pilot for a randomised controlled trial. BMC Fam Pract 2013;14:5. 10.1186/1471-2296-14-5 23297781 PMC3546024

[17] Eldridge SM, Chan CL, Campbell MJ, et al. Consort 2010 statement: extension to randomised pilot and feasibility trials. BMJ 2016;355:i5239. 10.1136/bmj.i5239 27777223 PMC5076380

[18] Craig P, Dieppe P, Macintyre S. Developing and evaluating complex interventions: new guidance. UK: Medical Research Council, 2006.

[19] National Institute for Health and Care Excellence. Osteoarthritis: care and management (Clinical guideline [CG177]). London: National Institute for Health and Care Excellence, 2014.

[20] Whitehead AL, Julious SA, Cooper CL, et al. Estimating the sample size for a pilot randomised trial to minimise the overall trial sample size for the external pilot and main trial for a continuous outcome variable. Stat Methods Med Res 2016;25:1057–73. 10.1177/0962280215588241 26092476 PMC4876429

[21] McHugh GA, Campbell M, Luker KA. Gp referral of patients with osteoarthritis for consideration of total joint replacement: a longitudinal study. Br J Gen Pract 2011;61:e459–68. 10.3399/bjgp11X588420 21801538 PMC3145529

[22] Sealed Envelope Ltd. Simple randomisation service 2019, 2020. Available: https://www.sealedenvelope.com/simple-randomiser/v1/

[23] Arthritis Research UK. Osteoarthritis. Chesterfield, UK: Arthritis Research UK, 2012.

[24] Jerant A, Moore-Hill M, Franks P. Home-Based, peer-led chronic illness self-management training: findings from a 1-year randomized controlled trial. Ann Fam Med 2009;7:319–27. 10.1370/afm.996 19597169 PMC2713168

[25] Chapin RK, Sergeant JF, Landry S, et al. Reclaiming joy: pilot evaluation of a mental health peer support program for older adults who receive Medicaid. Gerontologist 2013;53:345–52. 10.1093/geront/gns120 22936540

[26] Sandhu S, Veinot P, Embuldeniya G, et al. Peer-to-peer mentoring for individuals with early inflammatory arthritis: feasibility pilot. BMJ Open 2013;3. 10.1136/bmjopen-2012-002267. [Epub ahead of print: 01 Mar 2013]. PMC3612764 23457326

[27] Simmons D, Prevost AT, Bunn C, et al. Impact of community based peer support in type 2 diabetes: a cluster randomised controlled trial of individual and/or group approaches. PLoS One 2015;10:e0120277. 10.1371/journal.pone.0120277 25785452 PMC4364716

[28] Thom DH, Ghorob A, Hessler D, et al. Impact of peer health coaching on glycemic control in low-income patients with diabetes: a randomized controlled trial. Ann Fam Med 2013;11:137–44. 10.1370/afm.1443 23508600 PMC3601392

[29] Iliffe S, Kendrick D, Morris R, et al. Multicentre cluster randomised trial comparing a community group exercise programme and home-based exercise with usual care for people aged 65 years and over in primary care. Health Technol Assess 2014;18:1–106. 10.3310/hta18490 PMC4781144 25098959

[30] Ayala GX, Ibarra L, Cherrington AL, et al. Puentes hacia Una mejor vida (bridges to a better life): outcome of a diabetes control peer support intervention. Ann Fam Med 2015;13 Suppl 1:S9–17. 10.1370/afm.1807 26304977 PMC4648133

[31] Matthias MS, McGuire AB, Kukla M, et al. A brief peer support intervention for veterans with chronic musculoskeletal pain: a pilot study of feasibility and effectiveness. Pain Med 2015;16:81–7. 10.1111/pme.12571 25312858 PMC4793916

[32] Cooper K, Jehu LM, Klein S, et al. Training Peers to support older people with chronic low back pain following physiotherapy discharge: a feasibility study. Physiotherapy 2018;104:239–47. 10.1016/j.physio.2017.07.001 28789781

[33] Cooper K, Schofield P, Klein S, et al. Exploring peer-mentoring for community dwelling older adults with chronic low back pain: a qualitative study. Physiotherapy 2017;103:138–45. 10.1016/j.physio.2016.05.005 27613081 PMC5441170

[34] Stevens Z, Barlow C, Iliffe S. Promoting physical activity among older people in primary care using peer mentors. Prim Health Care Res Dev 2015;16:201–6. 10.1017/S1463423613000510 24451938

[35] Matthias MS, Kukla M, McGuire AB, et al. Facilitators and barriers to participation in a peer support intervention for veterans with chronic pain. Clin J Pain 2016;32:534–40. 10.1097/AJP.0000000000000297 26340653 PMC4794408

[36] McHugh GA, Conaghan PG, McConville M, et al. Promoting self-management in older people with arthritis: preliminary findings of the Northern Ireland staying connected programme. Musculoskeletal Care 2018;16:489–93. 10.1002/msc.1353 29984887

[37] Olsson L-E, Jakobsson Ung E, Swedberg K, et al. Efficacy of person-centred care as an intervention in controlled trials - a systematic review. J Clin Nurs 2013;22:456–65. 10.1111/jocn.12039 23231540

[38] nhs.uk. Involving people in their own care n.d, 2020. Available: https://www.england.nhs.uk/ourwork/patient-participation/

[39] Crown copyright. Disclosure & Barring Service: About us: GOV.UK; n.d., 2021. Available: https://www.gov.uk/government/organisations/disclosure-and-barring-service/about

[40] Thabane L, Ma J, Chu R, et al. A tutorial on pilot studies: the what, why and how. BMC Med Res Methodol 2010;10:1. 10.1186/1471-2288-10-1 20053272 PMC2824145

[41] Smith D, Harvey P, Lawn S, et al. Measuring chronic condition self-management in an Australian community: factor structure of the revised partners in health (PIH) scale. Qual Life Res 2017;26:149–59. 10.1007/s11136-016-1368-5 27432251

[42] Zimet GD, Dahlem NW, Zimet SG, et al. The multidimensional scale of perceived social support. J Pers Assess 1988;52:30–41. 10.1207/s15327752jpa5201_2

[43] Bellamy N, Buchanan WW, Goldsmith CH, et al. Validation study of WOMAC: a health status instrument for measuring clinically important patient relevant outcomes to antirheumatic drug therapy in patients with osteoarthritis of the hip or knee. J Rheumatol 1988;15:1833–40. 3068365

[44] Lorig K, Chastain RL, Ung E, et al. Development and evaluation of a scale to measure perceived self-efficacy in people with arthritis. Arthritis Rheum 1989;32:37–44. 10.1002/anr.1780320107 2912463

[45] Self-Management Resource Center. English evaluation tools: arthritis self-efficacy 2020, 2020. Available: https://www.selfmanagementresource.com/resources/evaluation-tools/english-evaluation-tools

[46] Brady TJ. Measures of self-efficacy: arthritis self-efficacy scale (ases), arthritis self-efficacy Scale-8 item (ASES-8), children's arthritis self-efficacy scale (case), chronic disease self-efficacy scale (CDSES), parent's arthritis self-efficacy scale (PASE), and rheumatoid arthritis self-efficacy scale (RASE). Arthritis Care Res 2011;63 Suppl 11:S473–85. 10.1002/acr.20567 22588769

[47] Zigmond AS, Snaith RP. The hospital anxiety and depression scale. Acta Psychiatr Scand 1983;67:361–70. 10.1111/j.1600-0447.1983.tb09716.x 6880820

[48] Herdman M, Gudex C, Lloyd A, et al. Development and preliminary testing of the new five-level version of EQ-5D (EQ-5D-5L). Qual Life Res 2011;20:1727–36. 10.1007/s11136-011-9903-x 21479777 PMC3220807

[49] van Hout B, Janssen MF, Feng Y-S, et al. Interim scoring for the EQ-5D-5L: mapping the EQ-5D-5L to EQ-5D-3L value sets. Value Health 2012;15:708–15. 10.1016/j.jval.2012.02.008 22867780

[50] Beecham J, Knapp M. Costing psychiatric interventions. In: Thornicroft G, ed. Measuring mental health needs. London. United Kingdom: Gaskell, 2001: 200–24.

[51] D'Agostino RB, Belanger A, D'Agostino RB. A suggestion for using powerful and informative tests of normality. Am Stat 1990;44:316–21.

[52] Koenker R, Hallock KF. Quantile regression. J Econ Perspect 2001;15:143–56.

[53] Williams EM, Hyer JM, Viswanathan R, et al. Peer-to-Peer mentoring for African American women with lupus: a feasibility pilot. Arthritis Care Res 2018;70:908–17. 10.1002/acr.23412 PMC5962359 29161471

[54] Walshe C, Roberts D, Calman L, et al. Peer mentors for people with advanced cancer: lessons learnt from recruiting and training peer mentors for a feasibility randomized controlled trial. J Cancer Educ 2020. 10.1007/s13187-020-01692-7. [Epub ahead of print: 28 Jan 2020]. PMC8328854 31994006

[55] Kroon FPB, van der Burg LRA, Buchbinder R. Self‐management education programmes for osteoarthritis. Cochrane Database of Syst Rev 2014;1. 10.1002/14651858.CD008963.pub2 PMC11104559 24425500

[56] Lorig KR, Ritter P, Stewart AL, et al. Chronic disease self-management program: 2-year health status and health care utilization outcomes. Med Care 2001;39:1217–23. 10.1097/00005650-200111000-00008 11606875

[57] Dale J, Caramlau I, Sturt J, et al. Telephone peer-delivered intervention for diabetes motivation and support: the telecare exploratory RCT. Patient Educ Couns 2009;75:91–8. 10.1016/j.pec.2008.09.014 19013741

[58] Sokol R, Fisher E. Peer support for the hardly reached: a systematic review. Am J Public Health 2016;106:e1–8. 10.2105/AJPH.2016.303180 PMC4984766 27196645

[59] Higgins JPT, Savović J, Page MJ. Revised Cochrane risk-of-bias tool for randomized trials (rob 2), 2019. Available: https://sites.google.com/site/riskofbiastool/welcome/rob-2-0-tool/current-version-of-rob-2

[60] Michie S, Richardson M, Johnston M, et al. The behavior change technique taxonomy (V1) of 93 hierarchically clustered techniques: building an international consensus for the reporting of behavior change interventions. Ann Behav Med 2013;46:81–95. 10.1007/s12160-013-9486-6 23512568

