# Supplementary material for: Peer mentorship to improve self-management of hip and knee osteoarthritis: a randomised feasibility trial
Source: BMJ Open. 2021 Jul 21;11(7):e045389. doi: 10.1136/bmjopen-2020-045389 (PMC8296761; doi:10.1136/bmjopen-2020-045389)
Supplement: Supplementary data [file bmjopen-2020-045389supp001.pdf]

Supplementary Material

Peer mentorship to improve self-management of osteoarthritis: A randomised feasibility trial – Supplementary Material

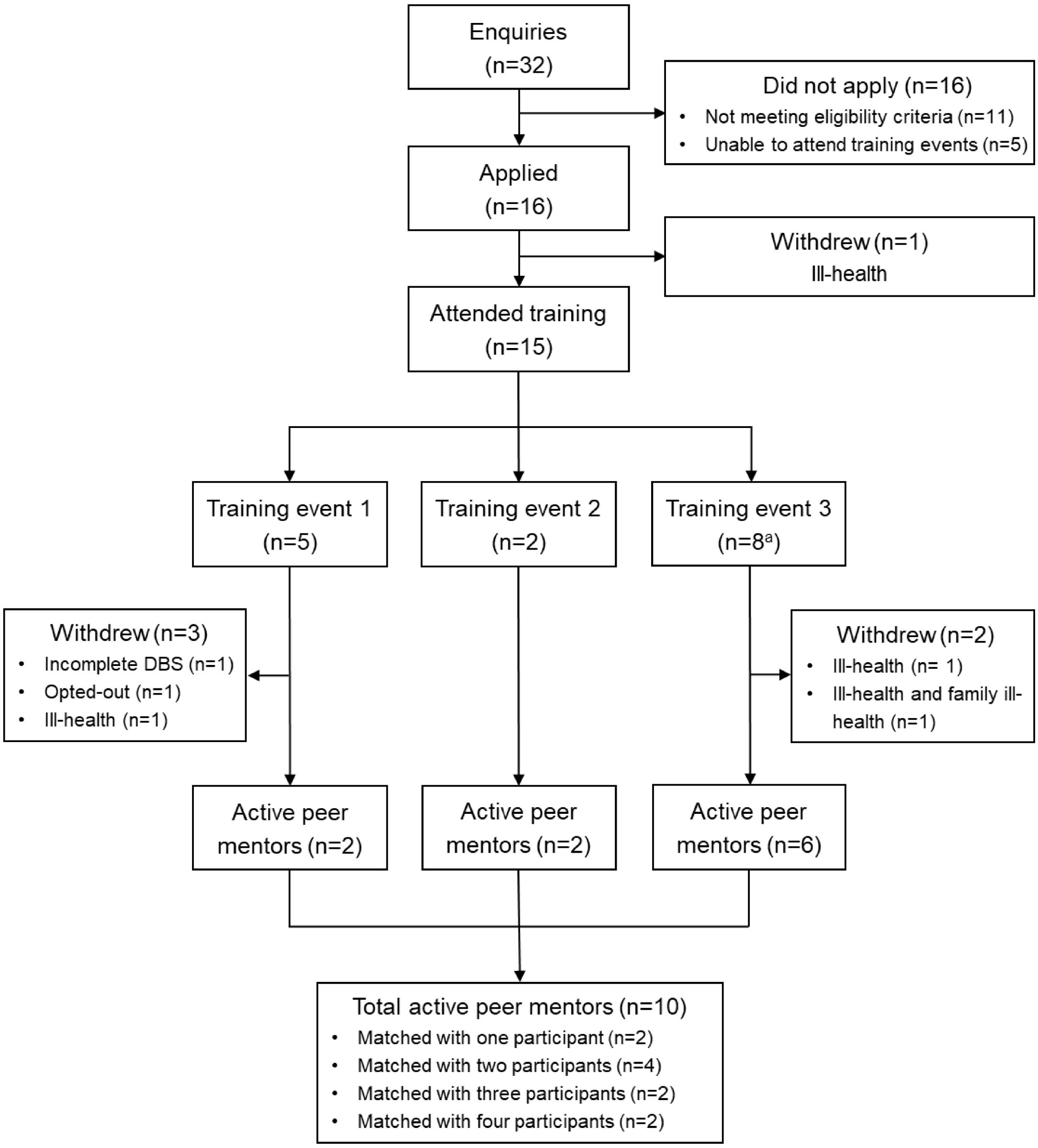

Supplementary Figure 1: Peer mentor flow diagram

[a] One trained peer mentor who attended Training event 1 also attended Training event 3 as refresher training.

DBS, Enhanced Disclosure and Barring Service check

Anderson AM, *et al. BMJ Open* 2021; 11:e045389. doi: 10.1136/bmjopen-2020-045389

Supplementary Material

Supplementary Table 1: CONSORT 2010 checklist of information to include when reporting a pilot or feasibility randomised trial in a journal or conference abstract

Item	Description	Reported on line number
Title	Identification of study as randomised pilot or feasibility trial	2
Authors	Contact details for the corresponding author	15-17
Trial design	Description of pilot trial design (eg, parallel, cluster)	28
**Methods**		
Participants	Eligibility criteria for participants and the settings where the pilot trial was conducted	29-30, 33
Interventions	Interventions intended for each group	32-35
Objective	Specific objectives of the pilot trial	25-27
Outcome	Prespecified assessment or measurement to address the pilot trial objectives	36-40
Randomisation	How participants were allocated to interventions	32
Blinding (masking)	Whether or not participants, care givers, and those assessing the outcomes were blinded to group assignment	28
**Results**		
Numbers randomised	Number of participants screened and randomised to each group for the pilot trial objectives	41-42
Recruitment	Trial status	N/A[a]
Numbers analysed	Number of participants analysed in each group for the pilot objectives	42-45
Outcome	Results for the pilot objectives, including any expressions of uncertainty	41-48
Harms	Important adverse events or side effects	45-46
Conclusions	General interpretation of the results of pilot trial and their implications for the future definitive trial	49-52
Trial registration	Registration number for pilot trial and name of trial register	53
Funding	Source of funding for pilot trial	54

Anderson AM, *et al. BMJ Open* 2021; 11:e045389. doi: 10.1136/bmjopen-2020-045389

Supplementary Material

N/A, Not applicable

[a] Not applicable as item is only applicable to conference abstracts.

Checklist from: Eldridge SM, Chan CL, Campbell MJ, et al. CONSORT 2010 statement: extension to randomised pilot and feasibility trials. *BMJ* 2016;355:i5239.

Supplementary Material

Supplementary Table 2: CONSORT 2010 checklist of information to include when reporting a pilot or feasibility trial

Section/Topic	Item No	Checklist item	Reported on page No
**Title and abstract**			
	1a	Identification as a pilot or feasibility randomised trial in the title	1
	1b	Structured summary of pilot trial design, methods, results, and conclusions	2-3
**Introduction**			
Background and objectives	2a	Scientific background and explanation of rationale for future definitive trial, and reasons for randomised pilot trial	4-5
	2b	Specific objectives or research questions for pilot trial	5
**Methods**			
Trial design	3a	Description of pilot trial design (such as parallel, factorial) including allocation ratio	5
	3b	Important changes to methods after pilot trial commencement (such as eligibility criteria), with reasons	13
Participants	4a	Eligibility criteria for participants	6
	4b	Settings and locations where the data were collected	6, 11, 15
	4c	How participants were identified and consented	6
Interventions	5	The interventions for each group with sufficient details to allow replication, including how and when they were actually administered	7-13<br>Figure 1<br>Supplementary table 3

Supplementary Material

Outcomes	6a	Completely defined prespecified assessments or measurements to address each pilot trial objective specified in 2b, including how and when they were assessed	13-17
	6b	Any changes to pilot trial assessments or measurements after the pilot trial commenced, with reasons	N/A
	6c	If applicable, prespecified criteria used to judge whether, or how, to proceed with future definitive trial	14
Sample size	7a	Rationale for numbers in the pilot trial	7
	7b	When applicable, explanation of any interim analyses and stopping guidelines	N/A
Randomisation:			
Sequence generation	8a	Method used to generate the random allocation sequence	7
	8b	Type of randomisation(s); details of any restriction (such as blocking and block size)	7
Allocation concealment mechanism	9	Mechanism used to implement the random allocation sequence (such as sequentially numbered containers), describing any steps taken to conceal the sequence until interventions were assigned	7
Implementation	10	Who generated the random allocation sequence, who enrolled participants, and who assigned participants to interventions	6-7
Blinding	11a	If done, who was blinded after assignment to interventions (for example, participants, care providers, those assessing outcomes) and how	N/A
	11b	If relevant, description of the similarity of interventions	N/A
Statistical methods	12	Methods used to address each pilot trial objective whether qualitative or quantitative	14, 17-18
**Results**			

Anderson AM, *et al. BMJ Open* 2021; 11:e045389. doi: 10.1136/bmjopen-2020-045389

Supplementary Material

Participant flow (a diagram is strongly recommended)	13a	For each group, the numbers of participants who were approached and/or assessed for eligibility, randomly assigned, received intended treatment, and were assessed for each objective	19-28<br>Figure 2
	13b	For each group, losses and exclusions after randomisation, together with reasons	20<br>Figure 2
Recruitment	14a	Dates defining the periods of recruitment and follow-up	5, 19
	14b	Why the pilot trial ended or was stopped	N/A
Baseline data	15	A table showing baseline demographic and clinical characteristics for each group	23<br>Supplementary table 7
Numbers analysed	16	For each objective, number of participants (denominator) included in each analysis. If relevant, these numbers should be by randomised group	19-28
Outcomes and estimation	17	For each objective, results including expressions of uncertainty (such as 95% confidence interval) for any estimates. If relevant, these results should be by randomised group	19-28
Ancillary analyses	18	Results of any other analyses performed that could be used to inform the future definitive trial	N/A
Harms	19	All important harms or unintended effects in each group	N/A
	19a	If relevant, other important unintended consequences	N/A
**Discussion**			
Limitations	20	Pilot trial limitations, addressing sources of potential bias and remaining uncertainty about feasibility	29-33
Generalisability	21	Generalisability (applicability) of pilot trial methods and findings to future definitive trial and other studies	32

Anderson AM, *et al. BMJ Open* 2021; 11:e045389. doi: 10.1136/bmjopen-2020-045389

Supplementary Material

Interpretation	22	Interpretation consistent with pilot trial objectives and findings, balancing potential benefits and harms, and considering other relevant evidence	28-33
	22a	Implications for progression from pilot to future definitive trial, including any proposed amendments	29-30, 32-33
**Other information**			
Registration	23	Registration number for pilot trial and name of trial registry	5
Protocol	24	Where the pilot trial protocol can be accessed, if available	35
Funding	25	Sources of funding and other support (such as supply of drugs), role of funders	34
	26	Ethical approval or approval by research review committee, confirmed with reference number	5, 34

Checklist from: Eldridge SM, Chan CL, Campbell MJ, et al. CONSORT 2010 statement: extension to randomised pilot and feasibility trials. *BMJ* 2016;355:i5239.

Anderson AM, *et al. BMJ Open* 2021; 11:e045389. doi: 10.1136/bmjopen-2020-045389

Supplementary Material

Supplementary Table 3: Key behaviour change techniques included in the osteoarthritis peer mentorship intervention

Behaviour change technique[a]	Example of implementation
1.1 Goal setting (behaviour)	The peer mentor supports the participant to set a goal of meeting a friend for coffee twice weekly.
1.2 Problem solving	The peer mentor supports the participant to analyse barriers to pacing their activities and identify strategies to help overcome the barriers.
1.4 Action planning	The peer mentor supports the participant to plan when and where they will go cycling and how long their cycle rides will last.
1.5 Review behaviour goal(s)	The peer mentor and participant review the participant's goal of not drinking any caffeinated drinks after 5.00pm and adapt it if necessary.
2.2 Feedback on behaviour	The peer mentor provides the participant with feedback on their strengthening exercise technique during their peer mentorship session.
3.1 Social support (unspecified)	The peer mentor provides information about a local walking football group.
3.3 Social support (emotional)	The peer mentor provides emotional support to help the participant to go for a walk even though they feel anxious about falling.
4.1 Instruction on how to perform the behaviour	The peer mentor advises the participant how to perform a relaxation technique.
5.1 Information about health consequences	The peer mentor explains that losing weight can help improve symptoms of osteoarthritis.
5.6 Information about emotional consequences	The peer mentor explains that using conscious breathing techniques can help people feel calmer.
6.1 Demonstration of the behaviour	The peer mentor demonstrates how to perform chair based exercises.
6.2 Social comparison	The peer mentor draws attention to how they are able to do yoga despite their osteoarthritis.
8.1 Behavioural practice/rehearsal	The peer mentor prompts the participant to practice being assertive in preparation for their doctor's appointment.
8.3 Habit formation	The peer mentor prompts the participant to carry out an exercise routine in their bedroom after they have had a shower each morning.

Supplementary Material

8.7 Graded tasks	The peer mentor supports the participant to swim 20 lengths by advising them to start with 10 lengths and then add an extra two lengths each week.
9.1 Credible source	The peer mentor provides communication in favour of eating five portions of fruit and vegetables per day.
10.4 Social reward	The peer mentor praises the participant for progressing towards their goal of getting up at 8.30am each morning.
15.1 Verbal persuasion about capability	The peer mentor tells the participant that they can do leg strengthening exercises safely despite their osteoarthritis.

[a] Behaviour change techniques are coded using the Behaviour Change Technique Taxonomy version 1 from: Michie S, Richardson M, Johnston M, et al. The Behavior Change Technique Taxonomy (v1) of 93 hierarchically clustered techniques: building an international consensus for the reporting of behavior change interventions. *Ann Behav Med* 2013 Aug;46(1):81-95. doi: 10.1007/s12160-013-9486-6.

Only key behaviour change techniques are listed. Peer mentors could incorporate additional behaviour change techniques as appropriate to the participant's individual needs and goals.

Anderson AM, *et al. BMJ Open* 2021; 11:e045389. doi: 10.1136/bmjopen-2020-045389

Supplementary Material

Supplementary Table 4: Overview of areas covered during the peer mentor training event

Day 1	Day 2
• Overview of the trial and peer mentor role<br>• Osteoarthritis key facts and self-management topics<br>• Goal setting<br>• Pacing<br>• Educational resource pack review<br>• Exercise<br>• Pain management<br>• Sleep	• Review of day 1<br>• Communication<br>• Professional boundaries<br>• Mentoring in practice (including safeguarding, disclosures and confidentiality)<br>• Visit and recording procedures<br>• Trial data collection<br>• Next steps

Anderson AM, *et al. BMJ Open* 2021; 11:e045389. doi: 10.1136/bmjopen-2020-045389

Supplementary Material

Supplementary Table 5: Estimated costs of delivering the two day peer mentor training event to five peer mentors

**Staff costs[a]**			
	Hourly rate[b]	Hours per event	Cost per event
Research Fellow (Grade 7)	£32.23	7.5	£241.73
Research Associate (Grade 6)	£26.09	15	£391.35
Volunteer Coordinator (Grade 5)	£21.79	15	£326.85
Total staff costs			£959.93
**Peer mentor costs**			
	Cost per peer mentor		Cost per event
Refreshments	£18.00		£90.00
Notebook	£2.48		£12.40
Ring binder	£2.00		£10.00
Plastic wallets	£0.67		£3.33
Printing	£10.50		£52.50
Name badge	£0.14		£0.68
Travel expenses	£13.14		£65.70
Total peer mentor costs	£46.92		£234.62
**Overall costs**			
	Cost per peer mentor		Cost per event
Overall costs	£238.91		£1,194.54

[a] Staff costs were based on the events run by the trial team members.

[b] Includes staff salary, National Insurance contributions and pension contributions, but does not include estates costs or indirect costs.

Anderson AM, *et al. BMJ Open* 2021; 11:e045389. doi: 10.1136/bmjopen-2020-045389

Supplementary Material

Supplementary Table 6: Estimated costs of delivering the OA peer mentorship intervention to one participant

**Volunteer Coordinator costs (Grade 5 hourly rate £21.79[a])**		
	Per single session	Per 5.79 sessions[b]
Hours spent supporting mentorship sessions (0.5 hours per session)	£10.90	£63.10
Additional hours spent on matching, initial session and closure (4 hours)	£15.05	£87.16
Travel expenses for initial session	£0.81	£4.72
Total Volunteer Coordinator costs	£26.76	£154.98
**Peer mentor costs**		
	Per single session	Per 5.79 sessions[b]
Honorarium	£15	£86.88
Travel expenses	£5.53	£32.01
Total peer mentor costs	£20.53	£118.89
**Overall costs**		
	Per single session	Per 5.79 sessions[b]
Overall costs	£47.29	£273.86

[a] Includes staff salary, National Insurance contributions and pension contributions, but does not include estates costs or indirect costs.

[b] 5.79 was the mean number of mentorship sessions received by participants.

Supplementary Material

Supplementary Table 7: Additional participant baseline characteristics

Characteristics	Intervention n=24	Control n=25
**Marital status,** n (%)		
Married	12 (50.0)	17 (68.0)
Divorced	6 (25.0)	2 (8.0)
Living together	0 (0.0)	1 (4.0)
Widowed	5 (20.8)	3 (12.0)
Single	1 (4.2)	2 (8.0)
**Current smokers,** n (%)	1 (4.2)	2 (8.0)
**Areas affected by arthritis**, n (%)		
Single hip	0 (0.0)	0 (0.0)
Both hips	2 (8.3)	4 (16.0)
Single knee	2 (8.3)	3 (12.0)
Both knees	8 (33.3)	9 (36.0)
Single hip and single knee	2 (8.3)	3 (12.0)
Both hips and one knee	2 (8.3)	1 (4.0)
Single hip and both knees	5 (20.8)	2 (8.0)
Both hips and both knees	3 (12.5)	3 (12.0)
**Areas affected by joint pain for > 6 weeks in the last 3 months**, n (%)		
Neck	7 (29.2)	6 (24.0)
Shoulder (right)	7 (29.2)	6 (24.0)
Shoulder (left)	7 (29.2)	7 (28.0)
Elbow (right)	0 (0.0)	2 (8.0)
Elbow (left)	0 (0.0)	2 (8.0)
Hand (right)	9 (37.5)	6 (24.0)
Hand (left)	9 (37.5)	5 (20.0)
Back	6 (25.0)	11 (44.0)
Hip (right)	10 (41.7)	10 (40.0)
Hip (left)	10 (41.7)	10 (40.0)
Knee (right)	19 (79.2)	16 (64.0)
Knee (left)	19 (79.2)	20 (80.0)

Anderson AM, *et al. BMJ Open* 2021; 11:e045389. doi: 10.1136/bmjopen-2020-045389

Foot (right)	5 (20.8)	4 (16.0)
Foot (left)	4 (16.7)	5 (16.0)
**Duration of pain in years,** mean (SD)	4.7 (5.2)	5.3 (6.2)
**Currently suffering from pain**, n (%)	23 (95.8)	25 (100.0)
**Frequency of pain**[a], n (%)		
Always	7 (30.4)	11 (44.0)
Often present	5 (21.7)	9 (36.0)
Sometimes present	10 (43.5)	3 (20.0)
Present very little	1 (4.4)	0 (0.0)
**Previous hip replacement,** n (%)	1 (4.2)	1 (4)
**Previous knee replacement(s),** n (%)	1 (4.2)	2 (8)
**Walking aid use,** n (%)	8 (33.3)	7 (28.0)
**Currently using medication for pain or other symptoms of arthritis,** n (%)	20 (83.3)	21 (84.0)
**Currently using strong opioids,** n, (%)	1 (4.2)	0 (0.0)
**Currently using over-the-counter NSAIDS,** n (%)	4 (16.7)	6 (24.0)

NSAIDS, non-steroidal anti-inflammatory drugs; SD, standard deviation.

[a] Frequency of pain intervention group n=23 because one participant reported they were not currently suffering from pain.

Supplementary Material

Supplementary Table 8: Mean resource use and mean difference between groups for the 8 week and 6 month questionnaires combined

	Intervention group mean use (n=22)	Control group mean use (n=24)	Mean difference (95% Confidence Interval)
Inpatient length of stay (nights)	0.05	0.96	-0.91 (-1.78 to -0.04)
Number of Outpatient appointments	3.05	2.92	0.13 (-1.87 to 2.13)
Number GP appointments (surgery)	2.73	2.42	0.31 (-1.47 to 2.09)
Number of GP visits (home)	0.00	0.17	-0.17 (-0.52 to 0.18)
Number of GP calls (telephone)	0.59	0.67	-0.08 (-0.70 to 0.55)
Number of GP Practice Nurse visits	1.64	0.71	0.93 (0.15 to 1.71)
Number of District Nurse visits	0.00	0.00	0.00
Number of Occupational Therapist visits	0.09	0.21	-0.12 (-0.47 to 0.24)
Number of Physiotherapist visits	1.36	1.96	-0.59 (-2.74 to 1.55)
Number of Chiropodist visits	0.64	0.75	-0.11 (-0.86 to 0.63)
Number of Pharmacist visits	2.27	1.67	0.61 (-1.04 to 2.25)
Number of NHS Direct calls	0.18	0.13	0.06 (-0.28 to 0.39)
Number of Walk-in Centre visits	0.41	0.13	0.28 (-0.43 to 1.00)
Number of Counsellor visits	0.09	0.08	0.01 (-0.24 to 0.26)

Anderson AM, *et al. BMJ Open* 2021; 11:e045389. doi: 10.1136/bmjopen-2020-045389
