# Supplementary material for: Peer mentorship to improve self-management of hip and knee osteoarthritis: a randomised feasibility trial
Source: BMJ Open. 2021 Jul 21;11(7):e045389. doi: 10.1136/bmjopen-2020-045389 (PMC8296761; doi:10.1136/bmjopen-2020-045389)
Supplement: Author's manuscript [file bmjopen-2020-045389.draft_revisions.pdf]

BMJ Open

BMJ Open is committed to open peer review. As part of this commitment we make the peer review history of every article we publish publicly available.

When an article is published we post the peer reviewers' comments and the authors' responses online. We also post the versions of the paper that were used during peer review. These are the versions that the peer review comments apply to.

The versions of the paper that follow are the versions that were submitted during the peer review process. They are not the versions of record or the final published versions. They should not be cited or distributed as the published version of this manuscript.

BMJ Open is an open access journal and the full, final, typeset and author-corrected version of record of the manuscript is available on our site with no access controls, subscription charges or pay-per-view fees (http://bmjopen.bmj.com).

If you have any questions on BMJ Open's open peer review process please email info.bmjopen@bmj.com

BMJ Open

# Peer mentorship to improve self-management of hip and knee osteoarthritis: A randomised feasibility trial



| | |
|---|---|
| Journal: | *BMJ Open* |
| Manuscript ID | bmjopen-2020-045389 |
| Article Type: | Original research |
| Date Submitted by the Author: | 29-Sep-2020 |



| | |
|---|---|
| Complete List of Authors: | Anderson, Anna; University of Leeds, School of Healthcare; University of Leeds, Leeds Institute of Rheumatic and Musculoskeletal Medicine and NIHR Leeds Biomedical Research Centre<br>Lavender, Elizabeth; University of Leeds, School of Healthcare<br>Dusabe-Richards, Esther; University of Leeds, School of Healthcare<br>Mebrahtu, Teumzghi; University of Leeds, School of Healthcare<br>McGowan, Linda; University of Leeds, School of Healthcare<br>Conaghan, Philip; University of Leeds, Leeds Institute of Rheumatic & Musculoskeletal Medicine and NIHR Leeds Biomedical Research Centre<br>Kingsbury, Sarah; University of Leeds, Leeds Institute of Rheumatic & Musculoskeletal Medicine and NIHR Leeds Biomedical Research Centre<br>Richardson, Gerry; University of York, Centre for Health Economics; NIHR Research Design Service for Yorkshire and the Humber<br>Antcliff, Deborah; University of Leeds, School of Healthcare; Northern Care Alliance NHS Group, Physiotherapy Department, Bury & Rochdale Care Organisation<br>McHugh, Gretl; University of Leeds, School of Healthcare |



| | |
|---|---|
| Keywords: | RHEUMATOLOGY, PAIN MANAGEMENT, SOCIAL MEDICINE |
| | |


SCHOLARONE™
Manuscripts

*I, the Submitting Author has the right to grant and does grant on behalf of all authors of the Work (as defined in the below author licence), an exclusive licence and/or a non-exclusive licence for contributions from authors who are: i) UK Crown employees; ii) where BMJ has agreed a CC-BY licence shall apply, and/or iii) in accordance with the terms applicable for US Federal Government officers or employees acting as part of their official duties; on a worldwide, perpetual, irrevocable, royalty-free basis to BMJ Publishing Group Ltd ("BMJ") its licensees and where the relevant Journal is co-owned by BMJ to the co-owners of the Journal, to publish the Work in this journal and any other BMJ products and to exploit all rights, as set out in our licence.*

*The Submitting Author accepts and understands that any supply made under these terms is made by BMJ to the Submitting Author unless you are acting as an employee on behalf of your employer or a postgraduate student of an affiliated institution which is paying any applicable article publishing charge ("APC") for Open Access articles. Where the Submitting Author wishes to make the Work available on an Open Access basis (and intends to pay the relevant APC), the terms of reuse of such Open Access shall be governed by a Creative Commons licence – details of these licences and which Creative Commons licence will apply to this Work are set out in our licence referred to above.*

*Other than as permitted in any relevant BMJ Author's Self Archiving Policies, I confirm this Work has not been accepted for publication elsewhere, is not being considered for publication elsewhere and does not duplicate material already published. I confirm all authors consent to publication of this Work and authorise the granting of this licence.*

For peer review only

# Peer mentorship to improve self-management of hip and knee osteoarthritis: A randomised feasibility trial

Anna M. Anderson[ab*], Elizabeth C. Lavender[a], Esther Dusabe-Richards[a], Teumzghi F. Mebrahtu[a], Linda McGowan[a], Philip G. Conaghan[b], Sarah R. Kingsbury[b], Gerry Richardson[c], Deborah Antcliff[ad], Gretl A. McHugh[a]

[a] School of Healthcare, University of Leeds, Leeds, United Kingdom

[b] Leeds Institute of Rheumatic & Musculoskeletal Medicine and NIHR Leeds Biomedical Research Centre, University of Leeds, Leeds, United Kingdom

[c] Centre for Health Economics, University of York, York, United Kingdom, and The NIHR Research Design Service for Yorkshire & the Humber, York, United Kingdom

[d] Physiotherapy Department, Bury & Rochdale Care Organisation, Northern Care Alliance NHS Group, Bury, United Kingdom

*Corresponding author at: Leeds Institute of Rheumatic & Musculoskeletal Medicine, University of Leeds, Leeds, United Kingdom,

Email address: A.Anderson@leeds.ac.uk

Word count: 4,262

Keywords: Rheumatology; Pain management; Social medicine



For peer review only - http://bmjopen.bmj.com/site/about/guidelines.xhtml

## ABSTRACT

**Objective** To determine the feasibility of conducting a randomised controlled trial (RCT) of a peer mentorship intervention to improve self-management of osteoarthritis (OA).

**Design** Six-month parallel-group non-blinded randomised feasibility trial.

**Setting** One secondary care and one primary care United Kingdom NHS Trust.

**Participants** Fifty adults aged ≥55 years old with hip and/or knee OA.

**Interventions** Participants were allocated 1:1 to the intervention or control group using an online randomisation service. Intervention group participants received usual care (information resources) and up to eight community-based self-management support sessions delivered by a peer mentor (trained volunteer with hip and/or knee OA). Control group participants received usual care only.

**Outcome measures** Key feasibility outcomes were participant and peer mentor recruitment and attrition, intervention completion and the sample size required for a definitive RCT. Based on these feasibility outcomes, four success criteria for proceeding to a definitive RCT were pre-specified. Patient-reported outcomes were collected via questionnaires at baseline, eight weeks and six months.

**Results** Ninety-six individuals were screened, 65 were eligible and 50 were randomised (25 per group). Of the 24 participants who commenced the intervention, 20 completed it. Four participants did not complete the six-month questionnaire. Twenty-one individuals were eligible for the peer mentor role, 15 were trained and five withdrew prior to being matched with a participant. No intervention-related harms occurred. Allowing for 20% attrition, the sample size required for a definitive RCT




For peer review only - http://bmjopen.bmj.com/site/about/guidelines.xhtml

was calculated as 170 participants. The intervention group showed improvements in self-management compared to the control group.

**Conclusions** The feasibility outcomes achieved the pre-specified criteria for proceeding to an RCT. The exploratory analyses suggest peer mentorship may improve OA self-management. An RCT of the OA peer mentorship intervention is therefore warranted with minor modifications to the intervention and trial procedures.

**Trial registration** ISRCTN:50675542

**Funding** National Institute for Health Research (NIHR)

## STRENGTHS AND LIMITATIONS OF THIS STUDY

- This randomised feasibility trial involved the development of a novel peer mentorship intervention to improve self-management of hip and knee osteoarthritis (OA).
- Extensive Patient and Public Involvement ensured the intervention and trial procedures were tailored to the needs of individuals with OA.
- A comprehensive range of feasibility outcomes were assessed, providing valuable information for designing a future definitive randomised controlled trial.
- Validated patient-reported outcome measures were administered; however, the results must be interpreted cautiously because the trial was not powered to detect statistically significant differences.
- A key limitation was that the majority of participants were recruited through a physiotherapy service and hence had already received some self-management support.

For peer review only



For peer review only - http://bmjopen.bmj.com/site/about/guidelines.xhtml

## INTRODUCTION

Osteoarthritis (OA) is one of the most prevalent musculoskeletal conditions, with hip and knee OA affecting over 300 million individuals worldwide.[1] Furthermore, the prevalence of OA is increasing due to the ageing population and rising obesity levels.[2] Individuals with OA often experience severe pain, impaired function and reduced quality of life.[3] Osteoarthritis can have a profound psychosocial impact,[4] and results in substantial economic burden.[5]

National and international guidelines emphasise that patient education, self-management strategies and exercise are core elements for managing OA.[6] However, implementation of OA guidelines is currently poor.[7] Patients report receiving insufficient information about OA management,[8] and having a limited understanding of the condition.[4, 9] This negatively impacts patients' health behaviours.[4, 9] A need for tailored interventions to support OA self-management has therefore been highlighted.[10]

Peer support interventions are an established approach for supporting chronic condition self-management.[11, 12] Various peer support models have been described, such as peer-led group programmes and peer mentorship.[13] The latter involves a trained individual with a particular health condition (the 'peer mentor') providing one-to-one support to another individual with the same condition.[13] This approach is likely to be particularly valuable for individuals with OA due to the heterogeneous nature of the condition, which means tailored support is crucial.[10, 14]

For peer review only

Previous studies have highlighted the value of OA interventions incorporating peer support.[15] However, no previous studies have explored an OA peer mentorship intervention. Although such an intervention offers multiple potential benefits, peer mentorship interventions can present feasibility/acceptability issues, such as high peer mentor attrition.[16] Therefore, this feasibility trial aimed to develop and trial a peer mentorship intervention to improve OA self-management. Its key objectives were to determine the feasibility of conducting a randomised controlled trial (RCT) of the OA peer mentorship intervention in terms of: the feasibility/acceptability of the intervention and trial procedures; participant recruitment and retention; questionnaire completion rates; generating the sample size required for a definitive RCT; and estimating the intervention costs. Additionally, the potential impact of the intervention on patient-reported outcomes and resource use was explored.

**METHODS**

This was a six-month parallel-group randomised feasibility trial. A nested qualitative study was included (reported elsewhere). The trial was prospectively registered (ISRCTN:50675542) and conducted between 1st September 2017 and 16th February 2020. Ethical approval was gained from the Greater Manchester South Research Ethics Committee (Reference 17/NW/0238). The trial is reported according to the CONSORT 2010 extension for randomised pilot and feasibility trials,[17] (online supplementary tables 1 and 2).



For peer review only - http://bmjopen.bmj.com/site/about/guidelines.xhtml

**Trial procedures and participants**

Recruitment and consent

Potential participants were identified from rheumatology and orthopaedic clinics of one secondary care NHS Trust and physiotherapy clinics and electronic records of one primary care NHS Trust. Both are large Trusts in Northern England. The initial approach was made via clinical staff during clinic appointments or through an invitation letter. Individuals interested in participating were provided with further details about the trial and screened for eligibility by a researcher at their clinic appointment or via telephone.

All participants provided written informed consent at their clinic appointment or during a baseline visit from a researcher. The latter took place in the participant's home or another private location of the participant's choice.

Eligibility criteria

Inclusion criteria were: 1) aged ≥55 years old, and 2) clinician-confirmed diagnosis of hip and/or knee OA.[18] Exclusion criteria were: 1) presence of inflammatory arthritis (including gout and rheumatoid arthritis), 2) serious health conditions that would prevent participation, and 3) listed for hip/knee replacement.

Sample size



For peer review only - http://bmjopen.bmj.com/site/about/guidelines.xhtml

Recommendations for pilot studies suggest 20 participants per arm is acceptable assuming at worst a small effect size (Cohen's d=0.2) for a continuous outcome and 80% power.[19] Allowing for 20% attrition,[20] the sample size was set at 25 participants per arm.

Randomisation

Participants were randomised to the intervention or control group with 1:1 allocation using the Sealed Envelope online randomisation service.[21] This was set up by an independent statistician to generate blocked randomisation with varying block lengths, stratified according to educational level. The researcher performing the randomisation was unable to access the allocation codes. Given the nature of the intervention, participant blinding was not possible.

Intervention group

Intervention group participants received usual care and the OA peer mentorship intervention. Usual care consisted of information resources (an OA booklet from Arthritis Research UK (now Versus Arthritis),[22] and a handout about local services/support groups/activities). The intervention group participants received the information resources during their initial mentorship session and were offered the opportunity to discuss the resources with their peer mentor.

Control group

For peer review only



For peer review only - http://bmjopen.bmj.com/site/about/guidelines.xhtml

Control group participants received usual care only. The control group participants received the information resources during their baseline researcher visit and were offered the opportunity to discuss the resources with the researcher.

**OA peer mentorship intervention**

Intervention development

For peer review only

The OA peer mentorship intervention and associated educational resources were developed based on:

- A rapid review of published research exploring the development and implementation of peer mentorship interventions to support chronic condition self-management
- Guidelines on self-management and OA from organisations such as the United Kingdom (UK) National Institute for Health and Care Excellence (NICE) and the UK charity Versus Arthritis
- The 'Staying Connected Programme' – an arthritis self-management programme previously run by Arthritis Care Northern Ireland (now Versus Arthritis Northern Ireland),[23]

The provisional intervention and educational resources were refined through expert review sessions conducted with: study Patient and Public Involvement (PPI) members (n=2); other individuals with OA (n=5); health professionals (n=4); voluntary/community organisation representatives (n=4); and researchers (n=2).



For peer review only - http://bmjopen.bmj.com/site/about/guidelines.xhtml

Peer mentor eligibility and recruitment

Peer mentors were trained volunteers aged ≥50 years old with hip and/or knee OA. Multiple approaches were used to recruit peer mentors, including printed/social media advertisements and sharing trial information at local support/activity groups.

Potential peer mentors were asked to complete an application form, supply two references and take part in a telephone interview with the Volunteer Coordinator. Those assessed as suitable were invited to attend a compulsory two-day training event and required to complete enhanced Disclosure and Barring Service checks.

Peer mentor training

Three training events were held due to varying peer mentor availability and staggering of the mentor recruitment. The first training event was provided by two external facilitators from Arthritis Care Northern Ireland and a trial team member (physiotherapist specialising in activity pacing/chronic pain management). The remaining events were provided by three trial team members (Volunteer Coordinator, the physiotherapist who facilitated the first training event and another musculoskeletal physiotherapist). Feedback from the first event was used to refine the content/delivery of the subsequent events.

The training events involved presentations and interactive activities covering OA self-management topics, mentorship skills and the practicalities of the peer mentor role (online supplementary table 3).

For peer review only

## Intervention delivery

The OA peer mentorship intervention involved up to eight one-hour self-management support sessions delivered by a peer mentor. The sessions took place approximately weekly in the participant's home or another private location of their choice. A person-centred approach was emphasised throughout.[24, 25] Therefore, the number of sessions and scheduling were flexible.

Participant/mentor matching was undertaken by the Volunteer Coordinator and the researcher who completed the baseline researcher visit. The initial intention was to base matching on location, gender preference, age and OA site(s). However, due to peer mentors' preferences and discrepancies in the availability of intervention group participants and trained peer mentors, these criteria were relaxed.

The Volunteer Coordinator attended the start of each initial session to introduce the participant and peer mentor; remind them of the trial and intervention aims; and answer questions. The remainder of the initial session and all subsequent sessions were undertaken by the mentor alone. The Volunteer Coordinator contacted the participant and peer mentor following the initial session to check they were happy to continue with subsequent sessions with their mentorship match.

During the sessions, the peer mentor provided guided support covering a combination of the following topics:



For peer review only - http://bmjopen.bmj.com/site/about/guidelines.xhtml

- Core topics: Learning about OA; Goal setting; Pacing; Muscle strengthening; and Getting connected with friends/services
- Optional topics: Getting active; Effective communication; Eating well; Positive thinking; Breathing and relaxation; Sleep quality; and Pain management

In line with a person-centred approach, the choice and order of topics covered was flexible and participant-led. However, peer mentors were encouraged to cover all core topics at least once. Peer mentors recorded a 'session summary' following each session, detailing the topics covered during that session, any challenges encountered and their reflections on the progress made.

The peer mentors received a comprehensive educational resource pack covering all the core and optional topics. The pack also included handouts to pass onto participants.

## Data collection and analysis

### Feasibility outcomes

This trial's primary focus was on assessing the feasibility outcomes specified in table 1. Four associated success criteria for proceeding to a definitive RCT were pre-specified based on relevant guidance,[26] (table 1).



For peer review only - http://bmjopen.bmj.com/site/about/guidelines.xhtml

**Table 1: Feasibility outcomes and success criteria for proceeding to a definitive randomised controlled trial**

| Feasibility outcome | Details of feasibility outcome | Success criterion |
|---|---|---|
| Peer mentor recruitment rate | Number of peer mentors trained divided by the number individuals who were eligible for the peer mentor role | 1. Demonstration that the intervention can be delivered in practice and is acceptable to participants and peer mentors[1] |
| Peer mentor attrition rate | Number of peer mentors who were not matched with a participant divided by the number of peer mentors trained | |
| Intervention completion rate | Number of participants who completed at least three peer mentorship sessions divided by the number of participants who commenced the intervention | |
| Participant recruitment rate | Number of participants randomised divided by the number of individuals who were eligible to participate | 2. ≥60% of eligible individuals recruited |
| Attrition rate overall and in each group | Number of participants who did not complete the 6 month questionnaire divided by the number of participants randomised | 3. <20% participant attrition with no evidence of attrition bias |
| Sample size required for a definitive RCT | Sample size calculation using the feasibility trial PIH scores assuming: 5% significance level; minimum power of 0.8; and 1:1 allocation ratio | 4. Calculation of a sample size that is achievable in a main trial |
| Questionnaire completion rate | Number of questionnaires completed divided by the number of participants provided with the questionnaires | None specified for these feasibility outcomes |
| Intervention fidelity | Content analysis of the mentors' session summaries | |
| Cost of delivering the mentor training event | Estimation based on the events delivered by the trial team members accounting for: staff salary, National Insurance and pension contributions; resources; refreshments; and peer mentor travel expenses; but not venue hire, estates and indirect costs | |
| Cost of delivering the intervention | Estimation based on the Volunteer Coordinator and peer mentor costs, excluding the training costs | |

For peer review only

PIH scale, Revised 12-Item Partners In Health scale; RCT, randomised controlled trial.

[1] No minimum/maximum rates were pre-specified as acceptable for this success criterion.

Patient-reported outcomes

Patient-reported outcomes were assessed using paper self-report questionnaires administered at baseline, eight weeks and six months. The baseline questionnaires were administered by a researcher or the Volunteer Coordinator. The majority of baseline questionnaires were administered after the participant had been informed of their group allocation. This approach was chosen for logistical reasons, primarily related to the fluctuating availability of trained peer mentors and the need to avoid a significant delay between intervention group participants completing the baseline questionnaire and commencing the intervention. The eight-week and six-month questionnaires were administered and returned via post. Non-responders were followed up with a second questionnaire posting two weeks later and a telephone call four weeks later.

The questionnaires covered participants' sociodemographic and clinical characteristics and included validated tools assessing the biomedical/psychosocial outcomes specified below.

Provisional primary outcome measure



For peer review only - http://bmjopen.bmj.com/site/about/guidelines.xhtml
For peer review only

- Revised 12-Item Partners in Health (PIH) Scale: 12-item scale that assesses chronic condition self-management.[27] Each item is scored on a 9-point Likert scale (0-8). Scores are summed to give a total score (0-96). Higher scores indicate higher self-management knowledge/behaviours.

Provisional secondary outcome measures

- Multidimensional Scale of Perceived Social Support (MSPSS): 12-item scale that assesses perceived social support from a significant other, family and friends.[28] Each item is scored on a 7-point Likert scale (1-7) and the mean for all items is calculated. Higher scores indicate greater perceived social support.
- Western Ontario and McMaster's University Osteoarthritis Index Likert version (WOMAC® 3.1): 24-item disease-specific scale with three subscales that assess pain (5 items), stiffness (2 items) and physical function (17 items).[29] Each item is scored on a 5-point Likert scale (0-4). Scores are summed to give a total score (0-96) and subscale scores (pain: 1-20; stiffness: 0-8; physical function: 0-68). Higher scores indicate more severe problems.
- 8-item Arthritis Self-Efficacy Scale English Version (ASES-8): 8-item scale that assesses self-efficacy for managing arthritis symptoms.[30-32] Each item is scored on a 10-point Likert scale (1-10) and the mean for all items is calculated. Higher scores indicate greater self-efficacy.
- Hospital Anxiety and Depression Scale (HADS): 14-item scale with two subscales that assess symptoms of anxiety (7 items) and depression (7 items).[33] Each item is scored on a 4-point Likert scale (0-3). Scores are

For peer review only



For peer review only - http://bmjopen.bmj.com/site/about/guidelines.xhtml

summed to give a total score (0-42) and subscale scores (0-21). Higher scores indicate more severe symptoms.

- EQ-5D-5L: Descriptive system and visual analogue scale (VAS) that assess general health status.[34] The descriptive system includes five items, each scored on a 5-point Likert scale (1-5). The scores can be converted into an index value anchored at 0 (a state equivalent to dead) and 1 (full health). The VAS consists of a single score on a scale from 0 (worst health imaginable) to 100 (best health imaginable).

The eight-week and six-month questionnaires included sections on healthcare and community resource use, adapted from the Client Services Receipt Inventory (CSRI).[35]

Missing data were addressed in line with guidance for the relevant outcome measurement tool. The quantitative questionnaire responses were analysed using STATA (version 15). Given the trial was not powered to detect statistically significant differences the analyses were predominantly descriptive. However, exploratory analyses of the outcome measure data and healthcare resource use data were performed. The outcome scores were tested for normality at the 5% significance level.[36] Between-group comparisons were made using ANCOVA models and, where the scores were significantly skewed, quantile regression models were used.[37]

**Patient and Public Involvement**

For peer review only



For peer review only - http://bmjopen.bmj.com/site/about/guidelines.xhtml

Patient and Public Involvement (PPI) played a key role in this trial as follows:

- During the trial development stage, six individuals with OA were consulted for research ideas and eight members of a local musculoskeletal PPI group participated in a group discussion. These patient representatives emphasised that one-to-one mentorship would be preferable to a group-based approach and felt the questionnaire burden was appropriate.
- Three patient representatives became ‘study PPI members’ and assisted with the conduct of the study, reviewing lay materials and finalising the dissemination plans. These PPI members were also invited to attend peer mentorship training sessions.
- Two study PPI members and five other individuals with OA helped refine the provisional intervention and educational resources through expert review sessions. A key change resulting from these sessions was the addition of more images and infographics to the education resources.
- The peer mentors were all trained volunteers with hip and/or knee OA. Peer mentors were encouraged to give regular informal feedback about their involvement in the trial, as well as being invited to participate in a nested qualitative study (reported elsewhere).

## RESULTS

### Feasibility outcomes



For peer review only - http://bmjopen.bmj.com/site/about/guidelines.xhtml

Participant flow and intervention completion

Participant recruitment took place between 22nd November 2018 and 30th May 2019. The final questionnaire was completed on the 16th February 2020. At least 345 individuals were approached (figure 1). Ninety-six individuals were screened, of whom 65 were eligible. The commonest reason for ineligibility was being listed for joint replacement (n=8). Fifty individuals were randomised. Therefore, the participant recruitment rate was 77%. The commonest reason for declining participation was insufficient time/other commitments (n=7). Most participants were recruited through the primary care physiotherapy department (n=44).

One intervention group participant withdrew due to ill-health prior to completing the baseline questionnaire and commencing the intervention. Of the 24 participants who commenced the intervention, 20 completed at least three mentorship sessions (figure 1). The intervention completion rate was therefore 83%. Reasons for discontinuing the intervention largely related to participants feeling the intervention was not relevant to their needs, for example, due to having mild OA symptoms and/or feeling they did not require additional self-management support. The mean number of mentorship sessions received was 5.79 (SD=2.25; median=7). No harms/unintended effects related to the intervention occurred.

One intervention participant who discontinued the intervention after two mentorship sessions withdrew (no reason provided). One additional intervention participant who discontinued the intervention after two mentorship sessions did not complete the follow-up questionnaires. One control group participant did not complete the follow-

For peer review only



For peer review only - http://bmjopen.bmj.com/site/about/guidelines.xhtml

up questionnaires. A family member of this control group participant returned his six-month questionnaire indicating he had died. The attrition rates were therefore: 8% overall; 12% in the intervention group; and 4% in the control group.

Questionnaire completion

Amongst the participants who were provided with the questionnaires, the questionnaire completion rate for both groups was 100% at baseline and 96% at eight weeks and six months (figure 1). Minor issues were noted with the resource use sections. Some participants duplicated details, for example, by recording the same physiotherapy appointments under 'Hospital services' and 'Services outside the hospital'. Additionally, the 'day activities' reported by participants largely related to their hobbies, such as yoga and dancing, rather than social/community support activities.

Peer mentor flow and characteristics

Peer mentor recruitment took place between May 2018 and January 2019. Thirty-two individuals enquired about the role, of whom 21 were eligible (online supplementary figure 1). Five eligible individuals were unable to attend the training events, one withdrew due to ill-health and 15 were trained. The peer mentor recruitment rate was therefore 71%. Five trained mentors withdrew prior to being matched, most commonly due to ill-health (n=3). The peer mentor attrition rate was therefore 33%. The most successful peer mentor recruitment approach was a local community magazine advertisement, which led to recruitment of five active peer mentors.



For peer review only - http://bmjopen.bmj.com/site/about/guidelines.xhtml

Nine active peer mentors were female and one was male. Their mean age was 68 years old (SD=5.5; range=57-75). All had been living with hip and/or knee OA for at least three years. Nine were retired, although three had only recently stopped work, and one worked full-time. Each active peer mentor supported between one and four participants (online supplementary figure 1).

Session summary data

The session summaries indicated the following topics were covered by almost all participant/mentor dyads and were the most frequently covered overall: Learning about OA; Goal setting; Pacing; Muscle strengthening; and Pain management. In addition, Getting active and Eating well were frequently covered.

The least frequently covered topics were: Getting connected with friends/services; and Effective communication. Correspondingly, these topics were covered by only six and eight dyads respectively. Positive thinking and Sleep quality were also rarely covered.

Intervention costs

The estimated cost of delivering the training event to five peer mentors (mean number of mentors per event) was £239 per mentor (online supplementary table 4). The estimated cost of delivering the intervention based on the mean number of 5.79 mentorship sessions was £274 per participant (online supplementary table 5).



For peer review only - http://bmjopen.bmj.com/site/about/guidelines.xhtml

Sample size calculation

Based on the assumptions stated in table 1, between-groups variance and within-groups variance of the PIH scale at six months of 369.1 and 6237.1 respectively and detecting a difference in the PIH scale of 4.4 (effect size at six months), the sample size required for a definitive RCT was calculated as 136 participants (68 per group). Retaining the same attrition rate as for the feasibility trial (20%) would give a required sample size of 170 (85 per group).

**Patient-reported outcomes**

Participant baseline characteristics

Table 2 provides a summary of participants' baseline sociodemographic and clinical characteristics. Additional details are available in online supplementary table 6.

**Table 2: Participant baseline characteristics**

| Characteristics | Intervention (n=24) | Control (n=25) |
|---|---|---|
| Age in years, mean (SD) | 70.0 (8.6) | 69.3 (8.1) |
| Men, n (%) | 10 (41.7) | 4 (16.0) |
| Ethnicity, white, n (%) | 21 (87.5) | 24 (96.0) |
| Further Education[a], n (%) | 15 (62.5) | 15 (60.0) |
| Employed, n (%) | 7 (29.2) | 5 (20.0) |
| Retired, n (%) | 17 (70.8) | 20 (80.0) |
| Body mass index in kg/m$^2$, mean (SD) | 27.5 (6.3) | 28.5 (5.5) |



For peer review only - http://bmjopen.bmj.com/site/about/guidelines.xhtml

| | | |
|---|---|---|
| Duration of arthritis diagnosis in years, mean (SD) | 4.7 (5.2) | 5.3 (6.2) |
| At least one hip affected by arthritis, n (%) | 14 (58.3) | 13 (52.0) |
| At least one knee affected by arthritis, n (%) | 22 (91.7) | 21 (84.0) |
| VAS for current pain due hip/knee arthritis, mean (SD) | 52.8 (26.8) | 48.6 (26.3) |
| VAS for ability to cope with/manage arthritis in general, mean (SD) | 66.2 (21.8) | 64.4 (21.8) |
| Number of joints affected by pain for > 6 weeks in the last 3 months, median (IRQ) | 5 (4) | 3 (3) |
| Number of comorbidities[b], n (%) | | |
| 0 | 8 (33.3) | 6 (24.0) |
| 1 | 5 (20.8) | 10 (40.0) |
| 2 | 5 (20.8) | 3 (12.0) |
| ≥3 | 6 (25.0) | 6 (24.0) |

SD, standard deviation; VAS, Visual Analogue Scale (0-100mm)

[a] Further education was defined as any formal education undertaken once the participant was aged over 16 years old

[b] Comorbidities include: diabetes; asthma; bronchitis; gastrointestinal problems; angina/heart problems; high blood pressure; depression; and anxiety.

Provisional primary outcome

The mean PIH scores at baseline were 73.9 and 76.5 for the intervention and control groups respectively (table 3). After eight weeks, the mean PIH score improved in the intervention group (mean change=2.6; SD=13.2), and deteriorated in the control group (mean change= -7; SD=12.3). Thus, the intervention group participants appeared to have better self-management knowledge/behaviours than the control

For peer review only

group participants at eight weeks (effect size=8.3; 95% CI: 2.2 to 14.4) (table 4). At six months, the intervention group's mean PIH score was similar to the group's score at eight weeks, whilst the control group's mean PIH score increased but remained lower than at baseline. Overall, the intervention group had higher PIH scores than the control group after six months (effect size=4.4; 95% CI: -2.8 to 11.6) (table 4).

For peer review only



For peer review only - http://bmjopen.bmj.com/site/about/guidelines.xhtml

**Table 3: Outcome measurement scores at baseline, 8 weeks and 6 months**

| Outcome measure | Baseline means (SD) | | 8 week means (SD) | | 6 month means (SD) | |
|---|---|---|---|---|---|---|
| | Intervention (n=22) | Control (n=24) | Intervention (n=22) | Control (n=24) | Intervention (n=22) | Control (n=24) |
| PIH scale | 73.9 (16.3) | 76.5 (14.3) | 76.5 (14.5) | 69.5 (10.6) | 76.0 (13.4) | 73.0 (15.0) |
| MSPSS | 5.3 (1.1) | 5.7 (0.98) | 4.9 (1.6) | 5.2 (1.6) | 4.9 (1.3) | 5.4 (1.4) |
| WOMAC Total[a] | 32.6 (18.8) | 41.9 (19.5) | 34.0 (17.8) | 43.5 (19.4) | 34.7 (18.5) | 41.3 (17.8) |
| WOMAC Pain[a] | 6.8 (4.5) | 9 .0 (4.2) | 7.5 (3.9) | 9.6 (4.1) | 7.7 (4.0) | 8.9 (3.2) |
| WOMAC Stiffness[a] | 3.7 (1.6) | 4.3 (1.9) | 3.3 (1.3) | 4.3 (1.6) | 3.4 (1.3) | 4.0 (1.7) |
| WOMAC Function[a] | 22.1 (14.0) | 28.5 (14.6) | 23.2 (13.2) | 29.6 (15.3) | 23.6 (14.8) | 28.3 (13.5) |
| ASES-8 | 6.0 (1.9) | 5.9 (1.7) | 5.5 (2.2) | 6.5 (2.4) | 6.3 (1.6) | 5.3 (2.5) |
| HAD Total[a] | 9.4 (4.4) | 11.3 (7.2) | 10.5 (5.7) | 12.7 (8.3) | 10.7 (6.2) | 12.8 (8.4) |
| HADS Anxiety[a] | 5.3 (3.1) | 6.7 (4.3) | 6.3 (3.4) | 7.3 (4.8) | 5.9 (3.9) | 7.5 (4.9) |
| HADS Depression[a] | 4.1 (2.0) | 4.6 (3.6) | 4.3 (3.1) | 5.4 (4.0) | 4.8 (2.7) | 5.3 (4.0) |
| EQ-5D-5L Index[bc] | 0.723 (0.139) | 0.645 (0.170) | 0.691 (0.099) | 0.647 (0.249) | 0.687 (0.088) | 0.647 (0.183) |
| EQ-5D-5L VAS[c] | 70 (15.0) | 75 (20.0) | 70 (25.0) | 70 (22.5) | 75 (20.0) | 70 (23.5) |

ASES-8, 8-item Arthritis Self-Efficacy Scale English version; HADS, Hospital Anxiety and Depression Scale; MSPSS, Multidimensional Scale of Perceived Social Support; PIH scale, Revised 12-Item Partners in Health scale; VAS, Visual Analogue Scale; WOMAC, Likert version of the Western Ontario and McMaster's University Osteoarthritis Index.

[a] Higher scores indicate more severe problems.

For peer review only



For peer review only - http://bmjopen.bmj.com/site/about/guidelines.xhtml

[b] EQ-5D-5L Index value control group n=23 due to missing data.

[c] Median (inter-quartile range).

**Table 4: Outcome measurements score changes and effect of the intervention at 8 weeks and 6 months**

| Outcome measure | Changes at 8 weeks | | Effect at 8 weeks, mean (95% CI)[c] | Changes at 6 months | | Effect at 6 months, mean (95% CI)[c] |
|---|---|---|---|---|---|---|
| | Intervention | Control | | Intervention | Control | |
| PIH scale | 2.6 (13.2) | -7.0 (12.3) | 8.3 (2.2-14.4) | 2.1 (10.6) | -3.5 (16.6) | 4.4 (-2.8-11.6) |
| MSPSS | -0.28 (1.4)[d] | -0.25 (1.2)[d] | 0.0 (-0.80-0.80)[e] | -0.42 (0.93)[d] | -0.06 (0.6)[d] | -0.40 (-0.84-0.03)[e] |
| WOMAC Total[a] | 3.0 (19)[d] | 5.0 (22)[d] | -2.6 (-10.2-4.9)[e] | 2.1 (8.6) | -0.59 (19.1) | -0.65 (-8.9-7.6) |
| WOMAC Pain[a] | 0.64 (2.6) | 0.63 (3.7) | -0.75 (-2.5-1.0) | 0.91 (2.4) | -0.09 (3.8) | 0.06 (-1.6-1.7) |
| WOMAC Stiffness[a] | -0.36 (1.5) | -0.04 (1.1) | -0.59 (-1.3-0.08) | -0.27 (1.8) | -0.29 (1.2) | -0.31 (-1.1-0.46) |
| WOMAC Function[a] | 1.1 (7.8) | 1.0 (10.4) | -1.3 (-6.7-4.1) | 1.5 (7.8) | -0.21 (15.4) | -0.8 (-7.7-6.0) |
| ASES-8 | 0.48 (1.6) | -0.04 (2.25) | 0.64 (-0.51-1.8) | 0.69 (3.0)[d] | 0.0 (3.3)[d] | 0.34 (-1.3-1.9)[e] |
| HADS Total[a] | 1.12 (3.8) | 1.38 (4.5) | -0.27 (-2.8-2.3) | 1.33 (3.4) | 1.5 (4.3) | -0.01 (-2.4-2.4) |
| HADS Anxiety[a] | 0.95 (2.8) | 0.58 (2.1) | 0.26 (-1.2-1.7) | 0.59 (2.4) | 0.79 (2.6) | -0.23 (-1.8-1.3) |
| HADS Depression[a] | 0.20 (2.4) | 0.79 (3.4) | -0.74 (-2.5-1.0) | 0.74 (1.6) | 0.67 (2.5) | 0.03 (-1.3-1.3) |
| EQ-5D-5L Index[b] | -0.057 (0.20) | 0 (0.12) | -0.06 (-0.18-0.06) | -0.053 (0.14) | 0 (0.19) | -0.04 (-0.12-0.04) |
| EQ-5D-5L VAS | -2.5 (20)[d] | 0 (10)[d] | 0 (-10.8-10.8)[e] | 0 (20)[d] | -5 (21)[d] | 6.7 (-4.6-18.0)[e] |

For peer review only



For peer review only - http://bmjopen.bmj.com/site/about/guidelines.xhtml

ASES-8, 8-item Arthritis Self-Efficacy Scale English version; CI, Confidence interval; HADS, Hospital Anxiety and Depression Scale; MSPSS, Multidimensional Scale of Perceived Social Support; PIH scale, Revised 12-Item Partners in Health scale; WOMAC, Likert version of the Western Ontario and McMaster's University Osteoarthritis Index.

[a] Higher scores indicate more severe problems.

[b] EQ-5D-5L Index value control group n=23 due to missing data.

[c] ANCOVA models adjusted for baseline outcomes.

[d] Median (inter-quartile range).

[e] Median (95% confidence intervals).

For peer review only

Provisional secondary outcomes

There were no significant between-group differences for any of the secondary outcomes at eight weeks or six months. However, the mean WOMAC and HADS scores were lower in the intervention group than the control group at baseline and both follow-up time-points.

Resource use

Healthcare resource use between baseline and six months was largely similar between groups. The main differences were more overnight hospital stays in the control group (mean difference between groups= -0.91; 95% CI: -1.78 to -0.04), and more GP Practice Nurse visits in the intervention group (mean difference between groups=0.93; 95% CI: 0.15 to 1.71) (online supplementary table 7). No participants reported attending a day centre. Two intervention group participants and four control group participants reported receiving help with daily activities.

For peer review only

## DISCUSSION

This feasibility trial aimed to develop and trial a peer mentorship intervention to improve OA self-management. All four pre-specified success criteria for proceeding to a definitive RCT (table 1) were achieved. The peer mentor recruitment rate (71%), peer mentor attrition rate (33%) and intervention completion rate (83%) all compare favourably with previous peer mentorship studies.[16, 38-40] This suggests the OA peer mentorship intervention can be delivered in practice and is acceptable to



For peer review only - http://bmjopen.bmj.com/site/about/guidelines.xhtml

participants and peer mentors. However, some participants discontinued the intervention due to having mild OA symptoms and feeling they did not need self-management support. This could be overcome by specifying a minimum symptom severity threshold and/or a maximum arthritis-related self-efficacy threshold in the eligibility criteria to prevent inclusion of individuals who have mild symptoms and/or already feel confident about managing their symptoms.

The participant recruitment rate was 77% and hence well above the pre-specified minimum threshold of 60%. However, this recruitment rate did not account for individuals lost prior to screening. The overall participant attrition rate at 6 months was 8% and hence well below the pre-specified maximum threshold of 20%. This suggests that additional strategies to improve participant recruitment and retention would not be needed for a future RCT. Allowing for 20% attrition, the sample size required for a definitive RCT was calculated as 170 participants (85 per group). Assuming each peer mentor supports two participants, this would require 43 mentors. This target RCT sample size is achievable. However, only ten active peer mentors were recruited during this trial. Additional strategies would therefore be needed to recruit sufficient mentors. These could include using multiple recruitment sites, recruiting mentors over a longer time-period and optimising recruitment through media outlets, such as magazine advertisements.

The findings for the remaining feasibility outcomes were also encouraging. The questionnaire completion rates were consistently high (96%-100%). However, minor modifications to the resource use sections would be beneficial to avoid participants duplicating details/reporting unnecessary details. The session summary data



For peer review only - http://bmjopen.bmj.com/site/about/guidelines.xhtml

indicated intervention fidelity was good overall, with most participant/mentor dyads covering four of the five core topics. The remaining core topic, Getting Connected with friends/services, was only covered by six dyads. This may be because the participants already had good social support networks, as suggested by their relatively high MSPSS scores. Additionally, most dyads covered the optional topic of Pain management. Therefore, changing Getting connected to an optional topic and Pain management to a core topic warrants consideration.

The estimated costs of training peer mentors and delivering the intervention were £239 per mentor and £274 per participant respectively. The training costs would have been lower if fewer training events had been run with more mentors per event. This was not possible due to varying peer mentor availability and staggering of the mentor recruitment. A similar issue was noted in a study of peer mentorship for people with advanced cancer,[39] and would need accounting for in a future RCT.

The exploratory analyses suggest the OA peer mentorship intervention may improve self-management knowledge/behaviours (table 4). However, no significant effects of the intervention on other outcomes, such as pain and arthritis-related self-efficacy, were observed. A Cochrane review identified low to moderate quality evidence indicating self-management education programmes for people with OA do not result in any clinically meaningful benefits.[41] Despite this, the authors concluded that trials investigating alternative self-management support approaches, particularly those involving tailored support, are warranted. One such approach is the Staying Connected Programme – a tailored one-to-one eight-week self-management programme delivered by trained volunteers.[23] A recent quasi-experimental study

For peer review only



For peer review only - http://bmjopen.bmj.com/site/about/guidelines.xhtml

identified significant improvements in pain and arthritis-related self-efficacy amongst individuals with arthritis who participated in this programme.[23] The Staying Connected Programme volunteers were not required to have an arthritis diagnosis, despite peer support being recognised as a valuable approach for supporting self-management.[11, 12] The present trial's OA peer mentorship intervention therefore aimed to replicate some key elements of the Staying Connected Programme whilst also incorporating peer support. Multiple factors may explain the disparities between the findings of the present trial and the Staying Connected Programme study. In particular, the present trial was not powered to detect statistically significant differences and the Staying Connected Programme study employed a single-group pretest–post-test design. Therefore, both studies' findings should be interpreted cautiously.

This trial also presents additional limitations. Notably, 88% of participants were recruited through a physiotherapy service and hence had already received some self-management support. This could be addressed through using alternative recruitment sites, such as GP practices. Furthermore, the majority of participants were older, white, well-educated females and a Cochrane review identified that the impact of self-management education programmes for OA may vary between Caucasian, educated females and other subgroups.[41] Additionally, peer support interventions may be most effective amongst the 'hardly reached', such as individuals with lower education levels.[42] Targeting the OA peer mentorship intervention to specific subgroups could therefore be valuable. Participants and peer mentors' experiences of the OA peer mentorship intervention were explored through a nested qualitative study (reported elsewhere). However, the impact of providing the

For peer review only



For peer review only - http://bmjopen.bmj.com/site/about/guidelines.xhtml

intervention on the peer mentors was not quantitatively assessed. This is an important consideration because providing peer mentorship may have positive and/or negative effects on peer mentors.[11, 43] Another limitation of the present trial was that most participants were aware of their group allocation when completing the baseline questionnaire, which may have influenced their questionnaire responses.[44] This could be overcome in a future definitive RCT by ensuring a greater number of trained peer mentors are available and/or performing a double baseline visit.

In conclusion, this trial's findings suggest peer mentorship is a feasible, acceptable and promising approach for improving OA self-management. Further investigation of the OA peer mentorship intervention is therefore warranted. However, minor modifications to the intervention and trial procedures should be considered, particularly regarding the participant and peer mentor recruitment procedures.

## ACKNOWLEDGEMENTS

The Research team acknowledges the support of the National Institute for Health Research Clinical Research Network (NIHR CRN) and are grateful to all participants and peer mentors who were part of the study; and to the trial PPI members and expert panel group.

## FOOTNOTES



For peer review only - http://bmjopen.bmj.com/site/about/guidelines.xhtml

**Twitter:** @LeedsBRC @_Anna_Anderson

**Author contributions:** GAM contributed to the conception and design of the study; acquisition, analysis and interpretation of data; and drafting the manuscript. LM, PGC, SRK and DA contributed to the design of the study. AMA contributed to the acquisition, analysis and interpretation of data and drafting the manuscript. ECL contributed to the acquisition and interpretation of data. EDR contributed to the acquisition of data. GR contributed to the design of the study and the interpretation of data. TFM contributed to the analysis of data. All authors have been involved in revising the work for important intellectual content and have approved the final version for publication.

**Funding:** This paper presents independent research funded by the National Institute for Health Research (NIHR) under its Research for Patient Benefit (RfPB) Programme (Grant Reference Number PB-PG-1215-20012). PGC and SRK are supported in part by the NIHR Leeds Biomedical Research Centre.

**Disclaimer:** The views expressed are those of the authors and not necessarily those of the NIHR or the Department of Health and Social Care.

**Competing interests:** None declared.

**Patient consent for publication:** Not required.

For peer review only



For peer review only - http://bmjopen.bmj.com/site/about/guidelines.xhtml

**Ethics approval:** Ethical approval was gained from the Greater Manchester South Research Ethics Committee (Reference:17/NW/0238). All participants provided written informed consent prior to participating in the trial.

**Data sharing agreement:** Deidentified participant data and the trial protocol are available from the corresponding author via A.Anderson@leeds.ac.uk upon reasonable request. Reuse is permitted for the purpose of health and care research as long as the original creators are acknowledged.

For peer review only

## REFERENCES


1. James SL, Abate D, Abate KH, et al. Global, regional, and national incidence, prevalence, and years lived with disability for 354 diseases and injuries for 195 countries and territories, 1990-2017: a systematic analysis for the Global Burden of Disease Study 2017. *Lancet* 2018;392(10159):1789-858. doi: 10.1016/S0140-6736(18)32279-7
2. Turkiewicz A, Petersson IF, Bjork J, et al. Current and future impact of osteoarthritis on health care: a population-based study with projections to year 2032. *Osteoarthritis Cartilage* 2014;22(11):1826-32. doi: 10.1016/j.joca.2014.07.015
3. Hunter DJ, Schofield D, Callander E. The individual and socioeconomic impact of osteoarthritis. *Nat Rev Rheumatol.* 2014;10(7):437-41. doi: 10.1038/nrrheum.2014.44




For peer review only - http://bmjopen.bmj.com/site/about/guidelines.xhtml

4. Smith TO, Purdy R, Lister S, et al. Living with osteoarthritis: a systematic review and meta-ethnography. *Scand J Rheumatol.* 2014;43(6):441-52. doi: 10.3109/03009742.2014.894569

5. Puig-Junoy J, Ruiz Zamora A. Socio-economic costs of osteoarthritis: A systematic review of cost-of-illness studies. *Semin Arthritis Rheum* 2015;44(5):531-41. doi: https://doi.org/10.1016/j.semarthrit.2014.10.012

6. Nelson AE, Allen KD, Golightly YM, et al. A systematic review of recommendations and guidelines for the management of osteoarthritis: The chronic osteoarthritis management initiative of the U.S. bone and joint initiative. *Semin Arthritis Rheum* 2014;43(6):701-12. doi: 10.1016/j.semarthrit.2013.11.012

7. Basedow M, Esterman A. Assessing appropriateness of osteoarthritis care using quality indicators: a systematic review. *J Eval Clin Pract.* 2015;21(5):782-9. doi: 10.1111/jep.12402

8. McHugh GA, Campbell M, Luker KA. Quality of care for individuals with osteoarthritis: a longitudinal study. *J Eval Clin Pract.* 2012;18(3):534-41. doi: 10.1111/j.1365-2753.2010.01616.x

9. Darlow B, Brown M, Thompson B, et al. Living with osteoarthritis is a balancing act: an exploration of patients' beliefs about knee pain. *BMC Rheumatol.* 2018;2(1):15. doi: 10.1186/s41927-018-0023-x

10. Hunter DJ, Bowden JL. Are you managing osteoarthritis appropriately? *Nat Rev Rheumatol.* 2017;13(12):703-04. doi: 10.1038/nrrheum.2017.160

11. Embuldeniya G, Veinot P, Bell E, et al. The experience and impact of chronic disease peer support interventions: A qualitative synthesis. *Patient Educ Couns.* 2013;92(1):3-12. doi: https://doi.org/10.1016/j.pec.2013.02.002

For peer review only



For peer review only - http://bmjopen.bmj.com/site/about/guidelines.xhtml

12. Fisher EB, Boothroyd RI, Elstad EA, et al. Peer support of complex health behaviors in prevention and disease management with special reference to diabetes: systematic reviews. *Clin Diabetes Endocrinol.* 2017;3(1):4. doi: 10.1186/s40842-017-0042-3

13. Heisler M. Overview of Peer Support Models to Improve Diabetes Self-Management and Clinical Outcomes. *Diabetes Spectr* 2007;20(4):214-21. doi: 10.2337/diaspect.20.4.214

14. Hunter DJ, Bierma-Zeinstra S. Osteoarthritis. *Lancet* 2019;393(10182):1745-59. doi: 10.1016/S0140-6736(19)30417-9

15. Ali SA, Kokorelias KM, MacDermid JC, et al. Education and Social Support as Key Factors in Osteoarthritis Management Programs: A Scoping Review. *Arthritis* 2018;2018:2496190. doi: 10.1155/2018/2496190

16. Simmons D, Cohn S, Bunn C, et al. Testing a peer support intervention for people with type 2 diabetes: a pilot for a randomised controlled trial. *BMC Fam Pract.* 2013;14(1):5. doi: 10.1186/1471-2296-14-5

17. Eldridge SM, Chan CL, Campbell MJ, et al. CONSORT 2010 statement: extension to randomised pilot and feasibility trials. *BMJ* 2016;355:i5239. doi: 10.1136/bmj.i5239

18. National Institute for Health and Care Excellence. Osteoarthritis: care and management (Clinical guideline [CG177]), 2014.

19. Whitehead AL, Julious SA, Cooper CL, et al. Estimating the sample size for a pilot randomised trial to minimise the overall trial sample size for the external pilot and main trial for a continuous outcome variable. *Stat Methods Med Res* 2016;25(3):1057-73. doi: 10.1177/0962280215588241

For peer review only



For peer review only - http://bmjopen.bmj.com/site/about/guidelines.xhtml

20. McHugh GA, Campbell M, Luker KA. GP referral of patients with osteoarthritis for consideration of total joint replacement: a longitudinal study. *Br J Gen Pract.* 2011;61(589):e459-e68. doi: 10.3399/bjgp11X588420

21. Sealed Envelope Ltd. Simple randomisation service 2019. https://www.sealedenvelope.com/simple-randomiser/v1/ (accessed 16th March 2020).

22. Arthritis Research UK. Osteoarthritis. Chesterfield, UK: Arthritis Research UK 2012.

23. McHugh GA, Conaghan PG, McConville M, et al. Promoting self-management in older people with arthritis: Preliminary findings of the Northern Ireland Staying Connected Programme. *Musculoskeletal Care* 2018;16(4):489-93. doi: 10.1002/msc.1353

24. Olsson L-E, Jakobsson Ung E, Swedberg K, et al. Efficacy of person-centred care as an intervention in controlled trials – a systematic review. *J Clin Nurs.* 2013;22(3-4):456-65. doi: 10.1111/jocn.12039

25. nhs.uk. Involving people in their own care n.d. https://www.england.nhs.uk/ourwork/patient-participation/ (accessed 16th April 2020).

26. Thabane L, Ma J, Chu R, et al. A tutorial on pilot studies: the what, why and how. *BMC Med Res Methodol* 2010;10(1):1. doi: 10.1186/1471-2288-10-1

27. Smith D, Harvey P, Lawn S, et al. Measuring chronic condition self-management in an Australian community: factor structure of the revised Partners in Health (PIH) scale. *Qual Life Res.* 2017;26(1):149-59. doi: 10.1007/s11136-016-1368-5



For peer review only - http://bmjopen.bmj.com/site/about/guidelines.xhtml

28. Zimet GD, Dahlem NW, Zimet SG, et al. The Multidimensional Scale of Perceived Social Support. *J Pers Assess* 1988;52(1):30-41. doi: 10.1207/s15327752jpa5201_2

29. Bellamy N, Buchanan WW, Goldsmith CH, et al. Validation study of WOMAC: a health status instrument for measuring clinically important patient relevant outcomes to antirheumatic drug therapy in patients with osteoarthritis of the hip or knee. *J Rheumatol.* 1988;15(12):1833-40.

30. Lorig K, Chastain RL, Ung E, et al. Development and evaluation of a scale to measure perceived self-efficacy in people with arthritis. *Arthritis Rheum.* 1989;32(1):37-44. doi: 10.1002/anr.1780320107

31. Self-Management Resource Center. English Evaluation Tools: Arthritis Self-Efficacy 2020. https://www.selfmanagementresource.com/resources/evaluation-tools/english-evaluation-tools (accessed 2nd April 2020).

32. Brady TJ. Measures of self-efficacy: Arthritis Self-Efficacy Scale (ASES), Arthritis Self-Efficacy Scale-8 Item (ASES-8), Children's Arthritis Self-Efficacy Scale (CASE), Chronic Disease Self-Efficacy Scale (CDSES), Parent's Arthritis Self-Efficacy Scale (PASE), and Rheumatoid Arthritis Self-Efficacy Scale (RASE). *Arthritis Care Res (Hoboken)* 2011;63 Suppl 11:S473-85. doi: 10.1002/acr.20567

33. Zigmond AS, Snaith RP. The hospital anxiety and depression scale. *Acta Psychiatr Scand.* 1983;67(6):361-70. doi: 10.1111/j.1600-0447.1983.tb09716.x

For peer review only



For peer review only - http://bmjopen.bmj.com/site/about/guidelines.xhtml

34. Herdman M, Gudex C, Lloyd A, et al. Development and preliminary testing of the new five-level version of EQ-5D (EQ-5D-5L). *Qual Life Res* 2011;20(10):1727-36. doi: 10.1007/s11136-011-9903-x

35. Beecham J, Knapp M. Costing psychiatric interventions. In: Thornicroft G, ed. Measuring Mental Health Needs. London, United Kingdom: Gaskell 2001:200-24.

36. D'Agostino RB, Belanger A, D'Agostino RB. A Suggestion for Using Powerful and Informative Tests of Normality. *Am Stat* 1990;44(4):316-21. doi: 10.1080/00031305.1990.10475751

37. Koenker R, Hallock KF. Quantile Regression *J Econ Perspect* 2001;15(4):143-56. doi: 10.1257/jep.15.4.143

38. Williams EM, Hyer JM, Viswanathan R, et al. Peer-to-Peer Mentoring for African American Women With Lupus: A Feasibility Pilot. *Arthritis Care Res (Hoboken)* 2018;70(6):908-17. doi: 10.1002/acr.23412

39. Walshe C, Roberts D, Calman L, et al. Peer Mentors for People with Advanced Cancer: Lessons Learnt from Recruiting and Training Peer Mentors for a Feasibility Randomized Controlled Trial. *J Cancer Educ.* 2020 doi: 10.1007/s13187-020-01692-7

40. Cooper K, Jehu LM, Klein S, et al. Training peers to support older people with chronic low back pain following physiotherapy discharge: a feasibility study. *Physiotherapy* 2018;104(2):239-47. doi: https://doi.org/10.1016/j.physio.2017.07.001

41. Kroon FPB, van der Burg LRA, Buchbinder R, et al. Self-management education programmes for osteoarthritis. *Cochrane Database of Syst Rev.* 2014(1) doi: 10.1002/14651858.CD008963.pub2



For peer review only - http://bmjopen.bmj.com/site/about/guidelines.xhtml

42. Sokol R, Fisher E. Peer Support for the Hardly Reached: A Systematic Review. *Am J Public Health* 2016;106(7):e1-e8. doi: 10.2105/AJPH.2016.303180

43. Sandhu S, Veinot P, Embuldeniya G, et al. Peer-to-peer mentoring for individuals with early inflammatory arthritis: feasibility pilot. *BMJ Open* 2013;3(3):e002267. doi: 10.1136/bmjopen-2012-002267

44. Higgins JPT, Savović J, Page MJ, et al. Revised Cochrane risk-of-bias tool for randomized trials (RoB 2) 2019. https://sites.google.com/site/riskofbiastool/welcome/rob-2-0-tool/current-version-of-rob-2 (accessed 9th March 2020).

For peer review only

**FIGURE LEGEND**

**Figure 1: Participant flow diagram**

[a] Minimum number of individuals approached/excluded. Additional individuals may have been approached/excluded because the number of individuals approached through physiotherapy clinics was not recorded to minimise the administrative burden on physiotherapists.

[b] Intervention completion was defined as completion of at least three peer mentorship sessions.



For peer review only - http://bmjopen.bmj.com/site/about/guidelines.xhtml

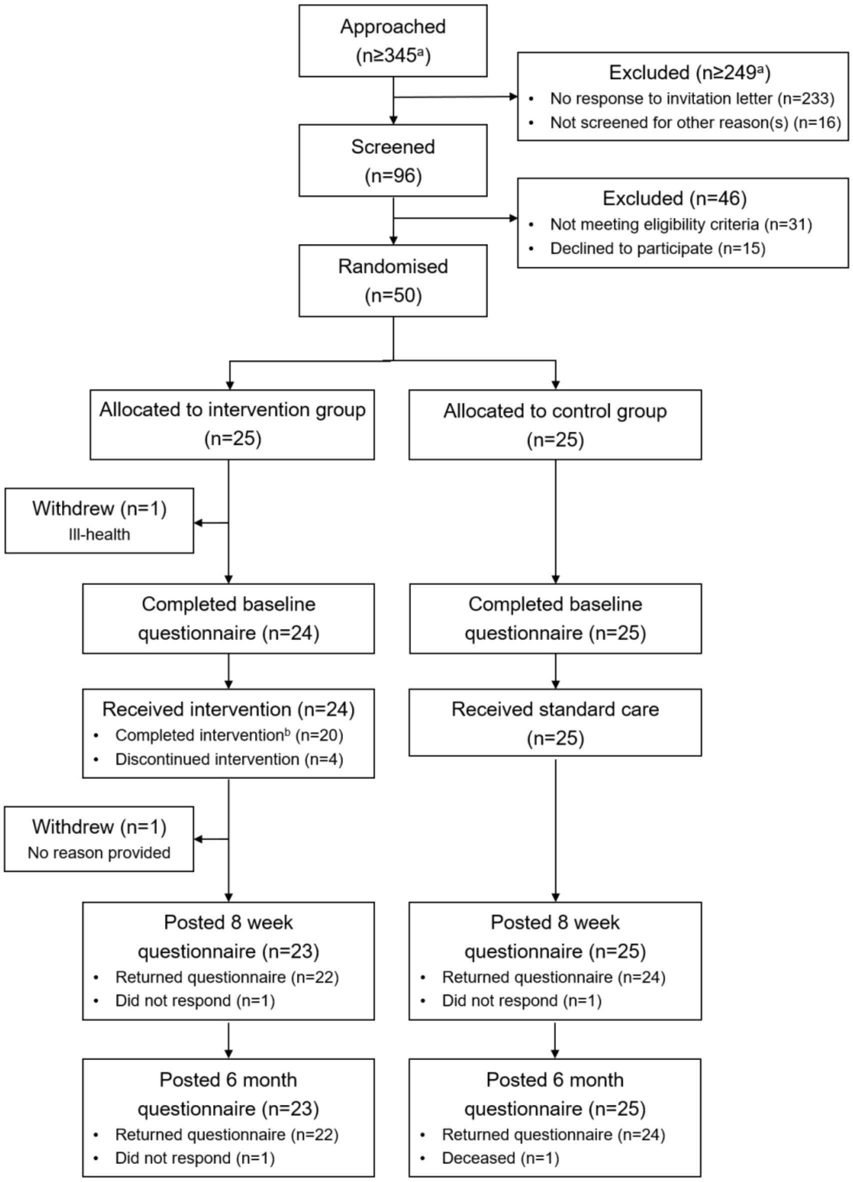


[a] Minimum number of individuals approached/excluded. Additional individuals may have been approached/excluded because the number of individuals approached through physiotherapy clinics was not recorded to minimise the administrative burden on physiotherapists.
[b] Intervention completion was defined as completion of at least three peer mentorship sessions.

214x295mm (150 x 150 DPI)

# Peer mentorship to improve self-management of osteoarthritis: A randomised feasibility trial – Supplementary Material

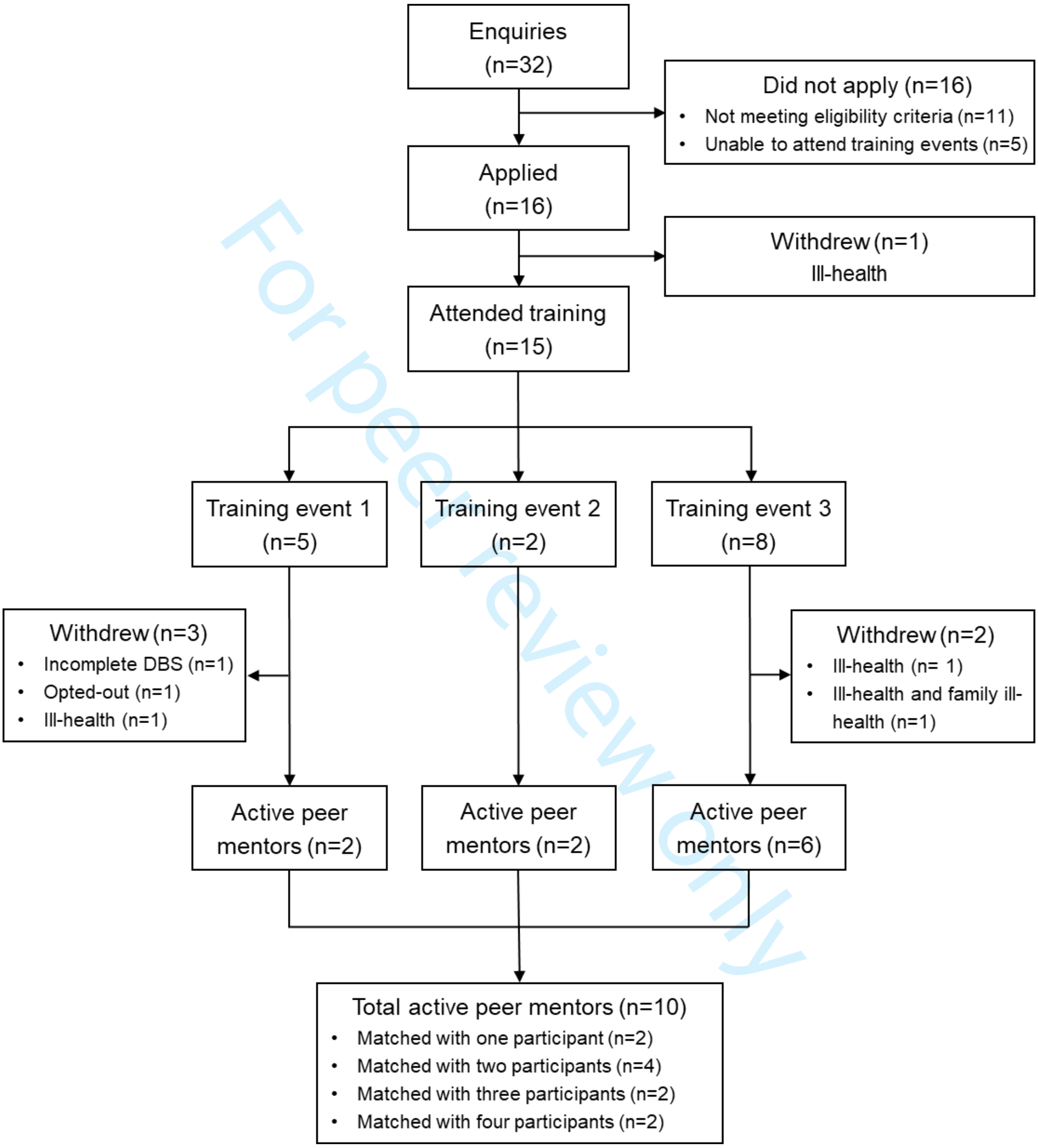


**Supplementary Figure 1: Peer mentor flow diagram**

DBS, Enhanced Disclosure and Barring Service check



For peer review only - http://bmjopen.bmj.com/site/about/guidelines.xhtml

Supplementary Material

## Supplementary Table 1: CONSORT 2010 checklist of information to include when reporting a pilot or feasibility randomised trial in a journal or conference abstract

| Item | Description | Reported on line number |
|---|---|---|
| Title | Identification of study as randomised pilot or feasibility trial | 2 |
| Authors | Contact details for the corresponding author | 15-17 |
| Trial design | Description of pilot trial design (eg, parallel, cluster) | 28 |
| **Methods** | | |
| Participants | Eligibility criteria for participants and the settings where the pilot trial was conducted | 29-30, 33 |
| Interventions | Interventions intended for each group | 32-35 |
| Objective | Specific objectives of the pilot trial | 25-27 |
| Outcome | Prespecified assessment or measurement to address the pilot trial objectives | 36-40 |
| Randomisation | How participants were allocated to interventions | 32 |
| Blinding (masking) | Whether or not participants, care givers, and those assessing the outcomes were blinded to group assignment | 28 |
| **Results** | | |
| Numbers randomised | Number of participants screened and randomised to each group for the pilot trial objectives | 41-42 |
| Recruitment | Trial status | N/A[1] |
| Numbers analysed | Number of participants analysed in each group for the pilot objectives | 42-45 |
| Outcome | Results for the pilot objectives, including any expressions of uncertainty | 41-48 |
| Harms | Important adverse events or side effects | 45-46 |
| Conclusions | General interpretation of the results of pilot trial and their implications for the future definitive trial | 49-52 |
| Trial registration | Registration number for pilot trial and name of trial register | 53 |
| Funding | Source of funding for pilot trial | 54 |

For peer review only

N/A, Not applicable

[1] Not applicable as item is only applicable to conference abstracts.

Checklist from: Eldridge SM, Chan CL, Campbell MJ, et al. CONSORT 2010 statement: extension to randomised pilot and feasibility trials. *BMJ* 2016;355:i5239.

For peer review only

Supplementary Material

## Supplementary Table 2: CONSORT 2010 checklist of information to include when reporting a pilot or feasibility trial

| Section/Topic | Item No | Checklist item | Reported on page No |
|---|---|---|---|
| **Title and abstract** | | | |
| | 1a | Identification as a pilot or feasibility randomised trial in the title | 1 |
| | 1b | Structured summary of pilot trial design, methods, results, and conclusions | 2-3 |
| **Introduction** | | | |
| Background and objectives | 2a | Scientific background and explanation of rationale for future definitive trial, and reasons for randomised pilot trial | 4-5 |
| | 2b | Specific objectives or research questions for pilot trial | 5 |
| **Methods** | | | |
| Trial design | 3a | Description of pilot trial design (such as parallel, factorial) including allocation ratio | 5 |
| | 3b | Important changes to methods after pilot trial commencement (such as eligibility criteria), with reasons | 10 |
| Participants | 4a | Eligibility criteria for participants | 6 |
| | 4b | Settings and locations where the data were collected | 6, 10, 13 |
| | 4c | How participants were identified and consented | 6 |
| Interventions | 5 | The interventions for each group with sufficient details to allow replication, including how and when they were actually administered | 7-11 |
| Outcomes | 6a | Completely defined prespecified assessments or measurements to address each pilot trial objective specified in 2b, including how and when they were assessed | 11-15 |



For peer review only - http://bmjopen.bmj.com/site/about/guidelines.xhtml

Supplementary Material

| | | | |
|---|---|---|---|
| | 6b | Any changes to pilot trial assessments or measurements after the pilot trial commenced, with reasons | N/A |
| | 6c | If applicable, prespecified criteria used to judge whether, or how, to proceed with future definitive trial | 12 |
| Sample size | 7a | Rationale for numbers in the pilot trial | 7 |
| | 7b | When applicable, explanation of any interim analyses and stopping guidelines | N/A |
| Randomisation: | | | |
| Sequence generation | 8a | Method used to generate the random allocation sequence | 7 |
| | 8b | Type of randomisation(s); details of any restriction (such as blocking and block size) | 7 |
| Allocation concealment mechanism | 9 | Mechanism used to implement the random allocation sequence (such as sequentially numbered containers), describing any steps taken to conceal the sequence until interventions were assigned | 7 |
| Implementation | 10 | Who generated the random allocation sequence, who enrolled participants, and who assigned participants to interventions | 6-7 |
| Blinding | 11a | If done, who was blinded after assignment to interventions (for example, participants, care providers, those assessing outcomes) and how | N/A |
| | 11b | If relevant, description of the similarity of interventions | N/A |
| Statistical methods | 12 | Methods used to address each pilot trial objective whether qualitative or quantitative | 12, 15 |
| **Results** | | | |
| Participant flow (a diagram is strongly recommended) | 13a | For each group, the numbers of participants who were approached and/or assessed for eligibility, randomly assigned, received intended treatment, and were assessed for each objective | 17-26<br>Figure 1 |
| | 13b | For each group, losses and exclusions after randomisation, together with reasons | 17-18 |



For peer review only - http://bmjopen.bmj.com/site/about/guidelines.xhtml

Supplementary Material

| | | | |
|---|---|---|---|
| | | | Figure 1 |
| Recruitment | 14a | Dates defining the periods of recruitment and follow-up | 5, 17 |
| | 14b | Why the pilot trial ended or was stopped | N/A |
| Baseline data | 15 | A table showing baseline demographic and clinical characteristics for each group | 20-21<br>Supplementary table 6 |
| Numbers analysed | 16 | For each objective, number of participants (denominator) included in each analysis. If relevant, these numbers should be by randomised group | 17-26 |
| Outcomes and estimation | 17 | For each objective, results including expressions of uncertainty (such as 95% confidence interval) for any estimates. If relevant, these results should be by randomised group | 17-26 |
| Ancillary analyses | 18 | Results of any other analyses performed that could be used to inform the future definitive trial | N/A |
| Harms | 19 | All important harms or unintended effects in each group | N/A |
| | 19a | If relevant, other important unintended consequences | N/A |
| **Discussion** | | | |
| Limitations | 20 | Pilot trial limitations, addressing sources of potential bias and remaining uncertainty about feasibility | 279-30 |
| Generalisability | 21 | Generalisability (applicability) of pilot trial methods and findings to future definitive trial and other studies | 29 |
| Interpretation | 22 | Interpretation consistent with pilot trial objectives and findings, balancing potential benefits and harms, and considering other relevant evidence | 26-30 |
| | 22a | Implications for progression from pilot to future definitive trial, including any proposed amendments | 26-30 |

For peer review only



For peer review only - http://bmjopen.bmj.com/site/about/guidelines.xhtml

| **Other information** | | | |
|---|---|---|---|
| Registration | 23 | Registration number for pilot trial and name of trial registry | 5 |
| Protocol | 24 | Where the pilot trial protocol can be accessed, if available | 32 |
| Funding | 25 | Sources of funding and other support (such as supply of drugs), role of funders | 31 |
| | 26 | Ethical approval or approval by research review committee, confirmed with reference number | 5, 32 |

Checklist from: Eldridge SM, Chan CL, Campbell MJ, et al. CONSORT 2010 statement: extension to randomised pilot and feasibility trials. *BMJ* 2016;355:i5239.

For peer review only

**Supplementary Table 3: Overview of areas covered during the peer mentor training event**

| Day 1 | Day 2 |
|---|---|
| • Overview of the trial and peer mentor role<br>• Osteoarthritis key facts and self-management topics<br>• Goal setting<br>• Pacing<br>• Educational resource pack review<br>• Exercise<br>• Pain management<br>• Sleep | • Review of day 1<br>• Communication<br>• Professional boundaries<br>• Mentoring in practice (including safeguarding, disclosures and confidentiality)<br>• Visit and recording procedures<br>• Trial data collection<br>• Next steps |

For peer review only



For peer review only - http://bmjopen.bmj.com/site/about/guidelines.xhtml

**Supplementary Table 4: Estimated costs of delivering the two day peer mentor training event to five peer mentors**

| **Staff costs**[a] | | | |
|---|---|---|---|
| | Hourly rate[b] | Hours per event | Cost per event |
| Research Fellow (Grade 7) | £32.23 | 7.5 | £241.73 |
| Research Associate (Grade 6) | £26.09 | 15 | £391.35 |
| Volunteer Coordinator (Grade 5) | £21.79 | 15 | £326.85 |
| Total staff costs | | | £959.93 |
| **Peer mentor costs** | | | |
| | Cost per peer mentor | | Cost per event |
| Refreshments | £18.00 | | £90.00 |
| Notebook | £2.48 | | £12.40 |
| Ring binder | £2.00 | | £10.00 |
| Plastic wallets | £0.67 | | £3.33 |
| Printing | £10.50 | | £52.50 |
| Name badge | £0.14 | | £0.68 |
| Travel expenses | £13.14 | | £65.70 |
| Total peer mentor costs | £46.92 | | £234.62 |
| **Overall costs** | | | |
| | Cost per peer mentor | | Cost per event |
| Overall costs | £238.91 | | £1,194.54 |

[a] Staff costs were based on the events run by the trial team members.

[b] Includes staff salary, National Insurance contributions and pension contributions, but does not include estates costs or indirect costs.

For peer review only

**Supplementary Table 5: Estimated costs of delivering the OA peer mentorship intervention to one participant**

| | | |
|---|---|---|
| **Volunteer Coordinator costs (Grade 5 hourly rate £21.79[a])** | | |
| | Per single session | Per 5.79 sessions[b] |
| Hours spent supporting mentorship sessions (0.5 hours per session) | £10.90 | £63.10 |
| Additional hours spent on matching, initial session and closure (4 hours) | £15.05 | £87.16 |
| Travel expenses for initial session | £0.81 | £4.72 |
| Total Volunteer Coordinator costs | £26.76 | £154.98 |
| **Peer mentor costs** | | |
| | Per single session | Per 5.79 sessions[b] |
| Honorarium | £15 | £86.88 |
| Travel expenses | £5.53 | £32.01 |
| Total peer mentor costs | £20.53 | £118.89 |
| **Overall costs** | | |
| | Per single session | Per 5.79 sessions[b] |
| Overall costs | £47.29 | £273.86 |

[a] Includes staff salary, National Insurance contributions and pension contributions, but does not include estates costs or indirect costs.

[b] 5.79 was the mean number of mentorship sessions received by participants.

For peer review only

## Supplementary Table 6: Additional participant baseline characteristics

| Characteristics | Intervention (n=24) | Control (n=25) |
|---|---|---|
| **Marital status,** n (%) | | |
| Married | 12 (50.0) | 17 (68.0) |
| Divorced | 6 (25.0) | 2 (8.0) |
| Living together | 0 (0.0) | 1 (4.0) |
| Widowed | 5 (20.8) | 3 (12.0) |
| Single | 1 (4.2) | 2 (8.0) |
| **Current smokers,** n (%) | 1 (4.2) | 2 (8.0) |
| **Areas affected by arthritis**, n (%) | | |
| Single hip | 0 (0.0) | 0 (0.0) |
| Both hips | 2 (8.3) | 4 (16.0) |
| Single knee | 2 (8.3) | 3 (12.0) |
| Both knees | 8 (33.3) | 9 (36.0) |
| Single hip and single knee | 2 (8.3) | 3 (12.0) |
| Both hips and one knee | 2 (8.3) | 1 (4.0) |
| Single hip and both knees | 5 (20.8) | 2 (8.0) |
| Both hips and both knees | 3 (12.5) | 3 (12.0) |
| **Areas affected by joint pain for > 6 weeks in the last 3 months**, n (%) | | |
| Neck | 7 (29.2) | 6 (24.0) |
| Shoulder (right) | 7 (29.2) | 6 (24.0) |
| Shoulder (left) | 7 (29.2) | 7 (28.0) |
| Elbow (right) | 0 (0.0) | 2 (8.0) |
| Elbow (left) | 0 (0.0) | 2 (8.0) |
| Hand (right) | 9 (37.5) | 6 (24.0) |
| Hand (left) | 9 (37.5) | 5 (20.0) |
| Back | 6 (25.0) | 11 (44.0) |
| Hip (right) | 10 (41.7) | 10 (40.0) |
| Hip (left) | 10 (41.7) | 10 (40.0) |
| Knee (right) | 19 (79.2) | 16 (64.0) |
| Knee (left) | 19 (79.2) | 20 (80.0) |



For peer review only - http://bmjopen.bmj.com/site/about/guidelines.xhtml

For peer review only

| | | |
|---|---|---|
| Foot (right) | 5 (20.8) | 4 (16.0) |
| Foot (left) | 4 (16.7) | 5 (16.0) |
| **Duration of pain in years,** mean (SD) | 4.7 (5.2) | 5.3 (6.2) |
| **Currently suffering from pain**, n (%) | 23 (95.8) | 25 (100.0) |
| **Frequency of pain**[a], n (%) | | |
| Always | 7 (30.4) | 11 (44.0) |
| Often present | 5 (21.7) | 9 (36.0) |
| Sometimes present | 10 (43.5) | 3 (20.0) |
| Present very little | 1 (4.4) | 0 (0.0) |
| **Previous hip replacement,** n (%) | 1 (4.2) | 1 (4) |
| **Previous knee replacement(s),** n (%) | 1 (4.2) | 2 (8) |
| **Walking aid use,** n (%) | 8 (33.3) | 7 (28.0) |
| **Currently using medication for pain or other symptoms of arthritis,** n (%) | 20 (83.3) | 21 (84.0) |
| **Currently using strong opioids,** n, (%) | 1 (4.2) | 0 (0.0) |
| **Currently using over-the-counter NSAIDS,** n (%) | 4 (16.7) | 6 (24.0) |

NSAIDS, non-steroidal anti-inflammatory drugs; SD, standard deviation.

[a] Frequency of pain intervention group n=23 because one participant reported they were not currently suffering from pain.



For peer review only - http://bmjopen.bmj.com/site/about/guidelines.xhtml

Supplementary Material

**Supplementary Table 7: Mean resource use and mean difference between groups for the 8 week and 6 month questionnaires combined**

| | Intervention group mean use (n=22) | Control group mean use (n=24) | Mean difference (95% Confidence Interval) |
|---|---|---|---|
| Inpatient length of stay (nights) | 0.05 | 0.96 | -0.91 (-1.78 to -0.04) |
| Number of Outpatient appointments | 3.05 | 2.92 | 0.13 (-1.87 to 2.13) |
| Number GP appointments (surgery) | 2.73 | 2.42 | 0.31 (-1.47 to 2.09) |
| Number of GP visits (home) | 0.00 | 0.17 | -0.17 (-0.52 to 0.18) |
| Number of GP calls (telephone) | 0.59 | 0.67 | -0.08 (-0.70 to 0.55) |
| Number of GP Practice Nurse visits | 1.64 | 0.71 | 0.93 (0.15 to 1.71) |
| Number of District Nurse visits | 0.00 | 0.00 | 0.00 |
| Number of Occupational Therapist visits | 0.09 | 0.21 | -0.12 (-0.47 to 0.24) |
| Number of Physiotherapist visits | 1.36 | 1.96 | -0.59 (-2.74 to 1.55) |
| Number of Chiropodist visits | 0.64 | 0.75 | -0.11 (-0.86 to 0.63) |
| Number of Pharmacist visits | 2.27 | 1.67 | 0.61 (-1.04 to 2.25) |
| Number of NHS Direct calls | 0.18 | 0.13 | 0.06 (-0.28 to 0.39) |
| Number of Walk-in Centre visits | 0.41 | 0.13 | 0.28 (-0.43 to 1.00) |
| Number of Counsellor visits | 0.09 | 0.08 | 0.01 (-0.24 to 0.26) |

For peer review only



For peer review only - http://bmjopen.bmj.com/site/about/guidelines.xhtml

CONSORT checklists

# Peer mentorship to improve self-management of osteoarthritis: A randomised feasibility trial – CONSORT checklists

## CONSORT 2010 checklist of information to include when reporting a pilot or feasibility randomised trial in a journal or conference abstract

| Item | Description | Reported on line number |
|---|---|---|
| Title | Identification of study as randomised pilot or feasibility trial | 2 |
| Authors | Contact details for the corresponding author | 15-17 |
| Trial design | Description of pilot trial design (eg, parallel, cluster) | 28 |
| **Methods** | | |
| Participants | Eligibility criteria for participants and the settings where the pilot trial was conducted | 29-30, 33 |
| Interventions | Interventions intended for each group | 32-35 |
| Objective | Specific objectives of the pilot trial | 25-27 |
| Outcome | Prespecified assessment or measurement to address the pilot trial objectives | 36-40 |
| Randomisation | How participants were allocated to interventions | 32 |
| Blinding (masking) | Whether or not participants, care givers, and those assessing the outcomes were blinded to group assignment | 28 |
| **Results** | | |
| Numbers randomised | Number of participants screened and randomised to each group for the pilot trial objectives | 41-42 |
| Recruitment | Trial status | N/A[1] |
| Numbers analysed | Number of participants analysed in each group for the pilot objectives | 42-45 |
| Outcome | Results for the pilot objectives, including any expressions of uncertainty | 41-48 |
| Harms | Important adverse events or side effects | 45-46 |
| Conclusions | General interpretation of the results of pilot trial and their implications for the future definitive trial | 49-52 |
| Trial registration | Registration number for pilot trial and name of trial register | 53 |



For peer review only - http://bmjopen.bmj.com/site/about/guidelines.xhtml

CONSORT checklists

| | | |
|---|---|---|
| Funding | Source of funding for pilot trial | 54 |

N/A, Not applicable

[1] Not applicable as item is only applicable to conference abstracts.

Checklist from: Eldridge SM, Chan CL, Campbell MJ, et al. CONSORT 2010 statement: extension to randomised pilot and feasibility trials. *BMJ* 2016;355:i5239.

For peer review only



For peer review only - http://bmjopen.bmj.com/site/about/guidelines.xhtml

CONSORT checklists

**CONSORT 2010 checklist of information to include when reporting a pilot or feasibility trial**

| Section/Topic | Item No | Checklist item | Reported on page No |
|---|---|---|---|
| **Title and abstract** | | | |
| | 1a | Identification as a pilot or feasibility randomised trial in the title | 1 |
| | 1b | Structured summary of pilot trial design, methods, results, and conclusions | 2-3 |
| **Introduction** | | | |
| Background and objectives | 2a | Scientific background and explanation of rationale for future definitive trial, and reasons for randomised pilot trial | 4-5 |
| | 2b | Specific objectives or research questions for pilot trial | 5 |
| **Methods** | | | |
| Trial design | 3a | Description of pilot trial design (such as parallel, factorial) including allocation ratio | 5 |
| | 3b | Important changes to methods after pilot trial commencement (such as eligibility criteria), with reasons | 10 |
| Participants | 4a | Eligibility criteria for participants | 6 |
| | 4b | Settings and locations where the data were collected | 6, 10, 13 |
| | 4c | How participants were identified and consented | 6 |
| Interventions | 5 | The interventions for each group with sufficient details to allow replication, including how and when they were actually administered | 7-11 |
| Outcomes | 6a | Completely defined prespecified assessments or measurements to address each pilot trial objective specified in 2b, including how and when they were assessed | 11-15 |



For peer review only - http://bmjopen.bmj.com/site/about/guidelines.xhtml

CONSORT checklists

| | | | |
|---|---|---|---|
| | 6b | Any changes to pilot trial assessments or measurements after the pilot trial commenced, with reasons | N/A |
| | 6c | If applicable, prespecified criteria used to judge whether, or how, to proceed with future definitive trial | 12 |
| Sample size | 7a | Rationale for numbers in the pilot trial | 7 |
| | 7b | When applicable, explanation of any interim analyses and stopping guidelines | N/A |
| Randomisation: | | | |
| Sequence generation | 8a | Method used to generate the random allocation sequence | 7 |
| | 8b | Type of randomisation(s); details of any restriction (such as blocking and block size) | 7 |
| Allocation concealment mechanism | 9 | Mechanism used to implement the random allocation sequence (such as sequentially numbered containers), describing any steps taken to conceal the sequence until interventions were assigned | 7 |
| Implementation | 10 | Who generated the random allocation sequence, who enrolled participants, and who assigned participants to interventions | 6-7 |
| Blinding | 11a | If done, who was blinded after assignment to interventions (for example, participants, care providers, those assessing outcomes) and how | N/A |
| | 11b | If relevant, description of the similarity of interventions | N/A |
| Statistical methods | 12 | Methods used to address each pilot trial objective whether qualitative or quantitative | 12, 15 |
| **Results** | | | |
| Participant flow (a diagram is strongly recommended) | 13a | For each group, the numbers of participants who were approached and/or assessed for eligibility, randomly assigned, received intended treatment, and were assessed for each objective | 17-26 Figure 1 |
| | 13b | For each group, losses and exclusions after randomisation, together with reasons | 17-18 |

For peer review only

CONSORT checklists

| | | | |
|---|---|---|---|
| | | | Figure 1 |
| Recruitment | 14a | Dates defining the periods of recruitment and follow-up | 5, 17 |
| | 14b | Why the pilot trial ended or was stopped | N/A |
| Baseline data | 15 | A table showing baseline demographic and clinical characteristics for each group | 20-21<br>Supplementary table 6 |
| Numbers analysed | 16 | For each objective, number of participants (denominator) included in each analysis. If relevant, these numbers should be by randomised group | 17-26 |
| Outcomes and estimation | 17 | For each objective, results including expressions of uncertainty (such as 95% confidence interval) for any estimates. If relevant, these results should be by randomised group | 17-26 |
| Ancillary analyses | 18 | Results of any other analyses performed that could be used to inform the future definitive trial | N/A |
| Harms | 19 | All important harms or unintended effects in each group | N/A |
| | 19a | If relevant, other important unintended consequences | N/A |
| **Discussion** | | | |
| Limitations | 20 | Pilot trial limitations, addressing sources of potential bias and remaining uncertainty about feasibility | 279-30 |
| Generalisability | 21 | Generalisability (applicability) of pilot trial methods and findings to future definitive trial and other studies | 29 |
| Interpretation | 22 | Interpretation consistent with pilot trial objectives and findings, balancing potential benefits and harms, and considering other relevant evidence | 26-30 |
| | 22a | Implications for progression from pilot to future definitive trial, including any proposed amendments | 26-30 |



For peer review only - http://bmjopen.bmj.com/site/about/guidelines.xhtml

For peer review only

CONSORT checklists

| **Other information** | | | |
|---|---|---|---|
| Registration | 23 | Registration number for pilot trial and name of trial registry | 5 |
| Protocol | 24 | Where the pilot trial protocol can be accessed, if available | 32 |
| Funding | 25 | Sources of funding and other support (such as supply of drugs), role of funders | 31 |
| | 26 | Ethical approval or approval by research review committee, confirmed with reference number | 5, 32 |

Checklist from: Eldridge SM, Chan CL, Campbell MJ, et al. CONSORT 2010 statement: extension to randomised pilot and feasibility trials. *BMJ* 2016;355:i5239.

For peer review only

BMJ Open

# Peer mentorship to improve self-management of hip and knee osteoarthritis: A randomised feasibility trial

| | |
|---|---|
| Journal: | *BMJ Open* |
| Manuscript ID | bmjopen-2020-045389.R1 |
| Article Type: | Original research |
| Date Submitted by the Author: | 10-Mar-2021 |
| Complete List of Authors: | Anderson, Anna; University of Leeds, School of Healthcare; University of Leeds, Leeds Institute of Rheumatic and Musculoskeletal Medicine and NIHR Leeds Biomedical Research Centre<br>Lavender, Elizabeth; University of Leeds, School of Healthcare<br>Dusabe-Richards, Esther; University of Leeds, School of Healthcare<br>Mebrahtu, Teumzghi; University of Leeds, School of Healthcare<br>McGowan, Linda; University of Leeds, School of Healthcare<br>Conaghan, Philip; University of Leeds, Leeds Institute of Rheumatic & Musculoskeletal Medicine and NIHR Leeds Biomedical Research Centre<br>Kingsbury, Sarah; University of Leeds, Leeds Institute of Rheumatic & Musculoskeletal Medicine and NIHR Leeds Biomedical Research Centre<br>Richardson, Gerry; University of York, Centre for Health Economics; NIHR Research Design Service for Yorkshire and the Humber<br>Antcliff, Deborah; University of Leeds, School of Healthcare; Northern Care Alliance NHS Group, Physiotherapy Department, Bury Care Organisation<br>McHugh, Gretl; University of Leeds, School of Healthcare |
| <b>Primary Subject Heading</b>: | Rheumatology |
| Secondary Subject Heading: | Patient-centred medicine |
| Keywords: | RHEUMATOLOGY, PAIN MANAGEMENT, SOCIAL MEDICINE |

SCHOLARONE™
Manuscripts

*I, the Submitting Author has the right to grant and does grant on behalf of all authors of the Work (as defined in the below author licence), an exclusive licence and/or a non-exclusive licence for contributions from authors who are: i) UK Crown employees; ii) where BMJ has agreed a CC-BY licence shall apply, and/or iii) in accordance with the terms applicable for US Federal Government officers or employees acting as part of their official duties; on a worldwide, perpetual, irrevocable, royalty-free basis to BMJ Publishing Group Ltd ("BMJ") its licensees and where the relevant Journal is co-owned by BMJ to the co-owners of the Journal, to publish the Work in this journal and any other BMJ products and to exploit all rights, as set out in our licence.*

*The Submitting Author accepts and understands that any supply made under these terms is made by BMJ to the Submitting Author unless you are acting as an employee on behalf of your employer or a postgraduate student of an affiliated institution which is paying any applicable article publishing charge ("APC") for Open Access articles. Where the Submitting Author wishes to make the Work available on an Open Access basis (and intends to pay the relevant APC), the terms of reuse of such Open Access shall be governed by a Creative Commons licence – details of these licences and which Creative Commons licence will apply to this Work are set out in our licence referred to above.*

*Other than as permitted in any relevant BMJ Author's Self Archiving Policies, I confirm this Work has not been accepted for publication elsewhere, is not being considered for publication elsewhere and does not duplicate material already published. I confirm all authors consent to publication of this Work and authorise the granting of this licence.*

For peer review only

# Peer mentorship to improve self-management of hip and knee osteoarthritis: A randomised feasibility trial

Anna M. Anderson[ab*], Elizabeth C. Lavender[a], Esther Dusabe-Richards[a], Teumzghi F. Mebrahtu[a], Linda McGowan[a], Philip G. Conaghan[b], Sarah R. Kingsbury[b], Gerry Richardson[c], Deborah Antcliff[ad], Gretl A. McHugh[a]

[a] School of Healthcare, University of Leeds, Leeds, United Kingdom

[b] Leeds Institute of Rheumatic & Musculoskeletal Medicine and NIHR Leeds Biomedical Research Centre, University of Leeds, Leeds, United Kingdom

[c] Centre for Health Economics, University of York, York, United Kingdom, and The NIHR Research Design Service for Yorkshire & the Humber, York, United Kingdom

[d] Physiotherapy Department, Bury Care Organisation, Northern Care Alliance NHS Group, Bury, United Kingdom

*Corresponding author at: Leeds Institute of Rheumatic & Musculoskeletal Medicine, University of Leeds, Leeds, United Kingdom,

Email address: A.Anderson@leeds.ac.uk

Word count: 5,232

Keywords: Rheumatology; Pain management; Social medicine



For peer review only - http://bmjopen.bmj.com/site/about/guidelines.xhtml

## ABSTRACT

**Objective** To determine the feasibility of conducting a randomised controlled trial (RCT) of a peer mentorship intervention to improve self-management of osteoarthritis (OA).

**Design** Six-month parallel-group non-blinded randomised feasibility trial.

**Setting** One secondary care and one primary care United Kingdom NHS Trust.

**Participants** Fifty adults aged ≥55 years old with hip and/or knee OA.

**Interventions** Participants were allocated 1:1 to the intervention or control group using an online randomisation service. Intervention group participants received usual care (information resources) and up to eight community-based self-management support sessions delivered by a peer mentor (trained volunteer with hip and/or knee OA). Control group participants received usual care only.

**Outcome measures** Key feasibility outcomes were participant and peer mentor recruitment and attrition, intervention completion and the sample size required for a definitive RCT. Based on these feasibility outcomes, four success criteria for proceeding to a definitive RCT were pre-specified. Patient-reported outcomes were collected via questionnaires at baseline, eight weeks and six months.

**Results** Ninety-six individuals were screened, 65 were eligible and 50 were randomised (25 per group). Of the 24 participants who commenced the intervention, 20 completed it. Four participants did not complete the six-month questionnaire. Twenty-one individuals were eligible for the peer mentor role, 15 were trained and five withdrew prior to being matched with a participant. No intervention-related harms occurred. Allowing for 20% attrition, the sample size required for a definitive RCT




For peer review only - http://bmjopen.bmj.com/site/about/guidelines.xhtml

was calculated as 170 participants. The intervention group showed improvements in self-management compared to the control group.

**Conclusions** The feasibility outcomes achieved the pre-specified criteria for proceeding to an RCT. The exploratory analyses suggest peer mentorship may improve OA self-management. An RCT of the OA peer mentorship intervention is therefore warranted with minor modifications to the intervention and trial procedures.

**Trial registration** ISRCTN:50675542

**Funding** National Institute for Health Research (NIHR)

For peer review only

## STRENGTHS AND LIMITATIONS OF THIS STUDY

- This randomised feasibility trial is the first study to develop a novel peer mentorship intervention specifically focused on improving self-management of hip and knee osteoarthritis (OA).
- Extensive Patient and Public Involvement ensured the intervention and trial procedures were tailored to the needs of individuals with OA.
- A comprehensive range of feasibility outcomes were assessed, providing valuable information for designing a future definitive randomised controlled trial.
- Validated patient-reported outcome measures were administered; however, the results must be interpreted cautiously because the trial was not powered to detect statistically significant differences.
- A key limitation was that the majority of participants were recruited through a physiotherapy service and hence had already received some self-management support.



For peer review only - http://bmjopen.bmj.com/site/about/guidelines.xhtml

## INTRODUCTION

Osteoarthritis (OA) is one of the most prevalent musculoskeletal conditions, with hip and knee OA affecting over 300 million individuals worldwide.[1] Furthermore, the prevalence of OA is increasing due to the ageing population and rising obesity levels.[2] Individuals with OA often experience severe pain, impaired function and reduced quality of life.[3] Osteoarthritis can have a profound psychosocial impact,[4] and results in substantial economic burden.[5]

National and international guidelines emphasise that patient education, self-management strategies and exercise are core elements for managing OA.[6] However, implementation of OA guidelines is currently poor.[7] Patients report receiving insufficient information about OA management,[8] and having a limited understanding of the condition.[4, 9] This negatively impacts patients' health behaviours.[4, 9] A need for tailored interventions to support OA self-management has therefore been highlighted.[10]

Peer support interventions are an established approach for supporting chronic condition self-management.[11, 12] Various peer support models have been described, such as peer-led group programmes and peer mentorship.[13] The latter involves a trained individual with a particular health condition (the 'peer mentor') providing one-to-one support to another individual with the same condition.[13] This approach is likely to be particularly valuable for individuals with OA due to the heterogeneous nature of the condition, which means tailored support is crucial.[10, 14]



For peer review only - http://bmjopen.bmj.com/site/about/guidelines.xhtml

Previous studies have highlighted the value of OA interventions incorporating peer support.[15] However, no previous studies have explored an OA peer mentorship intervention. Although such an intervention offers multiple potential benefits, peer mentorship interventions can present feasibility/acceptability issues, such as high peer mentor attrition.[16] Therefore, this feasibility trial aimed to develop and trial a peer mentorship intervention to improve OA self-management. Its key objectives were to determine the feasibility of conducting a randomised controlled trial (RCT) of the OA peer mentorship intervention in terms of: the feasibility/acceptability of the intervention and trial procedures; participant recruitment and retention; questionnaire completion rates; generating the sample size required for a definitive RCT; and estimating the intervention costs. Additionally, the potential impact of the intervention on patient-reported outcomes and resource use was explored.

**METHODS**

This was a six-month parallel-group randomised feasibility trial. A nested qualitative study was included (reported elsewhere). The trial was prospectively registered (ISRCTN:50675542) and conducted between 1st September 2017 and 16th February 2020. Ethical approval was gained from the Greater Manchester South Research Ethics Committee (Reference 17/NW/0238). The trial is reported according to the CONSORT 2010 extension for randomised pilot and feasibility trials,[17] (online supplementary tables 1 and 2). The development and feasibility testing of the OA peer mentorship intervention was guided by the Medical Research Council guidance on developing and evaluating complex interventions.[18]



For peer review only - http://bmjopen.bmj.com/site/about/guidelines.xhtml

**Trial procedures and participants**

Recruitment and consent

Potential participants were identified from rheumatology and orthopaedic clinics of one secondary care National Health Service (NHS) Trust and physiotherapy clinics and electronic records of one primary care NHS Trust. Both are large Trusts in Northern England. The initial approach was made via clinical staff during clinic appointments or through an invitation letter. Individuals interested in participating were provided with further details about the trial and screened for eligibility by a researcher at their clinic appointment or via telephone.

All participants provided written informed consent at their clinic appointment or during a baseline visit from a researcher. The latter took place in the participant's home or another private location of the participant's choice.

Eligibility criteria

Inclusion criteria were: 1) aged ≥55 years old, and 2) clinician-confirmed diagnosis of hip and/or knee OA.[19] Exclusion criteria were: 1) presence of inflammatory arthritis (including gout and rheumatoid arthritis), 2) serious health conditions that would prevent participation, and 3) listed for hip/knee replacement.

For peer review only



For peer review only - http://bmjopen.bmj.com/site/about/guidelines.xhtml

## Sample size

Recommendations for pilot studies suggest 20 participants per arm is acceptable assuming at worst a small effect size (Cohen's d=0.2) for a continuous outcome and 80% power.[20] Allowing for 20% attrition,[21] the sample size was set at 25 participants per arm.

## Randomisation

Participants were randomised to the intervention or control group with 1:1 allocation using the Sealed Envelope online randomisation service.[22] This was set up by an independent statistician to generate blocked randomisation with varying block lengths, stratified according to educational level. The researcher performing the randomisation was unable to access the allocation codes. Given the nature of the intervention, participant blinding was not possible.

## Intervention group

Intervention group participants received usual care and the OA peer mentorship intervention. Usual care consisted of information resources (an OA booklet from Arthritis Research UK (now Versus Arthritis),[23] and a handout about local services/support groups/activities). The intervention group participants received the information resources during their initial mentorship session and were offered the opportunity to discuss the resources with their peer mentor.



For peer review only - http://bmjopen.bmj.com/site/about/guidelines.xhtml

Control group

Control group participants received usual care only. The control group participants received the information resources during their baseline researcher visit and were offered the opportunity to discuss the resources with the researcher.

**OA peer mentorship intervention**

Intervention development

The OA peer mentorship intervention was developed in two stages. The first stage included a rapid review of published primary studies investigating one-to-one peer support interventions. The review aimed to identify: the range of methods and approaches used in delivering peer support interventions; training and support approaches for peer mentors involved in intervention delivery; and challenges of developing and implementing face-to-face peer support to improve self-management of long-term conditions. Using Medline, CINAHL & PsycInfo, a search of the literature between 2007 and 2018 was undertaken using the terms and synonyms *'peer support'*, *'long term condition'* and *'intervention'*. Thirteen papers were included [16, 24-35]. The findings highlighted the importance of encouraging a person-centred approach and retaining flexibility within the peer mentorship sessions. There was little information about 'matching' the mentee with the peer mentor, although some studies based this on factors such as gender and age. There were issues around recruitment of peer mentors and a need to provide support and guidance to peer mentors throughout the intervention.



For peer review only - http://bmjopen.bmj.com/site/about/guidelines.xhtml

A preliminary version of the OA peer mentorship intervention was developed based on the rapid review findings and the following sources:

- Guidelines on self-management and OA from organisations such as the United Kingdom (UK) National Institute for Health and Care Excellence (NICE) and the UK charity Versus Arthritis.
- The ‘Staying Connected Programme’ – an arthritis self-management programme previously run by Arthritis Care Northern Ireland (now Versus Arthritis Northern Ireland).[36]
- Input from project team members, including a consultant rheumatologist, a health psychologist and a physiotherapist specialising in activity pacing/chronic pain management.

The above sources were also used to develop a draft peer mentor educational resource pack. The pack was designed to supplement peer mentor training sessions and be used a resource during mentorship sessions. The pack included a range of handouts that peer mentors could give to participants.

The second stage of the development process consisted of expert review sessions conducted with the following key stakeholders: study Patient and Public Involvement (PPI) members (n=2); other older individuals with OA (n=5); health professionals (n=4); voluntary/community organisation representatives (n=4); and researchers (n=2). Review sessions were conducted face-to-face (group, paired or individual meetings), via telephone or via email. The stakeholders were provided with information about the provisional OA peer mentorship intervention and a copy of the

For peer review only



For peer review only - http://bmjopen.bmj.com/site/about/guidelines.xhtml

draft educational pack. Stakeholders' opinions of these were assessed using a pro forma.

Key refinements made based on the expert review sessions included:

- Peer mentors were encouraged not cover too many topics in the first mentorship session to allow more time for developing rapport and managing participant expectations.
- Participants were provided with notebooks for recording goals, reflections and additional notes.
- The language used in the educational pack was simplified.
- Additional infographic handouts and further information on falls, local authority services and emotional well-being were added to the educational resource pack.

Finalised intervention

The finalised OA peer mentorship intervention aimed to improve participants' health outcomes through increasing their engagement with self-management behaviours. Figure 1 presents a logic model of the intervention, including the proposed mechanisms of action. The intervention involved up to eight one-hour self-management support sessions delivered by a trained peer mentor. During the sessions, the peer mentor provided guided support that incorporated multiple behaviour change techniques (BCTs) and covered a combination of core and optional topics (figure 1). In line with a person-centred approach, the implementation of BCTs and the choice and order of topics covered was flexible and participant-led.



For peer review only - http://bmjopen.bmj.com/site/about/guidelines.xhtml

However, peer mentors were encouraged to cover all core topics at least once and set/review goals with the participant weekly. Online supplementary table 3 provides examples of the implementation of the key BCTs employed.

The peer mentorship sessions took place approximately weekly in the participant's home or another private location of their choice. A person-centred approach was emphasised throughout.[37, 38] Therefore, the number of sessions and scheduling were flexible.

The Volunteer Coordinator attended the start of each initial session to introduce the participant and peer mentor; remind them of the trial and intervention aims; and answer questions. The remainder of the initial session and all subsequent sessions were undertaken by the mentor alone. The Volunteer Coordinator contacted the participant and peer mentor following the initial session to check they were happy to continue with subsequent sessions with their mentorship match. Peer mentors completed a 'session summary' in writing following each session, detailing the topics covered during that session, any challenges encountered and their reflections on the progress made.

Peer mentor eligibility and recruitment

Peer mentors were trained volunteers aged ≥50 years old with hip and/or knee OA who were able to travel independently. Multiple approaches were used to recruit peer mentors, including printed/social media advertisements and sharing trial information at local support/activity groups.



For peer review only - http://bmjopen.bmj.com/site/about/guidelines.xhtml

Potential peer mentors were asked to complete an application form, supply two references and take part in a telephone interview with the Volunteer Coordinator. Those assessed as suitable were invited to attend a compulsory two-day training event. Prior to being matched with a participant, all peer mentors were required to complete enhanced Disclosure and Barring Service (criminal record) checks,[39] for safeguarding purposes.

Peer mentor training and matching

Three training events were held due to varying peer mentor availability and staggering of the mentor recruitment, which meant it was not possible to train all mentors in one event. The first training event was provided by two external facilitators from Arthritis Care Northern Ireland and a trial team member (physiotherapist specialising in activity pacing/chronic pain management). The remaining events were provided by three trial team members (Volunteer Coordinator, the physiotherapist who facilitated the first training event and another musculoskeletal physiotherapist). Feedback from the first event was used to refine the content/delivery of the subsequent events.

The training events involved presentations and interactive activities covering OA self-management topics, mentorship skills and the practicalities of the peer mentor role (online supplementary table 4).

For peer review only



For peer review only - http://bmjopen.bmj.com/site/about/guidelines.xhtml

Participant/mentor matching was undertaken by the Volunteer Coordinator and the researcher who completed the baseline researcher visit. The initial intention was to base matching on location, gender preference, age and OA site(s). However, due to peer mentors' preferences and discrepancies in the availability of intervention group participants and trained peer mentors, these criteria were relaxed.

**Data collection and analysis**

Feasibility outcomes

This trial's primary focus was on assessing the feasibility outcomes specified in table 1. Four associated success criteria for proceeding to a definitive RCT were pre-specified based on relevant guidance,[40] (table 1).



For peer review only - http://bmjopen.bmj.com/site/about/guidelines.xhtml

**Table 1: Feasibility outcomes and success criteria for proceeding to a definitive randomised controlled trial**

| Feasibility outcome | Details of feasibility outcome | Success criterion |
|---|---|---|
| Peer mentor recruitment rate | Number of peer mentors trained divided by the number individuals who were eligible for the peer mentor role | 1. Demonstration that the intervention can be delivered in practice and is acceptable to participants and peer mentors[a] |
| Peer mentor attrition rate | Number of peer mentors who were not matched with a participant divided by the number of peer mentors trained | |
| Intervention completion rate | Number of participants who completed at least three peer mentorship sessions divided by the number of participants who commenced the intervention | |
| Participant recruitment rate | Number of participants randomised divided by the number of individuals who were eligible to participate | 2. ≥60% of eligible individuals recruited |
| Attrition rate overall and in each group | Number of participants who did not complete the 6 month questionnaire divided by the number of participants randomised | 3. <20% participant attrition with no evidence of attrition bias |
| Sample size required for a definitive RCT | Sample size calculation using the feasibility trial PIH scale scores assuming: 5% significance level; minimum power of 0.8; and 1:1 allocation ratio. The PIH scale scores were used because the PIH scale was chosen as the provisional primary outcome measure. | 4. Calculation of a sample size that is achievable in a main trial |
| Questionnaire completion rate | Number of questionnaires completed divided by the number of participants provided with the questionnaires | None specified for these feasibility outcomes |
| Intervention fidelity | Content analysis of the mentors' session summaries | |
| Cost of delivering the mentor training event | Estimation based on the events delivered by the trial team members accounting for: staff salary, National Insurance and pension contributions; resources; refreshments; and peer mentor travel expenses; but not venue hire, estates and indirect costs | |
| Cost of delivering the intervention | Estimation based on the Volunteer Coordinator and peer mentor costs, excluding the training costs | |

For peer review only

**Table 1 Footnote**

PIH scale, Revised 12-Item Partners In Health scale; RCT, randomised controlled trial.

[a] No minimum/maximum rates were pre-specified as acceptable for this success criterion.

Patient-reported outcomes

Patient-reported outcomes were assessed using paper self-report questionnaires administered at baseline, eight weeks and six months. The baseline questionnaires were administered by a researcher or the Volunteer Coordinator. The majority of baseline questionnaires were administered after the participant had been informed of their group allocation. This approach was chosen due to the fluctuating availability of trained peer mentors. This meant there was sometimes a delay between participants being allocated to the intervention group and being able to commence their mentorship sessions. Therefore, most intervention group participants completed the baseline questionnaire with the Volunteer Coordinator immediately prior to their initial mentorship session to avoid a delay between completion of the baseline questionnaire and commencement of their mentorship sessions.

The eight-week and six-month questionnaires were administered and returned via post. Non-responders were followed up with a second questionnaire posting two weeks later and a telephone call four weeks later.



For peer review only - http://bmjopen.bmj.com/site/about/guidelines.xhtml

The questionnaires covered participants' sociodemographic and clinical characteristics and included validated tools assessing the biomedical/psychosocial outcomes specified below.

Provisional primary outcome measure

- Revised 12-Item Partners in Health (PIH) Scale: 12-item scale that assesses chronic condition self-management.[41] Each item is scored on a 9-point Likert scale (0-8). Scores are summed to give a total score (0-96). Higher scores indicate higher self-management knowledge/behaviours.

Provisional secondary outcome measures

- Multidimensional Scale of Perceived Social Support (MSPSS): 12-item scale that assesses perceived social support from a significant other, family and friends.[42] Each item is scored on a 7-point Likert scale (1-7) and the mean for all items is calculated. Higher scores indicate greater perceived social support.
- Western Ontario and McMaster's University Osteoarthritis Index Likert version (WOMAC® 3.1): 24-item disease-specific scale with three subscales that assess pain (5 items), stiffness (2 items) and physical function (17 items).[43] Each item is scored on a 5-point Likert scale (0-4). Scores are summed to give a total score (0-96) and subscale scores (pain: 1-20; stiffness: 0-8; physical function: 0-68). Higher scores indicate more severe problems.



For peer review only - http://bmjopen.bmj.com/site/about/guidelines.xhtml

- 8-item Arthritis Self-Efficacy Scale English Version (ASES-8): 8-item scale that assesses self-efficacy for managing arthritis symptoms.[44-46] Each item is scored on a 10-point Likert scale (1-10) and the mean for all items is calculated. Higher scores indicate greater self-efficacy.
- Hospital Anxiety and Depression Scale (HADS): 14-item scale with two subscales that assess symptoms of anxiety (7 items) and depression (7 items).[47] Each item is scored on a 4-point Likert scale (0-3). Scores are summed to give a total score (0-42) and subscale scores (0-21). Higher scores indicate more severe symptoms.
- EQ-5D-5L: Descriptive system and visual analogue scale (VAS) that assess general health status.[48, 49] The descriptive system includes five dimensions, each of which has five response levels. Each response is converted to a single-digit number. The numbers for each of the five dimensions can then be converted to a single index value anchored at 0 (a state equivalent to dead) and 1 (full health). The VAS consists of a single score on a scale from 0 (worst health imaginable) to 100 (best health imaginable).

The eight-week and six-month questionnaires included sections on healthcare and community resource use, adapted from the Client Services Receipt Inventory (CSRI).[50]

Missing data were addressed in line with guidance for the relevant outcome measurement tool. The quantitative questionnaire responses were analysed using STATA (version 15). Given the trial was not powered to detect statistically significant

For peer review only

differences the analyses were predominantly descriptive. However, exploratory analyses of the outcome measure data and healthcare resource use data were performed. The outcome scores were tested for normality at the 5% significance level.[51] Between-group comparisons were made using ANCOVA models and, where the scores were significantly skewed, quantile regression models were used.[52]

**Patient and Public Involvement**

Patient and Public Involvement (PPI) played a key role in this trial as follows:

- During the trial development stage, six individuals with OA were consulted for research ideas and eight members of a local musculoskeletal PPI group participated in a group discussion. These patient representatives emphasised that one-to-one mentorship would be preferable to a group-based approach and felt the questionnaire burden was appropriate.
- Three patient representatives became 'study PPI members' and assisted with the conduct of the study, reviewing lay materials and finalising the dissemination plans. These PPI members were also invited to attend peer mentorship training sessions.
- Two study PPI members and five other individuals with OA helped refine the provisional intervention and educational resources through expert review sessions.
- The peer mentors were all trained volunteers with hip and/or knee OA. Peer mentors were encouraged to give regular informal feedback about their

For peer review only



For peer review only - http://bmjopen.bmj.com/site/about/guidelines.xhtml

involvement in the trial, as well as being invited to participate in a nested qualitative study (reported elsewhere).

## RESULTS

### Feasibility outcomes

Participant flow and intervention completion

Participant recruitment took place between 22nd November 2018 and 30th May 2019. The final questionnaire was completed on the 16th February 2020. At least 345 individuals were approached (figure 2). Ninety-six individuals were screened, of whom 65 were eligible. The commonest reason for ineligibility was being listed for joint replacement (n=8). Fifty individuals were randomised. Therefore, the participant recruitment rate was 77%. The commonest reason for declining participation was insufficient time/other commitments (n=7). Most participants were recruited through the primary care physiotherapy department (n=44).

One intervention group participant withdrew due to ill-health prior to completing the baseline questionnaire and commencing the intervention. Of the 24 participants who commenced the intervention, 20 completed at least three mentorship sessions (figure 2). The intervention completion rate was therefore 83%. Reasons for discontinuing the intervention largely related to participants feeling the intervention was not relevant to their needs, for example, due to having mild OA symptoms

For peer review only

and/or feeling they did not require additional self-management support. The mean number of mentorship sessions received was 5.79 (SD=2.25; median=7). No harms/unintended effects related to the intervention occurred.

One intervention participant who discontinued the intervention after two mentorship sessions withdrew (no reason provided). One additional intervention participant who discontinued the intervention after two mentorship sessions did not complete the follow-up questionnaires. One control group participant did not complete the follow-up questionnaires. A family member of this control group participant returned his six-month questionnaire indicating he had died. The attrition rates were therefore: 8% overall; 12% in the intervention group; and 4% in the control group.

For peer review only

### Questionnaire completion

Amongst the participants who were provided with the questionnaires, the questionnaire completion rate for both groups was 100% at baseline and 96% at eight weeks and six months (figure 2). Minor issues were noted with the resource use sections. Some participants duplicated details, for example, by recording the same physiotherapy appointments under ‘Hospital services’ and ‘Services outside the hospital’. Additionally, the ‘day activities’ reported by participants largely related to their hobbies, such as yoga and dancing, rather than social/community support activities.

### Peer mentor flow and characteristics

Peer mentor recruitment took place between May 2018 and January 2019. Thirty-two individuals enquired about the role, of whom 21 were eligible (online supplementary figure 1). Five eligible individuals were unable to attend the training events, one withdrew due to ill-health and 15 were trained. The peer mentor recruitment rate was therefore 71%. Five trained mentors withdrew prior to being matched, most commonly due to ill-health (n=3). The peer mentor attrition rate was therefore 33%. The most successful peer mentor recruitment approach was a local community magazine advertisement, which led to recruitment of five active peer mentors.

Nine active peer mentors were female and one was male. Their mean age was 68 years old (SD=5.5; range=57-75). All had been living with hip and/or knee OA for at least three years. Nine were retired, although three had only recently stopped work, and one worked full-time. Each active peer mentor supported between one and four participants (online supplementary figure 1).

Session summary data

The session summaries indicated the following topics were covered by almost all participant/mentor dyads and were the most frequently covered overall: Learning about OA; Goal setting; Pacing; Muscle strengthening; and Pain management. In addition, Getting active and Eating well were frequently covered.

The least frequently covered topics were: Getting connected with friends/services; and Effective communication. Correspondingly, these topics were covered by only



For peer review only - http://bmjopen.bmj.com/site/about/guidelines.xhtml

six and eight dyads respectively. Positive thinking and Sleep quality were also rarely covered.

Intervention costs

The estimated cost of delivering the training event to five peer mentors (mean number of mentors per event) was £239 per mentor (online supplementary table 5). The estimated cost of delivering the intervention based on the mean number of 5.79 mentorship sessions was £274 per participant (online supplementary table 6).

Sample size calculation

Based on the assumptions stated in table 1, between-groups variance and within-groups variance of the PIH scale at six months of 369.1 and 6237.1 respectively and detecting a difference in the PIH scale of 4.4 (effect size at six months), the sample size required for a definitive RCT was calculated as 136 participants (68 per group). Retaining the same attrition rate as for the feasibility trial (20%) would give a required sample size of 170 (85 per group).

**Patient-reported outcomes**

Participant baseline characteristics

Table 2 provides a summary of participants' baseline sociodemographic and clinical characteristics. Additional details are available in online supplementary table 7.

For peer review only



For peer review only - http://bmjopen.bmj.com/site/about/guidelines.xhtml

**Table 2: Participant baseline characteristics**

| Characteristics | Intervention n=24 | Control n=25 |
|---|---|---|
| Age in years, mean (SD) | 70.0 (8.6) | 69.3 (8.1) |
| Men, n (%) | 10 (41.7) | 4 (16.0) |
| Ethnicity, white, n (%) | 21 (87.5) | 24 (96.0) |
| Further Education[a], n (%) | 15 (62.5) | 15 (60.0) |
| Employed, n (%) | 7 (29.2) | 5 (20.0) |
| Retired, n (%) | 17 (70.8) | 20 (80.0) |
| Body mass index in kg/m$^2$, mean (SD) | 27.5 (6.3) | 28.5 (5.5) |
| Duration of arthritis diagnosis in years, mean (SD) | 4.7 (5.2) | 5.3 (6.2) |
| At least one hip affected by arthritis, n (%) | 14 (58.3) | 13 (52.0) |
| At least one knee affected by arthritis, n (%) | 22 (91.7) | 21 (84.0) |
| VAS for current pain due hip/knee arthritis, mean (SD) | 52.8 (26.8) | 48.6 (26.3) |
| VAS for ability to cope with/manage arthritis in general, mean (SD) | 66.2 (21.8) | 64.4 (21.8) |
| Number of joints affected by pain for > 6 weeks in the last 3 months, median (IRQ) | 5 (4) | 3 (3) |
| Number of comorbidities[b], n (%) | | |
| 0 | 8 (33.3) | 6 (24.0) |
| 1 | 5 (20.8) | 10 (40.0) |
| 2 | 5 (20.8) | 3 (12.0) |
| ≥3 | 6 (25.0) | 6 (24.0) |

**Table 2 Footnote**

SD, standard deviation; VAS, Visual Analogue Scale (0-100mm)

[a] Further education was defined as any formal education undertaken once the participant was aged over 16 years old.

For peer review only

[b] Comorbidities include: diabetes; asthma; bronchitis; gastrointestinal problems; angina/heart problems; high blood pressure; depression; and anxiety.

Provisional primary outcome

The mean PIH scores at baseline were 73.9 and 76.5 for the intervention and control groups respectively (table 3). After eight weeks, the mean PIH score improved in the intervention group (mean change=2.6; SD=13.2), and deteriorated in the control group (mean change= -7; SD=12.3). Thus, the intervention group participants appeared to have better self-management knowledge/behaviours than the control group participants at eight weeks (effect size=8.3; 95% CI: 2.2 to 14.4) (table 4). At six months, the intervention group's mean PIH score was similar to the group's score at eight weeks, whilst the control group's mean PIH score increased but remained lower than at baseline. Overall, the intervention group had higher PIH scores than the control group after six months (effect size=4.4; 95% CI: -2.8 to 11.6) (table 4).



For peer review only - http://bmjopen.bmj.com/site/about/guidelines.xhtml

**Table 3: Outcome measurement scores at baseline, 8 weeks and 6 months**

| Outcome measure | Baseline means (SD) | | 8 week means (SD) | | 6 month means (SD) | |
|---|---|---|---|---|---|---|
| | Intervention (n=22) | Control (n=24) | Intervention (n=22) | Control (n=24) | Intervention (n=22) | Control (n=24) |
| PIH scale | 73.9 (16.3) | 76.5 (14.3) | 76.5 (14.5) | 69.5 (10.6) | 76.0 (13.4) | 73.0 (15.0) |
| MSPSS | 5.3 (1.1) | 5.7 (0.98) | 4.9 (1.6) | 5.2 (1.6) | 4.9 (1.3) | 5.4 (1.4) |
| WOMAC Total[a] | 32.6 (18.8) | 41.9 (19.5) | 34.0 (17.8) | 43.5 (19.4) | 34.7 (18.5) | 41.3 (17.8) |
| WOMAC Pain[a] | 6.8 (4.5) | 9 .0 (4.2) | 7.5 (3.9) | 9.6 (4.1) | 7.7 (4.0) | 8.9 (3.2) |
| WOMAC Stiffness[a] | 3.7 (1.6) | 4.3 (1.9) | 3.3 (1.3) | 4.3 (1.6) | 3.4 (1.3) | 4.0 (1.7) |
| WOMAC Function[a] | 22.1 (14.0) | 28.5 (14.6) | 23.2 (13.2) | 29.6 (15.3) | 23.6 (14.8) | 28.3 (13.5) |
| ASES-8 | 6.0 (1.9) | 5.9 (1.7) | 5.5 (2.2) | 6.5 (2.4) | 6.3 (1.6) | 5.3 (2.5) |
| HAD Total[a] | 9.4 (4.4) | 11.3 (7.2) | 10.5 (5.7) | 12.7 (8.3) | 10.7 (6.2) | 12.8 (8.4) |
| HADS Anxiety[a] | 5.3 (3.1) | 6.7 (4.3) | 6.3 (3.4) | 7.3 (4.8) | 5.9 (3.9) | 7.5 (4.9) |
| HADS Depression[a] | 4.1 (2.0) | 4.6 (3.6) | 4.3 (3.1) | 5.4 (4.0) | 4.8 (2.7) | 5.3 (4.0) |
| EQ-5D-5L Index[bc] | 0.723 (0.139) | 0.645 (0.170) | 0.691 (0.099) | 0.647 (0.249) | 0.687 (0.088) | 0.647 (0.183) |
| EQ-5D-5L VAS[c] | 70 (15.0) | 75 (20.0) | 70 (25.0) | 70 (22.5) | 75 (20.0) | 70 (23.5) |

**Table 3 Footnote**

ASES-8, 8-item Arthritis Self-Efficacy Scale English version; HADS, Hospital Anxiety and Depression Scale; MSPSS, Multidimensional Scale of Perceived Social Support; PIH scale, Revised 12-Item Partners in Health scale; VAS, Visual Analogue Scale; WOMAC, Likert version of the Western Ontario and McMaster's University Osteoarthritis Index.

For peer review only

[a] Higher scores indicate more severe problems.

[b] EQ-5D-5L Index value control group n=23 due to missing data.

[c] Median (inter-quartile range).

**Table 4: Outcome measurements score changes and effect of the intervention at 8 weeks and 6 months**

| Outcome measure | Changes at 8 weeks | | Effect size at 8 weeks, mean (95% CI)[c] | Changes at 6 months | | Effect size at 6 months, mean (95% CI)[c] |
|---|---|---|---|---|---|---|
| | Intervention | Control | | Intervention | Control | |
| PIH scale | 2.6 (13.2) | -7.0 (12.3) | 8.3 (2.2-14.4) | 2.1 (10.6) | -3.5 (16.6) | 4.4 (-2.8-11.6) |
| MSPSS | -0.28 (1.4)[d] | -0.25 (1.2)[d] | 0.0 (-0.80-0.80)[e] | -0.42 (0.93)[d] | -0.06 (0.6)[d] | -0.40 (-0.84-0.03)[e] |
| WOMAC Total[a] | 3.0 (19)[d] | 5.0 (22)[d] | -2.6 (-10.2-4.9)[e] | 2.1 (8.6) | -0.59 (19.1) | -0.65 (-8.9-7.6) |
| WOMAC Pain[a] | 0.64 (2.6) | 0.63 (3.7) | -0.75 (-2.5-1.0) | 0.91 (2.4) | -0.09 (3.8) | 0.06 (-1.6-1.7) |
| WOMAC Stiffness[a] | -0.36 (1.5) | -0.04 (1.1) | -0.59 (-1.3-0.08) | -0.27 (1.8) | -0.29 (1.2) | -0.31 (-1.1-0.46) |
| WOMAC Function[a] | 1.1 (7.8) | 1.0 (10.4) | -1.3 (-6.7-4.1) | 1.5 (7.8) | -0.21 (15.4) | -0.8 (-7.7-6.0) |
| ASES-8 | 0.48 (1.6) | -0.04 (2.25) | 0.64 (-0.51-1.8) | 0.69 (3.0)[d] | 0.0 (3.3)[d] | 0.34 (-1.3-1.9)[e] |
| HADS Total[a] | 1.12 (3.8) | 1.38 (4.5) | -0.27 (-2.8-2.3) | 1.33 (3.4) | 1.5 (4.3) | -0.01 (-2.4-2.4) |
| HADS Anxiety[a] | 0.95 (2.8) | 0.58 (2.1) | 0.26 (-1.2-1.7) | 0.59 (2.4) | 0.79 (2.6) | -0.23 (-1.8-1.3) |
| HADS Depression[a] | 0.20 (2.4) | 0.79 (3.4) | -0.74 (-2.5-1.0) | 0.74 (1.6) | 0.67 (2.5) | 0.03 (-1.3-1.3) |
| EQ-5D-5L Index[b] | -0.057 (0.20) | 0 (0.12) | -0.06 (-0.18-0.06) | -0.053 (0.14) | 0 (0.19) | -0.04 (-0.12-0.04) |
| EQ-5D-5L VAS | -2.5 (20)[d] | 0 (10)[d] | 0 (-10.8-10.8)[e] | 0 (20)[d] | -5 (21)[d] | 6.7 (-4.6-18.0)[e] |

**Table 4 Footnote**



For peer review only - http://bmjopen.bmj.com/site/about/guidelines.xhtml

ASES-8, 8-item Arthritis Self-Efficacy Scale English version; CI, Confidence interval; HADS, Hospital Anxiety and Depression Scale; MSPSS, Multidimensional Scale of Perceived Social Support; PIH scale, Revised 12-Item Partners in Health scale; WOMAC, Likert version of the Western Ontario and McMaster's University Osteoarthritis Index.

[a] Higher scores indicate more severe problems.

[b] EQ-5D-5L Index value control group n=23 due to missing data.

[c] ANCOVA models adjusted for baseline outcomes.

[d] Median (inter-quartile range).

[e] Median (95% confidence intervals).

For peer review only



For peer review only - http://bmjopen.bmj.com/site/about/guidelines.xhtml

Provisional secondary outcomes

There were no significant between-group differences for any of the secondary outcomes at eight weeks or six months. However, the mean WOMAC and HADS scores were lower in the intervention group than the control group at baseline and both follow-up time-points.

Resource use

Healthcare resource use between baseline and six months was largely similar between groups. The main differences were more overnight hospital stays in the control group (mean difference between groups= -0.91; 95% CI: -1.78 to -0.04), and more GP Practice Nurse visits in the intervention group (mean difference between groups=0.93; 95% CI: 0.15 to 1.71) (online supplementary table 8). No participants reported attending a day centre. Two intervention group participants and four control group participants reported receiving help with daily activities.

## DISCUSSION

This feasibility trial aimed to develop and trial a peer mentorship intervention to improve OA self-management. All four pre-specified success criteria for proceeding to a definitive RCT (table 1) were achieved. The peer mentor recruitment rate (71%), peer mentor attrition rate (33%) and intervention completion rate (83%) all compare favourably with previous peer mentorship studies.[16, 32, 53, 54] This suggests the OA peer mentorship intervention can be delivered in practice and is acceptable to



For peer review only - http://bmjopen.bmj.com/site/about/guidelines.xhtml

participants and peer mentors. However, some participants discontinued the intervention due to having mild OA symptoms and feeling they did not need self-management support. This could be overcome by specifying a minimum symptom severity threshold and/or a maximum arthritis-related self-efficacy threshold in the eligibility criteria to prevent inclusion of individuals who have mild symptoms and/or already feel confident about managing their symptoms.

The participant recruitment rate was 77% and hence well above the pre-specified minimum threshold of 60%. However, this recruitment rate did not account for individuals lost prior to screening. The overall participant attrition rate at 6 months was 8% and hence well below the pre-specified maximum threshold of 20%. This suggests that additional strategies to improve participant recruitment and retention would not be needed for a future RCT. Allowing for 20% attrition, the sample size required for a definitive RCT was calculated as 170 participants (85 per group). Assuming each peer mentor supports two participants, this would require 43 mentors. This target RCT sample size is achievable. However, only ten active peer mentors were recruited during this trial. Additional strategies would therefore be needed to recruit sufficient mentors. These could include using multiple recruitment sites across a wider geographical area, recruiting mentors over a longer time-period, using snowballing recruitment with previously trained peer mentors and optimising recruitment through media outlets, such as magazine advertisements.

The findings for the remaining feasibility outcomes were also encouraging. The questionnaire completion rates were consistently high (96%-100%). However, minor modifications to the resource use sections would be beneficial to avoid participants

For peer review only



For peer review only - http://bmjopen.bmj.com/site/about/guidelines.xhtml

duplicating details/reporting unnecessary details. The session summary data indicated intervention fidelity was good overall, with most participant/mentor dyads covering four of the five core topics. The remaining core topic, Getting Connected with friends/services, was only covered by six dyads. This may be because the participants already had good social support networks, as suggested by their relatively high MSPSS scores. Additionally, most dyads covered the optional topic of Pain management. Therefore, changing Getting connected to an optional topic and Pain management to a core topic warrants consideration.

The estimated costs of training peer mentors and delivering the intervention were £239 per mentor and £274 per participant respectively. The training costs would have been lower if fewer training events had been run with more mentors per event. This was not possible due to varying peer mentor availability and staggering of the mentor recruitment. A similar issue was noted in a study of peer mentorship for people with advanced cancer,[54] and would need accounting for in a future RCT.

This trial included exploratory analyses of the impact of the OA peer mentorship intervention on patient-reported outcomes. These analyses provide a preliminary insight into the potential effectiveness of the intervention. However, the results must be interpreted cautiously because the present trial was not powered to detect statistically significant differences. The exploratory analyses suggest the OA peer mentorship intervention may improve self-management knowledge/behaviours (table 4). However, no significant effects of the OA peer mentorship intervention on other outcomes, such as pain and arthritis-related self-efficacy, were observed in the present trial.



For peer review only - http://bmjopen.bmj.com/site/about/guidelines.xhtml

A Cochrane review identified low to moderate quality evidence indicating self-management education programmes for people with OA do not result in any clinically meaningful benefits.[55] Despite this, the authors concluded that trials investigating alternative self-management support approaches, particularly those involving tailored support, are warranted. One such approach is the Staying Connected Programme – a tailored one-to-one eight-week self-management programme delivered by trained volunteers.[36] A recent quasi-experimental study identified significant improvements in pain and arthritis-related self-efficacy amongst individuals with arthritis who participated in this programme.[36] The Staying Connected Programme volunteers were not required to have an arthritis diagnosis, despite peer support being recognised as a valuable approach for supporting self-management.[11, 12] In addition, the Staying Connected Programme aimed to support individuals with various types of arthritis and other long-term conditions [36]. The present trial's intervention therefore aimed to replicate some key elements of the Staying Connected Programme whilst also incorporating peer support and being tailored specifically to the needs of people with OA. Although there are disparities between the findings of the present trial and the Staying Connected Programme study, neither was an adequately powered RCT. Future work is therefore required to determine the effectiveness of both the present trial's OA peer mentorship intervention and the Staying Connected Programme.

In addition to drawing on the Staying Connected Programme, the development of the OA peer mentorship intervention incorporated multiple other sources and an expert review with key stakeholders. This approach, combined with extensive PPI, helped



For peer review only - http://bmjopen.bmj.com/site/about/guidelines.xhtml

ensure the intervention is feasible, acceptable and focused on the needs of individuals with OA. The substantial investment in the development process will also maximise the chances of the intervention proving effective during a future definitive RCT. A potential limitation is that the development process was not based on a single behaviour change theory or theoretical framework. However, the broad range of sources considered and input from multidisciplinary experts helped ensure that the intervention has a sound theoretical basis (figure 1). In particular, the focus on enhancing self-efficacy is consistent with other peer support interventions aimed at improving chronic condition self-management.[24, 32, 56, 57]

This trial also presents additional limitations. Notably, 88% of participants were recruited through a physiotherapy service and hence had already received some self-management support. This could be addressed through using alternative recruitment sites, such as GP practices. Furthermore, the majority of participants were older, white, well-educated females and a Cochrane review identified that the impact of self-management education programmes for OA may vary between Caucasian, educated females and other subgroups.[55] Additionally, peer support interventions may be most effective amongst the 'hardly reached', such as individuals with lower education levels.[58] Targeting the OA peer mentorship intervention to specific subgroups could therefore be valuable. Participants and peer mentors' experiences of the OA peer mentorship intervention were explored through a nested qualitative study (reported elsewhere). However, the impact of providing the intervention on the peer mentors was not quantitatively assessed. This is an important consideration because providing peer mentorship may have positive and/or negative effects on peer mentors.[11, 26] Another limitation of the present trial

For peer review only



For peer review only - http://bmjopen.bmj.com/site/about/guidelines.xhtml

was that most participants were aware of their group allocation when completing the baseline questionnaire, which may have influenced their questionnaire responses.[59] This could be overcome in a future definitive RCT by ensuring a greater number of trained peer mentors are available and/or administering the baseline questionnaire by post/online.

In conclusion, this trial's findings suggest peer mentorship is a feasible, acceptable and promising approach for improving OA self-management. Further investigation of the OA peer mentorship intervention is therefore warranted. However, minor modifications to the intervention and trial procedures should be considered, particularly regarding the participant and peer mentor recruitment procedures.

## ACKNOWLEDGEMENTS

The Research team acknowledges the support of the National Institute for Health Research Clinical Research Network (NIHR CRN) and are grateful to all participants and peer mentors who were part of the study; and to the trial PPI members and expert panel group.

## FOOTNOTES

**Twitter:** @LeedsBRC @_Anna_Anderson



For peer review only - http://bmjopen.bmj.com/site/about/guidelines.xhtml

**Author contributions:** GAM contributed to the conception and design of the study; acquisition, analysis and interpretation of data; and drafting the manuscript. LM, PGC, SRK and DA contributed to the design of the study. AMA contributed to the acquisition, analysis and interpretation of data and drafting the manuscript. ECL contributed to the acquisition and interpretation of data. EDR contributed to the acquisition of data. GR contributed to the design of the study and the interpretation of data. TFM contributed to the analysis of data. All authors have been involved in revising the work for important intellectual content and have approved the final version for publication.

**Funding:** This paper presents independent research funded by the National Institute for Health Research (NIHR) under its Research for Patient Benefit (RfPB) Programme (Grant Reference Number PB-PG-1215-20012). PGC and SRK are supported in part by the NIHR Leeds Biomedical Research Centre.

**Disclaimer:** The views expressed are those of the authors and not necessarily those of the NIHR or the Department of Health and Social Care.

**Competing interests:** None declared.

**Patient consent for publication:** Not required.

**Ethics approval:** Ethical approval was gained from the Greater Manchester South Research Ethics Committee (Reference:17/NW/0238). All participants provided written informed consent prior to participating in the trial.

For peer review only



For peer review only - http://bmjopen.bmj.com/site/about/guidelines.xhtml

**Data sharing agreement:** Deidentified participant data and the trial protocol are available from the corresponding author via A.Anderson@leeds.ac.uk upon reasonable request. Reuse is permitted for the purpose of health and care research as long as the original creators are acknowledged.

## REFERENCES

1. James SL, Abate D, Abate KH, et al. Global, regional, and national incidence, prevalence, and years lived with disability for 354 diseases and injuries for 195 countries and territories, 1990-2017: a systematic analysis for the Global Burden of Disease Study 2017. *Lancet* 2018;392(10159):1789-858. doi: 10.1016/S0140-6736(18)32279-7.
2. Turkiewicz A, Petersson IF, Bjork J, et al. Current and future impact of osteoarthritis on health care: a population-based study with projections to year 2032. *Osteoarthritis Cartilage* 2014;22(11):1826-32. doi: 10.1016/j.joca.2014.07.015.
3. Hunter DJ, Schofield D, Callander E. The individual and socioeconomic impact of osteoarthritis. *Nat Rev Rheumatol* 2014;10(7):437-41. doi: 10.1038/nrrheum.2014.44.
4. Smith TO, Purdy R, Lister S, et al. Living with osteoarthritis: a systematic review and meta-ethnography. *Scand J Rheumatol* 2014;43(6):441-52. doi: 10.3109/03009742.2014.894569.



For peer review only - http://bmjopen.bmj.com/site/about/guidelines.xhtml

5. Puig-Junoy J, Ruiz Zamora A. Socio-economic costs of osteoarthritis: A systematic review of cost-of-illness studies. *Semin Arthritis Rheum* 2015;44(5):531-41. doi: https://doi.org/10.1016/j.semarthrit.2014.10.012.
6. Nelson AE, Allen KD, Golightly YM, et al. A systematic review of recommendations and guidelines for the management of osteoarthritis: The chronic osteoarthritis management initiative of the U.S. bone and joint initiative. *Semin Arthritis Rheum* 2014;43(6):701-12. doi: 10.1016/j.semarthrit.2013.11.012.
7. Basedow M, Esterman A. Assessing appropriateness of osteoarthritis care using quality indicators: a systematic review. *J Eval Clin Pract* 2015;21(5):782-9. doi: 10.1111/jep.12402.
8. McHugh GA, Campbell M, Luker KA. Quality of care for individuals with osteoarthritis: a longitudinal study. *J Eval Clin Pract* 2012;18(3):534-41. doi: 10.1111/j.1365-2753.2010.01616.x.
9. Darlow B, Brown M, Thompson B, et al. Living with osteoarthritis is a balancing act: an exploration of patients' beliefs about knee pain. *BMC Rheumatol* 2018;2(1):15. doi: 10.1186/s41927-018-0023-x.
10. Hunter DJ, Bowden JL. Are you managing osteoarthritis appropriately? *Nat Rev Rheumatol* 2017;13(12):703-04. doi: 10.1038/nrrheum.2017.160.
11. Embuldeniya G, Veinot P, Bell E, et al. The experience and impact of chronic disease peer support interventions: A qualitative synthesis. *Patient Educ Couns* 2013;92(1):3-12. doi: https://doi.org/10.1016/j.pec.2013.02.002.
12. Fisher EB, Boothroyd RI, Elstad EA, et al. Peer support of complex health behaviors in prevention and disease management with special reference to


For peer review only



For peer review only - http://bmjopen.bmj.com/site/about/guidelines.xhtml

diabetes: systematic reviews. *Clin Diabetes Endocrinol* 2017;3(1):4. doi: 10.1186/s40842-017-0042-3.

13. Heisler M. Overview of Peer Support Models to Improve Diabetes Self-Management and Clinical Outcomes. *Diabetes Spectr* 2007;20(4):214-21. doi: 10.2337/diaspect.20.4.214.
14. Hunter DJ, Bierma-Zeinstra S. Osteoarthritis. *Lancet* 2019;393(10182):1745-59. doi: 10.1016/S0140-6736(19)30417-9.
15. Ali SA, Kokorelias KM, MacDermid JC, et al. Education and Social Support as Key Factors in Osteoarthritis Management Programs: A Scoping Review. *Arthritis* 2018;2018:2496190. doi: 10.1155/2018/2496190.
16. Simmons D, Cohn S, Bunn C, et al. Testing a peer support intervention for people with type 2 diabetes: a pilot for a randomised controlled trial. *BMC Fam Pract* 2013;14(1):5. doi: 10.1186/1471-2296-14-5.
17. Eldridge SM, Chan CL, Campbell MJ, et al. CONSORT 2010 statement: extension to randomised pilot and feasibility trials. *BMJ* 2016;355:i5239. doi: 10.1136/bmj.i5239.
18. Craig P, Dieppe P, Macintyre S, et al. Developing and evaluating complex interventions: new guidance. Medical Research Council; 2006.
19. National Institute for Health and Care Excellence. Osteoarthritis: care and management (Clinical guideline [CG177]), 2014.
20. Whitehead AL, Julious SA, Cooper CL, et al. Estimating the sample size for a pilot randomised trial to minimise the overall trial sample size for the external pilot and main trial for a continuous outcome variable. *Stat Methods Med Res* 2016;25(3):1057-73. doi: 10.1177/0962280215588241.

For peer review only



For peer review only - http://bmjopen.bmj.com/site/about/guidelines.xhtml

21. McHugh GA, Campbell M, Luker KA. GP referral of patients with osteoarthritis for consideration of total joint replacement: a longitudinal study. *Br J Gen Pract* 2011;61(589):e459-e68. doi: 10.3399/bjgp11X588420.

22. Sealed Envelope Ltd. Simple randomisation service 2019. https://www.sealedenvelope.com/simple-randomiser/v1/ (accessed 16th March 2020).

23. Arthritis Research UK. Osteoarthritis. Chesterfield, UK: Arthritis Research UK 2012.

24. Jerant A, Moore-Hill M, Franks P. Home-based, peer-led chronic illness self-management training: findings from a 1-year randomized controlled trial. *Ann Fam Med* 2009;7(4):319-27. doi: 10.1370/afm.996.

25. Chapin RK, Sergeant JF, Landry S et al. Reclaiming joy: pilot evaluation of a mental health peer support program for older adults who receive Medicaid. *Gerontologist* 2013 Apr;53(2):345-52. doi: 10.1093/geront/gns120.

26. Sandhu S, Veinot P, Embuldeniya G, et al. Peer-to-peer mentoring for individuals with early inflammatory arthritis: feasibility pilot. *BMJ Open* 2013;3(3). doi: 10.1136/bmjopen-2012-002267.

27. Simmons D, Prevost AT, Bunn C, et al. Impact of community based peer support in type 2 diabetes: a cluster randomised controlled trial of individual and/or group approaches. *PLoS One* 2015;10(3). doi: 10.1371/journal.pone.0120277.

28. Thom DH, Ghorob A, Hessler D, et al. Impact of peer health coaching on glycemic control in low-income patients with diabetes: a randomized controlled trial. *Ann Fam Med* 2013 Mar-Apr;11(2):137-44. doi: 10.1370/afm.1443.

For peer review only



For peer review only - http://bmjopen.bmj.com/site/about/guidelines.xhtml

29. Iliffe S, Kendrick D, Morris R, et al. Multicentre cluster randomised trial comparing a community group exercise programme and home-based exercise with usual care for people aged 65 years and over in primary care. *Health Technol Assess* 2014 Aug;18(49):vii-xxvii, 1-105. doi: 10.3310/hta18490.

30. Ayala GX, Ibarra L, Cherrington AL, et al. Puentes hacia una mejor vida (Bridges to a Better Life): Outcome of a Diabetes Control Peer Support Intervention. *Ann Fam Med* 2015;13 Suppl 1(Suppl 1):S9-S17. doi: 10.1370/afm.1807.

31. Matthias MS, McGuire AB, Kukla M, et al. A brief peer support intervention for veterans with chronic musculoskeletal pain: a pilot study of feasibility and effectiveness. *Pain Med* 2015;16(1):81-7. doi: 10.1111/pme.12571.

32. Cooper K, Jehu LM, Klein S, et al. Training peers to support older people with chronic low back pain following physiotherapy discharge: a feasibility study. *Physiotherapy* 2018:104(2):239-47. doi: 10.1016/j.physio.2017.07.001.

33. Cooper K, Schofield P, Klein S, Smith BH, Jehu LM. Exploring peer-mentoring for community dwelling older adults with chronic low back pain: a qualitative study. *Physiotherapy* 2017;103(2):138-45. doi: 10.1016/j.physio.2016.05.005.

34. Stevens Z, Barlow C, Iliffe S. Promoting physical activity among older people in primary care using peer mentors. *Prim Health Care Res Dev* 2015;16(2):201-6. doi: 10.1017/S1463423613000510.

35. Matthias MS, Kukla M, McGuire AB, et al. Facilitators and Barriers to Participation in a Peer Support Intervention for Veterans With Chronic Pain. *Clin J Pain* 2016;32(6):534-40. doi: 10.1097/AJP.0000000000000297.

For peer review only

36. McHugh GA, Conaghan PG, McConville M, et al. Promoting self-management in older people with arthritis: Preliminary findings of the Northern Ireland Staying Connected Programme. *Musculoskeletal Care* 2018;16(4):489-93. doi: 10.1002/msc.1353.

37. Olsson L-E, Jakobsson Ung E, Swedberg K, et al. Efficacy of person-centred care as an intervention in controlled trials – a systematic review. *J Clin Nurs* 2013;22(3-4):456-65. doi: 10.1111/jocn.12039.

38. nhs.uk. Involving people in their own care n.d. https://www.england.nhs.uk/ourwork/patient-participation/ (accessed 16th April 2020).

39. Crown copyright. Disclosure & Barring Service: About us: GOV.UK; n.d. https://www.gov.uk/government/organisations/disclosure-and-barring-service/about (accessed 27th February 2021).

40. Thabane L, Ma J, Chu R, et al. A tutorial on pilot studies: the what, why and how. *BMC Med Res Methodol* 2010;10(1):1. doi: 10.1186/1471-2288-10-1.

41. Smith D, Harvey P, Lawn S, et al. Measuring chronic condition self-management in an Australian community: factor structure of the revised Partners in Health (PIH) scale. *Qual Life Res* 2017;26(1):149-59. doi: 10.1007/s11136-016-1368-5.

42. Zimet GD, Dahlem NW, Zimet SG, et al. The Multidimensional Scale of Perceived Social Support. *J Pers Assess* 1988;52(1):30-41. doi: 10.1207/s15327752jpa5201_2.

43. Bellamy N, Buchanan WW, Goldsmith CH, et al. Validation study of WOMAC: a health status instrument for measuring clinically important patient relevant

For peer review only



For peer review only - http://bmjopen.bmj.com/site/about/guidelines.xhtml

outcomes to antirheumatic drug therapy in patients with osteoarthritis of the hip or knee. *J Rheumatol* 1988;15(12):1833-40.

44. Lorig K, Chastain RL, Ung E, et al. Development and evaluation of a scale to measure perceived self-efficacy in people with arthritis. *Arthritis Rheum* 1989;32(1):37-44. doi: 10.1002/anr.1780320107.

45. Self-Management Resource Center. English Evaluation Tools: Arthritis Self-Efficacy 2020. https://www.selfmanagementresource.com/resources/evaluation-tools/english-evaluation-tools (accessed 2nd April 2020).

46. Brady TJ. Measures of self-efficacy: Arthritis Self-Efficacy Scale (ASES), Arthritis Self-Efficacy Scale-8 Item (ASES-8), Children's Arthritis Self-Efficacy Scale (CASE), Chronic Disease Self-Efficacy Scale (CDSES), Parent's Arthritis Self-Efficacy Scale (PASE), and Rheumatoid Arthritis Self-Efficacy Scale (RASE). *Arthritis Care Res (Hoboken)* 2011;63 Suppl 11:S473-85. doi: 10.1002/acr.20567.

47. Zigmond AS, Snaith RP. The hospital anxiety and depression scale. *Acta Psychiatr Scand* 1983;67(6):361-70. doi: 10.1111/j.1600-0447.1983.tb09716.x.

48. Herdman M, Gudex C, Lloyd A, et al. Development and preliminary testing of the new five-level version of EQ-5D (EQ-5D-5L). *Qual Life Res* 2011;20(10):1727-36. doi: 10.1007/s11136-011-9903-x.

49. van Hout B, Janssen MF, Feng YS et al. Interim scoring for the EQ-5D-5L: mapping the EQ-5D-5L to EQ-5D-3L value sets. *Value Health* 2012;15(5):708-15. doi: 10.1016/j.jval.2012.02.008.

For peer review only



For peer review only - http://bmjopen.bmj.com/site/about/guidelines.xhtml

50. Beecham J, Knapp M. Costing psychiatric interventions. In: Thornicroft G, ed. Measuring Mental Health Needs. London, United Kingdom: Gaskell 2001:200-24.

51. D'Agostino RB, Belanger A, D'Agostino RB. A Suggestion for Using Powerful and Informative Tests of Normality. *Am Stat* 1990;44(4):316-21. doi: 10.1080/00031305.1990.10475751.

52. Koenker R, Hallock KF. Quantile Regression *J Econ Perspect* 2001;15(4):143-56. doi: 10.1257/jep.15.4.143.

53. Williams EM, Hyer JM, Viswanathan R, et al. Peer-to-Peer Mentoring for African American Women With Lupus: A Feasibility Pilot. *Arthritis Care Res (Hoboken)* 2018;70(6):908-17. doi: 10.1002/acr.23412.

54. Walshe C, Roberts D, Calman L, et al. Peer Mentors for People with Advanced Cancer: Lessons Learnt from Recruiting and Training Peer Mentors for a Feasibility Randomized Controlled Trial. *J Cancer Educ* 2020 doi: 10.1007/s13187-020-01692-7.

55. Kroon FPB, van der Burg LRA, Buchbinder R, et al. Self-management education programmes for osteoarthritis. *Cochrane Database of Syst Rev* 2014(1) doi: 10.1002/14651858.CD008963.pub2.

56. Lorig KR, Ritter P, Stewart AL, et al. Chronic disease self-management program: 2-year health status and health care utilization outcomes. *Med Care*. 2001 Nov;39(11):1217-23. doi: 10.1097/00005650-200111000-00008.

57. Dale J, Caramlau I, Sturt J, et al. Telephone peer-delivered intervention for diabetes motivation and support: The telecare exploratory RCT. *Patient Educ Couns* 2009;75(1):91-8. doi: 10.1016/j.pec.2008.09.014.

For peer review only



For peer review only - http://bmjopen.bmj.com/site/about/guidelines.xhtml

58. Sokol R, Fisher E. Peer Support for the Hardly Reached: A Systematic Review. *Am J Public Health* 2016;106(7):e1-e8. doi: 10.2105/AJPH.2016.303180.

59. Higgins JPT, Savović J, Page MJ, et al. Revised Cochrane risk-of-bias tool for randomized trials (RoB 2) 2019. https://sites.google.com/site/riskofbiastool/welcome/rob-2-0-tool/current-version-of-rob-2 (accessed 9th March 2020).

60. Michie S, Richardson M, Johnston M, et al. The Behavior Change Technique Taxonomy (v1) of 93 hierarchically clustered techniques: building an international consensus for the reporting of behavior change interventions. *Ann Behav Med* 2013 Aug;46(1):81-95. doi: 10.1007/s12160-013-9486-6.

## FIGURE LEGENDS

**Figure 1: Osteoarthritis peer mentorship intervention logic model**

OA, osteoarthritis.

[a] Behaviour change techniques are coded using the Behaviour Change Technique Taxonomy version 1.[60]

**Figure 2: Participant flow diagram**

[a] Minimum number of individuals approached/excluded. Additional individuals may have been approached/excluded because the number of individuals approached through physiotherapy clinics was not recorded to minimise the administrative burden on physiotherapists.



For peer review only - http://bmjopen.bmj.com/site/about/guidelines.xhtml

939 [b] Intervention completion was defined as completion of at least three peer mentorship sessions.

For peer review only



For peer review only - http://bmjopen.bmj.com/site/about/guidelines.xhtml

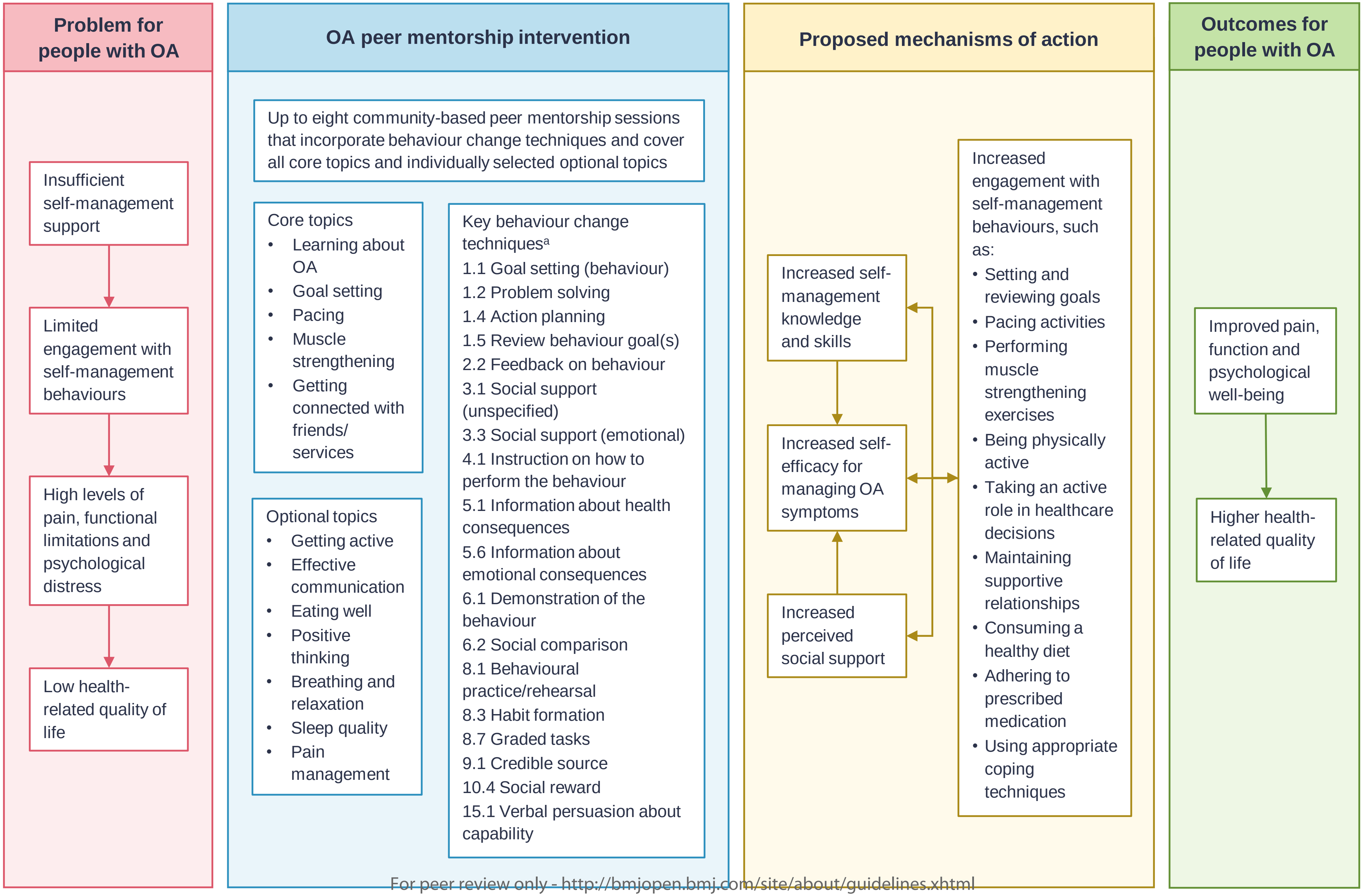
Problem for people with OA
Insufficient self-management support
Limited engagement with self-management behaviours
High levels of pain, functional limitations and psychological distress
Low health-related quality of life
OA peer mentorship intervention
Up to eight community-based peer mentorship sessions that incorporate behaviour change techniques and cover all core topics and individually selected optional topics
Core topics
• Learning about OA
• Goal setting
• Pacing
• Muscle strengthening
• Getting connected with friends/ services
Optional topics
• Getting active
• Effective communication
• Eating well
• Positive thinking
• Breathing and relaxation
• Sleep quality
• Pain management
Key behaviour change techniques[a]
1.1 Goal setting (behaviour)
1.2 Problem solving
1.4 Action planning
1.5 Review behaviour goal(s)
2.2 Feedback on behaviour
3.1 Social support (unspecified)
3.3 Social support (emotional)
4.1 Instruction on how to perform the behaviour
5.1 Information about health consequences
5.6 Information about emotional consequences
6.1 Demonstration of the behaviour
6.2 Social comparison
8.1 Behavioural practice/rehearsal
8.3 Habit formation
8.7 Graded tasks
9.1 Credible source
10.4 Social reward
15.1 Verbal persuasion about capability
Proposed mechanisms of action
Increased self-management knowledge and skills
Increased self-efficacy for managing OA symptoms
Increased perceived social support
Increased engagement with self-management behaviours, such as:
• Setting and reviewing goals
• Pacing activities
• Performing muscle strengthening exercises
• Being physically active
• Taking an active role in healthcare decisions
• Maintaining supportive relationships
• Consuming a healthy diet
• Adhering to prescribed medication
• Using appropriate coping techniques
Outcomes for people with OA
Improved pain, function and psychological well-being
Higher health-related quality of life

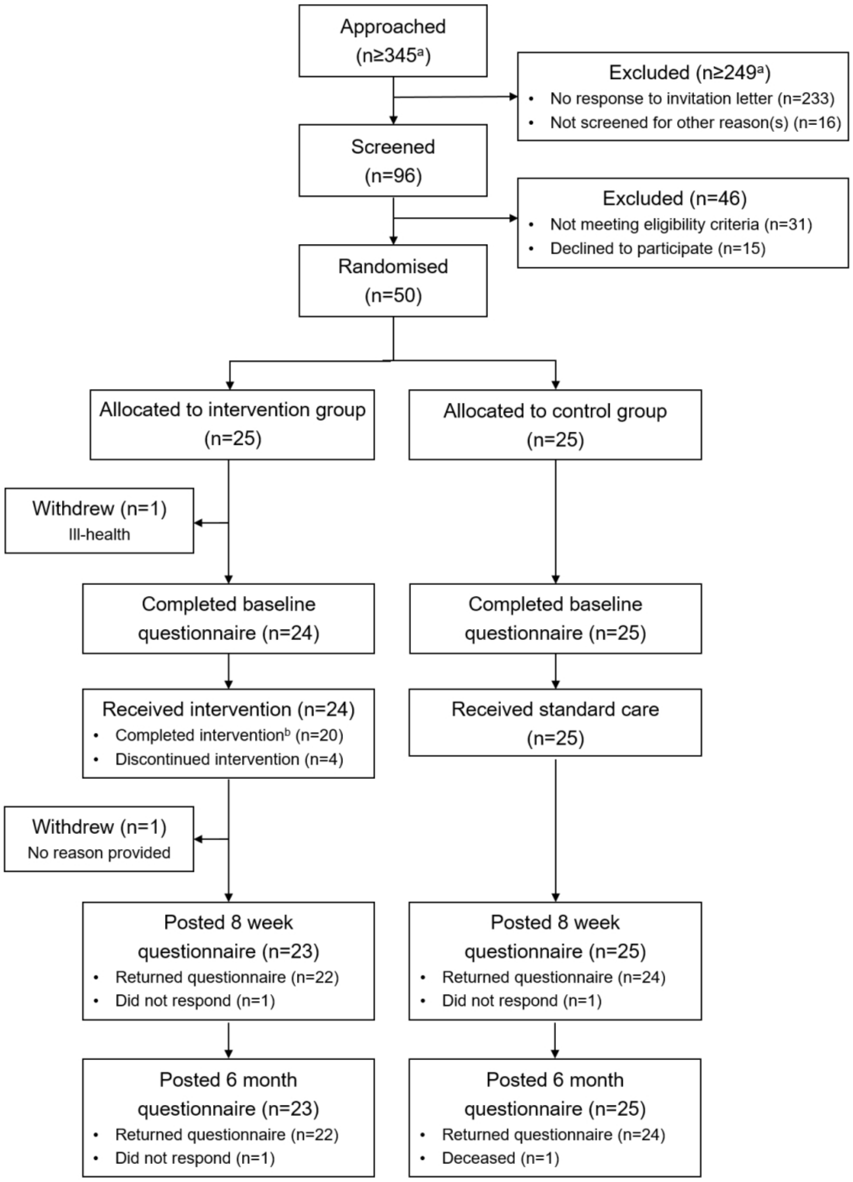


[a] Minimum number of individuals approached/excluded. Additional individuals may have been approached/excluded because the number of individuals approached through physiotherapy clinics was not recorded to minimise the administrative burden on physiotherapists.
[b] Intervention completion was defined as completion of at least three peer mentorship sessions.

214x295mm (150 x 150 DPI)

# Peer mentorship to improve self-management of osteoarthritis: A randomised feasibility trial – Supplementary Material

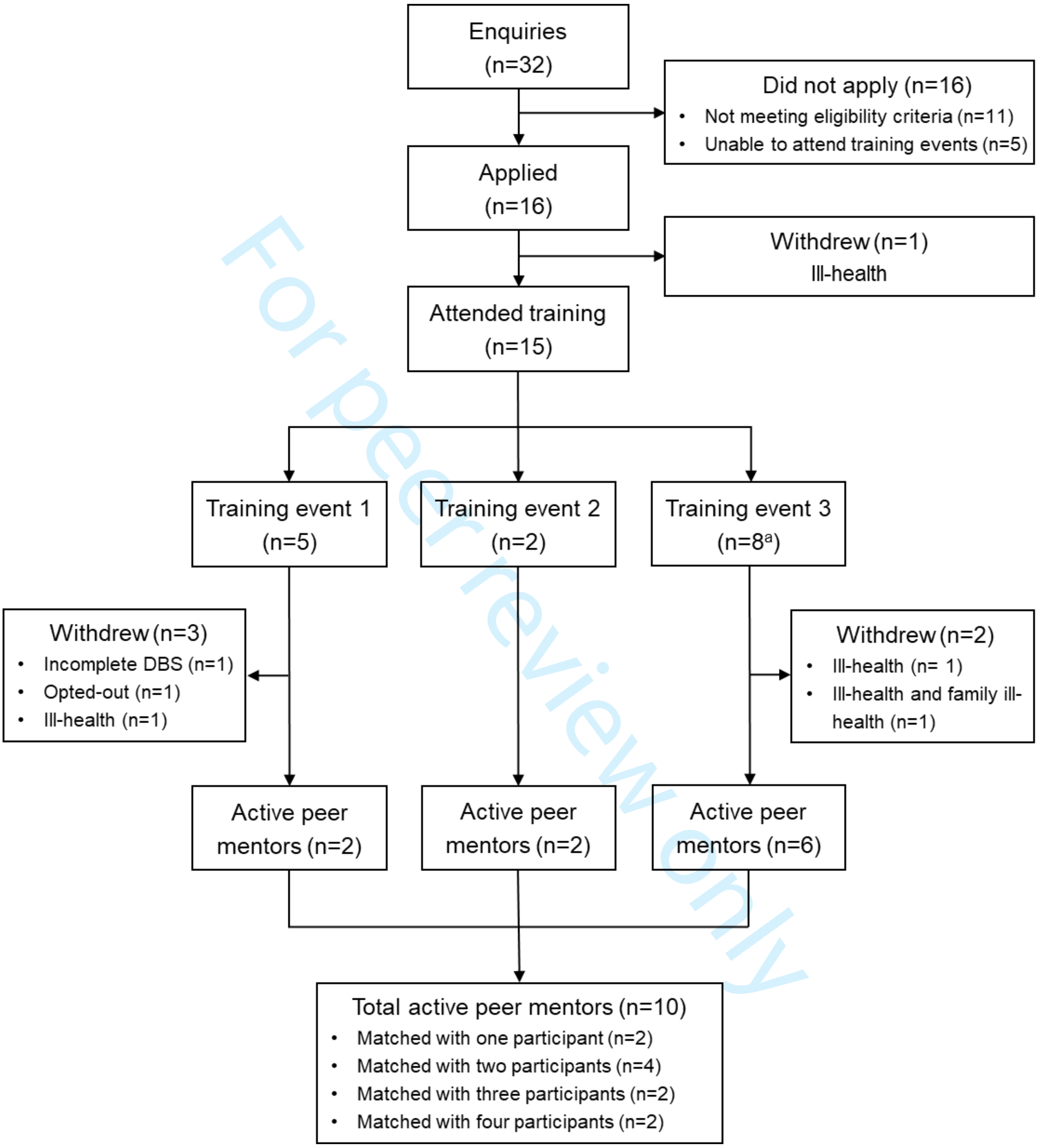


**Supplementary Figure 1: Peer mentor flow diagram**

[a] One trained peer mentor who attended Training event 1 also attended Training event 3 as refresher training.

DBS, Enhanced Disclosure and Barring Service check



For peer review only - http://bmjopen.bmj.com/site/about/guidelines.xhtml

**Supplementary Table 1: CONSORT 2010 checklist of information to include when reporting a pilot or feasibility randomised trial in a journal or conference abstract**

| Item | Description | Reported on line number |
|---|---|---|
| Title | Identification of study as randomised pilot or feasibility trial | 2 |
| Authors | Contact details for the corresponding author | 15-17 |
| Trial design | Description of pilot trial design (eg, parallel, cluster) | 28 |
| **Methods** | | |
| Participants | Eligibility criteria for participants and the settings where the pilot trial was conducted | 29-30, 33 |
| Interventions | Interventions intended for each group | 32-35 |
| Objective | Specific objectives of the pilot trial | 25-27 |
| Outcome | Prespecified assessment or measurement to address the pilot trial objectives | 36-40 |
| Randomisation | How participants were allocated to interventions | 32 |
| Blinding (masking) | Whether or not participants, care givers, and those assessing the outcomes were blinded to group assignment | 28 |
| **Results** | | |
| Numbers randomised | Number of participants screened and randomised to each group for the pilot trial objectives | 41-42 |
| Recruitment | Trial status | N/A[a] |
| Numbers analysed | Number of participants analysed in each group for the pilot objectives | 42-45 |
| Outcome | Results for the pilot objectives, including any expressions of uncertainty | 41-48 |
| Harms | Important adverse events or side effects | 45-46 |
| Conclusions | General interpretation of the results of pilot trial and their implications for the future definitive trial | 49-52 |
| Trial registration | Registration number for pilot trial and name of trial register | 53 |
| Funding | Source of funding for pilot trial | 54 |



For peer review only - http://bmjopen.bmj.com/site/about/guidelines.xhtml

N/A, Not applicable

[a] Not applicable as item is only applicable to conference abstracts.

Checklist from: Eldridge SM, Chan CL, Campbell MJ, et al. CONSORT 2010 statement: extension to randomised pilot and feasibility trials. *BMJ* 2016;355:i5239.

For peer review only

## Supplementary Table 2: CONSORT 2010 checklist of information to include when reporting a pilot or feasibility trial

| Section/Topic | Item No | Checklist item | Reported on page No |
|---|---|---|---|
| **Title and abstract** | | | |
| | 1a | Identification as a pilot or feasibility randomised trial in the title | 1 |
| | 1b | Structured summary of pilot trial design, methods, results, and conclusions | 2-3 |
| **Introduction** | | | |
| Background and objectives | 2a | Scientific background and explanation of rationale for future definitive trial, and reasons for randomised pilot trial | 4-5 |
| | 2b | Specific objectives or research questions for pilot trial | 5 |
| **Methods** | | | |
| Trial design | 3a | Description of pilot trial design (such as parallel, factorial) including allocation ratio | 5 |
| | 3b | Important changes to methods after pilot trial commencement (such as eligibility criteria), with reasons | 13 |
| Participants | 4a | Eligibility criteria for participants | 6 |
| | 4b | Settings and locations where the data were collected | 6, 11, 15 |
| | 4c | How participants were identified and consented | 6 |
| Interventions | 5 | The interventions for each group with sufficient details to allow replication, including how and when they were actually administered | 7-13<br>Figure 1<br>Supplementary table 3 |



For peer review only - http://bmjopen.bmj.com/site/about/guidelines.xhtml

For peer review only

Supplementary Material

| | | | |
|---|---|---|---|
| Outcomes | 6a | Completely defined prespecified assessments or measurements to address each pilot trial objective specified in 2b, including how and when they were assessed | 13-17 |
| | 6b | Any changes to pilot trial assessments or measurements after the pilot trial commenced, with reasons | N/A |
| | 6c | If applicable, prespecified criteria used to judge whether, or how, to proceed with future definitive trial | 14 |
| Sample size | 7a | Rationale for numbers in the pilot trial | 7 |
| | 7b | When applicable, explanation of any interim analyses and stopping guidelines | N/A |
| Randomisation: | | | |
| Sequence generation | 8a | Method used to generate the random allocation sequence | 7 |
| | 8b | Type of randomisation(s); details of any restriction (such as blocking and block size) | 7 |
| Allocation concealment mechanism | 9 | Mechanism used to implement the random allocation sequence (such as sequentially numbered containers), describing any steps taken to conceal the sequence until interventions were assigned | 7 |
| Implementation | 10 | Who generated the random allocation sequence, who enrolled participants, and who assigned participants to interventions | 6-7 |
| Blinding | 11a | If done, who was blinded after assignment to interventions (for example, participants, care providers, those assessing outcomes) and how | N/A |
| | 11b | If relevant, description of the similarity of interventions | N/A |
| Statistical methods | 12 | Methods used to address each pilot trial objective whether qualitative or quantitative | 14, 17-18 |
| **Results** | | | |

For peer review only



For peer review only - http://bmjopen.bmj.com/site/about/guidelines.xhtml

Supplementary Material

| | | | |
|---|---|---|---|
| Participant flow (a diagram is strongly recommended) | 13a | For each group, the numbers of participants who were approached and/or assessed for eligibility, randomly assigned, received intended treatment, and were assessed for each objective | 19-28<br>Figure 2 |
| | 13b | For each group, losses and exclusions after randomisation, together with reasons | 20<br>Figure 2 |
| Recruitment | 14a | Dates defining the periods of recruitment and follow-up | 5, 19 |
| | 14b | Why the pilot trial ended or was stopped | N/A |
| Baseline data | 15 | A table showing baseline demographic and clinical characteristics for each group | 23<br>Supplementary table 7 |
| Numbers analysed | 16 | For each objective, number of participants (denominator) included in each analysis. If relevant, these numbers should be by randomised group | 19-28 |
| Outcomes and estimation | 17 | For each objective, results including expressions of uncertainty (such as 95% confidence interval) for any estimates. If relevant, these results should be by randomised group | 19-28 |
| Ancillary analyses | 18 | Results of any other analyses performed that could be used to inform the future definitive trial | N/A |
| Harms | 19 | All important harms or unintended effects in each group | N/A |
| | 19a | If relevant, other important unintended consequences | N/A |
| **Discussion** | | | |
| Limitations | 20 | Pilot trial limitations, addressing sources of potential bias and remaining uncertainty about feasibility | 29-33 |
| Generalisability | 21 | Generalisability (applicability) of pilot trial methods and findings to future definitive trial and other studies | 32 |



For peer review only

For peer review only - http://bmjopen.bmj.com/site/about/guidelines.xhtml

| | | | |
|---|---|---|---|
| Interpretation | 22 | Interpretation consistent with pilot trial objectives and findings, balancing potential benefits and harms, and considering other relevant evidence | 28-33 |
| | 22a | Implications for progression from pilot to future definitive trial, including any proposed amendments | 29-30, 32-33 |
| **Other information** | | | |
| Registration | 23 | Registration number for pilot trial and name of trial registry | 5 |
| Protocol | 24 | Where the pilot trial protocol can be accessed, if available | 35 |
| Funding | 25 | Sources of funding and other support (such as supply of drugs), role of funders | 34 |
| | 26 | Ethical approval or approval by research review committee, confirmed with reference number | 5, 34 |

Checklist from: Eldridge SM, Chan CL, Campbell MJ, et al. CONSORT 2010 statement: extension to randomised pilot and feasibility trials. *BMJ* 2016;355:i5239.

For peer review only

**Supplementary Table 3: Key behaviour change techniques included in the osteoarthritis peer mentorship intervention**

| Behaviour change technique[a] | Example of implementation |
|---|---|
| 1.1 Goal setting (behaviour) | The peer mentor supports the participant to set a goal of meeting a friend for coffee twice weekly. |
| 1.2 Problem solving | The peer mentor supports the participant to analyse barriers to pacing their activities and identify strategies to help overcome the barriers. |
| 1.4 Action planning | The peer mentor supports the participant to plan when and where they will go cycling and how long their cycle rides will last. |
| 1.5 Review behaviour goal(s) | The peer mentor and participant review the participant's goal of not drinking any caffeinated drinks after 5.00pm and adapt it if necessary. |
| 2.2 Feedback on behaviour | The peer mentor provides the participant with feedback on their strengthening exercise technique during their peer mentorship session. |
| 3.1 Social support (unspecified) | The peer mentor provides information about a local walking football group. |
| 3.3 Social support (emotional) | The peer mentor provides emotional support to help the participant to go for a walk even though they feel anxious about falling. |
| 4.1 Instruction on how to perform the behaviour | The peer mentor advises the participant how to perform a relaxation technique. |
| 5.1 Information about health consequences | The peer mentor explains that losing weight can help improve symptoms of osteoarthritis. |
| 5.6 Information about emotional consequences | The peer mentor explains that using conscious breathing techniques can help people feel calmer. |
| 6.1 Demonstration of the behaviour | The peer mentor demonstrates how to perform chair based exercises. |
| 6.2 Social comparison | The peer mentor draws attention to how they are able to do yoga despite their osteoarthritis. |
| 8.1 Behavioural practice/rehearsal | The peer mentor prompts the participant to practice being assertive in preparation for their doctor's appointment. |
| 8.3 Habit formation | The peer mentor prompts the participant to carry out an exercise routine in their bedroom after they have had a shower each morning. |



For peer review only - http://bmjopen.bmj.com/site/about/guidelines.xhtml

| | |
|---|---|
| 8.7 Graded tasks | The peer mentor supports the participant to swim 20 lengths by advising them to start with 10 lengths and then add an extra two lengths each week. |
| 9.1 Credible source | The peer mentor provides communication in favour of eating five portions of fruit and vegetables per day. |
| 10.4 Social reward | The peer mentor praises the participant for progressing towards their goal of getting up at 8.30am each morning. |
| 15.1 Verbal persuasion about capability | The peer mentor tells the participant that they can do leg strengthening exercises safely despite their osteoarthritis. |

[a] Behaviour change techniques are coded using the Behaviour Change Technique Taxonomy version 1 from: Michie S, Richardson M, Johnston M, et al. The Behavior Change Technique Taxonomy (v1) of 93 hierarchically clustered techniques: building an international consensus for the reporting of behavior change interventions. *Ann Behav Med* 2013 Aug;46(1):81-95. doi: 10.1007/s12160-013-9486-6.

Only key behaviour change techniques are listed. Peer mentors could incorporate additional behaviour change techniques as appropriate to the participant's individual needs and goals.

For peer review only



For peer review only - http://bmjopen.bmj.com/site/about/guidelines.xhtml

## Supplementary Table 4: Overview of areas covered during the peer mentor training event

| Day 1 | Day 2 |
|---|---|
| • Overview of the trial and peer mentor role<br>• Osteoarthritis key facts and self-management topics<br>• Goal setting<br>• Pacing<br>• Educational resource pack review<br>• Exercise<br>• Pain management<br>• Sleep | • Review of day 1<br>• Communication<br>• Professional boundaries<br>• Mentoring in practice (including safeguarding, disclosures and confidentiality)<br>• Visit and recording procedures<br>• Trial data collection<br>• Next steps |

For peer review only



For peer review only - http://bmjopen.bmj.com/site/about/guidelines.xhtml

**Supplementary Table 5: Estimated costs of delivering the two day peer mentor training event to five peer mentors**

| **Staff costs**[a] | | | |
|---|---|---|---|
| | Hourly rate[b] | Hours per event | Cost per event |
| Research Fellow (Grade 7) | £32.23 | 7.5 | £241.73 |
| Research Associate (Grade 6) | £26.09 | 15 | £391.35 |
| Volunteer Coordinator (Grade 5) | £21.79 | 15 | £326.85 |
| Total staff costs | | | £959.93 |
| **Peer mentor costs** | | | |
| | Cost per peer mentor | | Cost per event |
| Refreshments | £18.00 | | £90.00 |
| Notebook | £2.48 | | £12.40 |
| Ring binder | £2.00 | | £10.00 |
| Plastic wallets | £0.67 | | £3.33 |
| Printing | £10.50 | | £52.50 |
| Name badge | £0.14 | | £0.68 |
| Travel expenses | £13.14 | | £65.70 |
| Total peer mentor costs | £46.92 | | £234.62 |
| **Overall costs** | | | |
| | Cost per peer mentor | | Cost per event |
| Overall costs | £238.91 | | £1,194.54 |

[a] Staff costs were based on the events run by the trial team members.

[b] Includes staff salary, National Insurance contributions and pension contributions, but does not include estates costs or indirect costs.

For peer review only

**Supplementary Table 6: Estimated costs of delivering the OA peer mentorship intervention to one participant**

| | | |
|---|---|---|
| **Volunteer Coordinator costs (Grade 5 hourly rate £21.79[a])** | | |
| | Per single session | Per 5.79 sessions[b] |
| Hours spent supporting mentorship sessions (0.5 hours per session) | £10.90 | £63.10 |
| Additional hours spent on matching, initial session and closure (4 hours) | £15.05 | £87.16 |
| Travel expenses for initial session | £0.81 | £4.72 |
| Total Volunteer Coordinator costs | £26.76 | £154.98 |
| **Peer mentor costs** | | |
| | Per single session | Per 5.79 sessions[b] |
| Honorarium | £15 | £86.88 |
| Travel expenses | £5.53 | £32.01 |
| Total peer mentor costs | £20.53 | £118.89 |
| **Overall costs** | | |
| | Per single session | Per 5.79 sessions[b] |
| Overall costs | £47.29 | £273.86 |

[a] Includes staff salary, National Insurance contributions and pension contributions, but does not include estates costs or indirect costs.

[b] 5.79 was the mean number of mentorship sessions received by participants.

For peer review only

## Supplementary Table 7: Additional participant baseline characteristics

| Characteristics | Intervention n=24 | Control n=25 |
|---|---|---|
| **Marital status,** n (%) | | |
| Married | 12 (50.0) | 17 (68.0) |
| Divorced | 6 (25.0) | 2 (8.0) |
| Living together | 0 (0.0) | 1 (4.0) |
| Widowed | 5 (20.8) | 3 (12.0) |
| Single | 1 (4.2) | 2 (8.0) |
| **Current smokers,** n (%) | 1 (4.2) | 2 (8.0) |
| **Areas affected by arthritis**, n (%) | | |
| Single hip | 0 (0.0) | 0 (0.0) |
| Both hips | 2 (8.3) | 4 (16.0) |
| Single knee | 2 (8.3) | 3 (12.0) |
| Both knees | 8 (33.3) | 9 (36.0) |
| Single hip and single knee | 2 (8.3) | 3 (12.0) |
| Both hips and one knee | 2 (8.3) | 1 (4.0) |
| Single hip and both knees | 5 (20.8) | 2 (8.0) |
| Both hips and both knees | 3 (12.5) | 3 (12.0) |
| **Areas affected by joint pain for > 6 weeks in the last 3 months**, n (%) | | |
| Neck | 7 (29.2) | 6 (24.0) |
| Shoulder (right) | 7 (29.2) | 6 (24.0) |
| Shoulder (left) | 7 (29.2) | 7 (28.0) |
| Elbow (right) | 0 (0.0) | 2 (8.0) |
| Elbow (left) | 0 (0.0) | 2 (8.0) |
| Hand (right) | 9 (37.5) | 6 (24.0) |
| Hand (left) | 9 (37.5) | 5 (20.0) |
| Back | 6 (25.0) | 11 (44.0) |
| Hip (right) | 10 (41.7) | 10 (40.0) |
| Hip (left) | 10 (41.7) | 10 (40.0) |
| Knee (right) | 19 (79.2) | 16 (64.0) |
| Knee (left) | 19 (79.2) | 20 (80.0) |



For peer review only

| | | |
|---|---|---|
| Foot (right) | 5 (20.8) | 4 (16.0) |
| Foot (left) | 4 (16.7) | 5 (16.0) |
| **Duration of pain in years,** mean (SD) | 4.7 (5.2) | 5.3 (6.2) |
| **Currently suffering from pain**, n (%) | 23 (95.8) | 25 (100.0) |
| **Frequency of pain**[a], n (%) | | |
| Always | 7 (30.4) | 11 (44.0) |
| Often present | 5 (21.7) | 9 (36.0) |
| Sometimes present | 10 (43.5) | 3 (20.0) |
| Present very little | 1 (4.4) | 0 (0.0) |
| **Previous hip replacement,** n (%) | 1 (4.2) | 1 (4) |
| **Previous knee replacement(s),** n (%) | 1 (4.2) | 2 (8) |
| **Walking aid use,** n (%) | 8 (33.3) | 7 (28.0) |
| **Currently using medication for pain or other symptoms of arthritis,** n (%) | 20 (83.3) | 21 (84.0) |
| **Currently using strong opioids,** n, (%) | 1 (4.2) | 0 (0.0) |
| **Currently using over-the-counter NSAIDS,** n (%) | 4 (16.7) | 6 (24.0) |

NSAIDS, non-steroidal anti-inflammatory drugs; SD, standard deviation.

[a] Frequency of pain intervention group n=23 because one participant reported they were not currently suffering from pain.



For peer review only - http://bmjopen.bmj.com/site/about/guidelines.xhtml

For peer review only

**Supplementary Table 8: Mean resource use and mean difference between groups for the 8 week and 6 month questionnaires combined**

| | Intervention group mean use (n=22) | Control group mean use (n=24) | Mean difference (95% Confidence Interval) |
|---|---|---|---|
| Inpatient length of stay (nights) | 0.05 | 0.96 | -0.91 (-1.78 to -0.04) |
| Number of Outpatient appointments | 3.05 | 2.92 | 0.13 (-1.87 to 2.13) |
| Number GP appointments (surgery) | 2.73 | 2.42 | 0.31 (-1.47 to 2.09) |
| Number of GP visits (home) | 0.00 | 0.17 | -0.17 (-0.52 to 0.18) |
| Number of GP calls (telephone) | 0.59 | 0.67 | -0.08 (-0.70 to 0.55) |
| Number of GP Practice Nurse visits | 1.64 | 0.71 | 0.93 (0.15 to 1.71) |
| Number of District Nurse visits | 0.00 | 0.00 | 0.00 |
| Number of Occupational Therapist visits | 0.09 | 0.21 | -0.12 (-0.47 to 0.24) |
| Number of Physiotherapist visits | 1.36 | 1.96 | -0.59 (-2.74 to 1.55) |
| Number of Chiropodist visits | 0.64 | 0.75 | -0.11 (-0.86 to 0.63) |
| Number of Pharmacist visits | 2.27 | 1.67 | 0.61 (-1.04 to 2.25) |
| Number of NHS Direct calls | 0.18 | 0.13 | 0.06 (-0.28 to 0.39) |
| Number of Walk-in Centre visits | 0.41 | 0.13 | 0.28 (-0.43 to 1.00) |
| Number of Counsellor visits | 0.09 | 0.08 | 0.01 (-0.24 to 0.26) |

For peer review only



For peer review only - http://bmjopen.bmj.com/site/about/guidelines.xhtml

# Peer mentorship to improve self-management of osteoarthritis: A randomised feasibility trial – CONSORT checklists

## CONSORT 2010 checklist of information to include when reporting a pilot or feasibility randomised trial in a journal or conference abstract

| Item | Description | Reported on line number |
|---|---|---|
| Title | Identification of study as randomised pilot or feasibility trial | 2 |
| Authors | Contact details for the corresponding author | 15-17 |
| Trial design | Description of pilot trial design (eg, parallel, cluster) | 28 |
| **Methods** | | |
| Participants | Eligibility criteria for participants and the settings where the pilot trial was conducted | 29-30, 33 |
| Interventions | Interventions intended for each group | 32-35 |
| Objective | Specific objectives of the pilot trial | 25-27 |
| Outcome | Prespecified assessment or measurement to address the pilot trial objectives | 36-40 |
| Randomisation | How participants were allocated to interventions | 32 |
| Blinding (masking) | Whether or not participants, care givers, and those assessing the outcomes were blinded to group assignment | 28 |
| **Results** | | |
| Numbers randomised | Number of participants screened and randomised to each group for the pilot trial objectives | 41-42 |
| Recruitment | Trial status | N/A[a] |
| Numbers analysed | Number of participants analysed in each group for the pilot objectives | 42-45 |
| Outcome | Results for the pilot objectives, including any expressions of uncertainty | 41-48 |
| Harms | Important adverse events or side effects | 45-46 |
| Conclusions | General interpretation of the results of pilot trial and their implications for the future definitive trial | 49-52 |
| Trial registration | Registration number for pilot trial and name of trial register | 53 |



For peer review only - http://bmjopen.bmj.com/site/about/guidelines.xhtml

CONSORT checklists

| | | |
|---|---|---|
| Funding | Source of funding for pilot trial | 54 |

N/A, Not applicable

[a] Not applicable as item is only applicable to conference abstracts.

Checklist from: Eldridge SM, Chan CL, Campbell MJ, et al. CONSORT 2010 statement: extension to randomised pilot and feasibility trials. *BMJ* 2016;355:i5239.

For peer review only



For peer review only - http://bmjopen.bmj.com/site/about/guidelines.xhtml

CONSORT checklists

## CONSORT 2010 checklist of information to include when reporting a pilot or feasibility trial

| Section/Topic | Item No | Checklist item | Reported on page No |
|---|---|---|---|
| **Title and abstract** | | | |
| | 1a | Identification as a pilot or feasibility randomised trial in the title | 1 |
| | 1b | Structured summary of pilot trial design, methods, results, and conclusions | 2-3 |
| **Introduction** | | | |
| Background and objectives | 2a | Scientific background and explanation of rationale for future definitive trial, and reasons for randomised pilot trial | 4-5 |
| | 2b | Specific objectives or research questions for pilot trial | 5 |
| **Methods** | | | |
| Trial design | 3a | Description of pilot trial design (such as parallel, factorial) including allocation ratio | 5 |
| | 3b | Important changes to methods after pilot trial commencement (such as eligibility criteria), with reasons | 13 |
| Participants | 4a | Eligibility criteria for participants | 6 |
| | 4b | Settings and locations where the data were collected | 6, 11, 15 |
| | 4c | How participants were identified and consented | 6 |
| Interventions | 5 | The interventions for each group with sufficient details to allow replication, including how and when they were actually administered | 7-13<br>Figure 1<br>Supplementary table 3 |



For peer review only - http://bmjopen.bmj.com/site/about/guidelines.xhtml

CONSORT checklists

| | | | |
|---|---|---|---|
| Outcomes | 6a | Completely defined prespecified assessments or measurements to address each pilot trial objective specified in 2b, including how and when they were assessed | 13-17 |
| | 6b | Any changes to pilot trial assessments or measurements after the pilot trial commenced, with reasons | N/A |
| | 6c | If applicable, prespecified criteria used to judge whether, or how, to proceed with future definitive trial | 14 |
| Sample size | 7a | Rationale for numbers in the pilot trial | 7 |
| | 7b | When applicable, explanation of any interim analyses and stopping guidelines | N/A |
| Randomisation: | | | |
| Sequence generation | 8a | Method used to generate the random allocation sequence | 7 |
| | 8b | Type of randomisation(s); details of any restriction (such as blocking and block size) | 7 |
| Allocation concealment mechanism | 9 | Mechanism used to implement the random allocation sequence (such as sequentially numbered containers), describing any steps taken to conceal the sequence until interventions were assigned | 7 |
| Implementation | 10 | Who generated the random allocation sequence, who enrolled participants, and who assigned participants to interventions | 6-7 |
| Blinding | 11a | If done, who was blinded after assignment to interventions (for example, participants, care providers, those assessing outcomes) and how | N/A |
| | 11b | If relevant, description of the similarity of interventions | N/A |
| Statistical methods | 12 | Methods used to address each pilot trial objective whether qualitative or quantitative | 14, 17-18 |
| **Results** | | | |

For peer review only



For peer review only - http://bmjopen.bmj.com/site/about/guidelines.xhtml

CONSORT checklists

| | | | |
|---|---|---|---|
| Participant flow (a diagram is strongly recommended) | 13a | For each group, the numbers of participants who were approached and/or assessed for eligibility, randomly assigned, received intended treatment, and were assessed for each objective | 19-28<br>Figure 2 |
| | 13b | For each group, losses and exclusions after randomisation, together with reasons | 20<br>Figure 2 |
| Recruitment | 14a | Dates defining the periods of recruitment and follow-up | 5, 19 |
| | 14b | Why the pilot trial ended or was stopped | N/A |
| Baseline data | 15 | A table showing baseline demographic and clinical characteristics for each group | 23<br>Supplementary table 7 |
| Numbers analysed | 16 | For each objective, number of participants (denominator) included in each analysis. If relevant, these numbers should be by randomised group | 19-28 |
| Outcomes and estimation | 17 | For each objective, results including expressions of uncertainty (such as 95% confidence interval) for any estimates. If relevant, these results should be by randomised group | 19-28 |
| Ancillary analyses | 18 | Results of any other analyses performed that could be used to inform the future definitive trial | N/A |
| Harms | 19 | All important harms or unintended effects in each group | N/A |
| | 19a | If relevant, other important unintended consequences | N/A |
| **Discussion** | | | |
| Limitations | 20 | Pilot trial limitations, addressing sources of potential bias and remaining uncertainty about feasibility | 29-33 |
| Generalisability | 21 | Generalisability (applicability) of pilot trial methods and findings to future definitive trial and other studies | 32 |



For peer review only - http://bmjopen.bmj.com/site/about/guidelines.xhtml

CONSORT checklists

| | | | |
|---|---|---|---|
| Interpretation | 22 | Interpretation consistent with pilot trial objectives and findings, balancing potential benefits and harms, and considering other relevant evidence | 28-33 |
| | 22a | Implications for progression from pilot to future definitive trial, including any proposed amendments | 29-30, 32-33 |
| **Other information** | | | |
| Registration | 23 | Registration number for pilot trial and name of trial registry | 5 |
| Protocol | 24 | Where the pilot trial protocol can be accessed, if available | 35 |
| Funding | 25 | Sources of funding and other support (such as supply of drugs), role of funders | 34 |
| | 26 | Ethical approval or approval by research review committee, confirmed with reference number | 5, 34 |

Checklist from: Eldridge SM, Chan CL, Campbell MJ, et al. CONSORT 2010 statement: extension to randomised pilot and feasibility trials. *BMJ* 2016;355:i5239.

For peer review only
